# Transcriptomic analysis of a classical model of carbon catabolite regulation in *Streptomyces coelicolor*

**DOI:** 10.1186/s12866-016-0690-y

**Published:** 2016-04-27

**Authors:** Alba Romero-Rodríguez, Diana Rocha, Beatriz Ruiz-Villafan, Víctor Tierrafría, Romina Rodríguez-Sanoja, Daniel Segura-González, Sergio Sánchez

**Affiliations:** Departamento de Biología Molecular y Biotecnología del Instituto de Investigaciones Biomédicas, Universidad Nacional Autónoma de México, Tercer Circuito Exterior s/n, Ciudad de Mexico, 04510 Mexico; Departamento de Microbiología Molecular, Instituto de Biotecnología, Universidad Nacional Autónoma de México, Ave. Universidad 2001, Cuernavaca, Mor. 62210 Mexico

**Keywords:** Transcriptome, Transcriptional factors, Carbon catabolite regulation, Glucose kinase, *Streptomyces coelicolor*

## Abstract

**Background:**

In the genus *Streptomyces*, one of the most remarkable control mechanisms of physiological processes is carbon catabolite repression (CCR). This mechanism regulates the expression of genes involved in the uptake and utilization of alternative carbon sources. CCR also affects the synthesis of secondary metabolites and morphological differentiation. Even when the outcome effect of CCR in different bacteria is the same, their essential mechanisms can be quite different. In several streptomycetes glucose kinase (Glk) represents the main glucose phosphorylating enzyme and has been regarded as a regulatory protein in CCR. To evaluate the paradigmatic model proposed for CCR in *Streptomyces*, a high-density microarray approach was applied to *Streptomyces coelicolor* M145, under repressed and non-repressed conditions. The transcriptomic study was extended to assess the ScGlk role in this model by comparing the transcriptomic profile of *S. coelicolor* M145 with that of a ∆glk mutant derived from the wild-type strain, complemented with a heterologous *glk* gene from *Zymomonas mobilis* (*Zmglk*), insensitive to CCR but able to grow in glucose (*ScoZm* strain).

**Results:**

Microarray experiments revealed that glucose influenced the expression of 651 genes. Interestingly, even when the ScGlk protein does not have DNA binding domains and the glycolytic flux was restored by a heterologous glucokinase, the ScGlk replacement modified the expression of 134 genes. From these, 91 were also affected by glucose while 43 appeared to be under the control of ScGlk. This work identified the expression of *S. coelicolor* genes involved in primary metabolism that were influenced by glucose and/or ScGlk. Aside from describing the metabolic pathways influenced by glucose and/or ScGlk, several unexplored transcriptional regulators involved in the CCR mechanism were disclosed.

**Conclusions:**

The transcriptome of a classical model of CCR was studied in *S. coelicolor* to differentiate between the effects due to glucose or ScGlk in this regulatory mechanism. Glucose elicited important metabolic and transcriptional changes in this microorganism. While its entry and flow through glycolysis and pentose phosphate pathway were stimulated, the gluconeogenesis was inhibited. Glucose also triggered the CCR by repressing transporter systems and the transcription of enzymes required for secondary carbon sources utilization. Our results confirm and update the agar model of the CCR in *Streptomyces* and its dependence on the ScGlk *per se*. Surprisingly, the expected regulatory function of ScGlk was not found to be as global as thought before (only 43 out of 779 genes were affected), although may be accompanied or coordinated by other transcriptional regulators. Aside from describing the metabolic pathways influenced by glucose and/or ScGlk, several unexplored transcriptional regulators involved in the CCR mechanism were disclosed. These findings offer new opportunities to study and understand the CCR in *S. coelicolor* by increasing the number of known glucose and ScGlk -regulated pathways and a new set of putative regulatory proteins possibly involved or controlling the CCR.

**Electronic supplementary material:**

The online version of this article (doi:10.1186/s12866-016-0690-y) contains supplementary material, which is available to authorized users.

## Background

Saprophytic soil bacteria from the genus *Streptomyces* produce a large number of secondary metabolites and extracellular enzymes [1–3]. Streptomycetes are ecologically important in carbon recycling and constitute the largest genus of Actinobacteria, a phylum including more than 900 species [4, 5].

In general, free-living bacteria must adapt to constantly changing environmental conditions. Therefore, they have developed mechanisms for finely modulate metabolism and growth. One of the most remarkable control mechanisms is carbon catabolite repression (CCR). This mechanism guarantees the sequential utilization of carbon sources when more than one is simultaneously present in the culture media. Even when the outcome effect of CCR in different bacteria is the same, their essential mechanisms can be quite different. In Gram-negative bacteria, CCR relies on the carbohydrate translocation phosphoenolpyruvate-dependent phosphotransferase system (PTS). For *Escherichia coli* and other Gram-negative bacteria, the main control system is cAMP-receptor protein (Crp), whose activity is related to the levels of the phosphorylated PTS enzyme EII^Glc^. On the other hand, in some low guanine-cytosine Gram-positive bacteria like *Bacillus subtilis*, the control is exerted by the catabolite control protein (CcpA), which is related to the phosphorylation levels of the PTS protein HPr-His [6].

In the *Streptomyces* genus (Gram-positive bacteria with high guanine-cytosine content), glucose also exerts an inhibitory effect on the expression of genes involved in the uptake and utilization of alternative carbon sources [7–10]. But, in contrast with other bacteria in *Streptomyces* glucose is not transported by the PTS system, but via the Major Facilitator System (MFS), GlcP [11, 12]. Besides, orthologous Crp (SCO3571) does not seem to play a significant role in CCR in these genera [13].

After the observation that mutants of *Streptomyces coelicolor* and other streptomycetes resistant to the non-utilizable glucose analog, 2-deoxyglucose (2-dog), lack glucose kinase (Glk) activity and lose sensitivity to CCR, a key role was ascribed to Glk in the CCR mechanism [10, 14, 15]. Furthermore, when Glk activity and consequently, the glycolytic flux is restored by transformation in the *S. coelicolor* mutant with a heterologous non-related Glk from *Zymomonas mobilis*, its growth in glucose is recovered, but not its sensitivity to CCR [16], suggesting a dual function of Glk. However, attempts to separate the phosphorylation activity from the regulatory function have failed [17].

Based on their primary structure, microbial Glks are classified into three families [18]. *S. coelicolor* Glk belongs to the family III, which contains a ROK signature (Repressor, ORF, Kinase). However, in contrast to the transcriptional repressors of this family, these kinases lack DNA binding domains. The only presumed interaction reported for Glk is with the glucose transporter Glcp [19].

Using a high-density microarray approach, in the present work the CCR paradigmatic model proposed by Angell et al. [16] for *S. coelicolor* was explored. For this purpose, the transcriptomic profile of a *glk* null mutant, complemented with a heterologous *glk* gene was analyzed and compared to the wild-type *SCO*M145 strain. In addition, this transcriptomic study was extended to evaluate the glucose effect under repressive and non-repressive conditions.

## Results and discussion

### Deletion of *glk*, growth and glucose utilization

For several years, studies to understand CCR and carbon regulation in *Streptomyces* have depended on the isolation of spontaneous mutants resistant to the glucose analog, 2-deoxyglucose [7, 15]. Although some of these mutations relied on the *glk* gene, the full effect of the mutagenic treatment remains unknown. Therefore, to isolate the “Glk” effect, the *glk* gene (*SCO2126*) was deleted in the *S. coelicolor* M145 wild-type generating the *Sco*∆*glk* strain. In addition, the *Z. mobilis glk* gen was cloned into the plasmid pIJ702, generating the plasmid pUNAMZm. The plasmids pIJ702 and pUNAMZm were independently transformed into the *Sco*∆*glk* mutant generating the *Sco*702 and *ScoZm* strains. A qualitative in vivo visualization of Glk activity using BPG Agar medium displayed a clear Glk activity in strains *Sco*M145 and *ScoZm* while *Sco*702 was unable to grow in this medium (Additional file 1: Figure S1). The capacity of *Z. mobilis* Glk to reestablish growth of the Δ*glk* mutant was quantitatively confirmed measuring the specific growth rate, μ = 0.08 h^−1^ for *Sco*M145 and μ = 0.09 h^−1^ for *ScoZm*. Next, the complemented mutant *ScoZm* was used in the transcriptomic assays.

### Global transcriptomic analysis

**Fig. 1:**
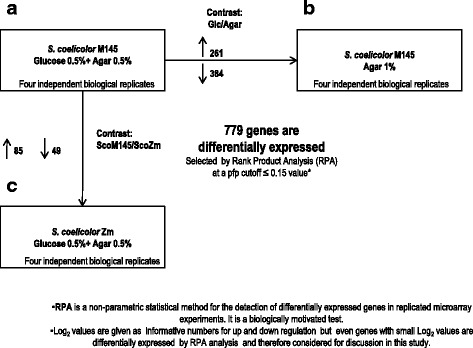
Scheme showing the number of differentially expressed genes in comparisons between strains and carbon sources. Orientation of the longer arrows shows the comparison between both conditions. Differential transcription values were obtained by Rank product analysis at a pfp cutoff ≤ 0.15 value. The small arrows up or down oriented indicate the number of genes up or down-expressed in each condition, respectively. **a** *Sco*M145 strain grown under repressive conditions. **b** *Sco*M145 strain grown under non-repressive conditions. **c** *Sco*Zm mutant grown under repressive conditions

As introduced above, even when CCR has been largely studied in *S. coelicolor*, neither the Glk protein role nor its precise regulatory mechanism has been elucidated. Therefore, to evaluate the effect of ScGlk in CCR, the gene expression of *Sco*M145 and the *ScoZm*, were compared under repressed conditions at the exponential growth phase, ([M145/*ScoZm*] in Fig. 1). Additionally, to evaluate the effect of glucose on gene expression, a global transcriptomic analysis (GTA) [20] was performed using a classical glucose-repressive condition over agar utilization, in exponential growing phase cultures using a classical glucose repressive condition over agar utilization [16]. Thus, the transcriptomic profile of *Sco*M145 was compared between repressive (referred in the text as Glc) (0.5 % agar plus 0.5 % glucose, and non-repressive conditions (referred as Agar) (0.5 % agar partially hydrolyzed) ([Glc/Agar] in Fig. 1). Additionally, to evaluate the ScGlk participation in CCR, the gene expression of *Sco*M145 and the *ScoZm*, were compared under repressive conditions at the exponential growth phase ([M145/*ScoZm*] in Fig. 1). GTA was applied to four independent biological replicates using the 104 K microarray from Oxford Gene Technology Ltd (UK).

**Fig. 2:**
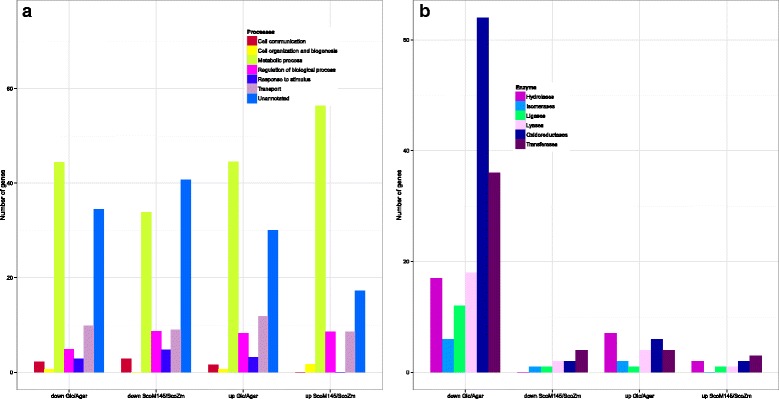
**a** Classification based on GO Biological Processes for Glk/Agar and M145/*ScoZm* comparisons. In all cases the most representatives GO are those corresponding to metabolic processes, transport and regulation of biological processes. **b** Classification of genes coding for enzymes included in the GO Metabolic processes

Average expression data were compared, using the Rank Products Analysis method (RPA). For all expression data, the RPA method calculated a pfp value. Those genes with a pfp value ≈ 0, have the highest probability of biological relevance [21]. Differentially expressed (up-and down-regulated) genes were identified as having a pfp value less than or equal to 0.15, equal to a false discovery rate of approximately 15 %, as previously reported [22]. This statistical analysis involving four biological replicates resulted in the identification of 261 up-regulated and 390 down-regulated genes in the Glc/Agar comparison and, 85 up-regulated and 96 down-regulated genes in the *Sco*M145/*ScoZm* comparison. In total, 785 genes were differentially expressed between both comparisons (Fig. 2).

The list of differentially expressed genes was submitted to the Protein Center (Thermo®) to group broadly them on the basis of its GO-Biological Function Term (GO-BP) (Fig. 2a). Half of the annotated genes were related to the GO metabolic processes in both comparisons. The GO term “metabolic process”, includes anabolic and catabolic chemical reactions and pathways. Looking closer inside the GO Metabolic process, genes encoding enzymes were also classified. A considerable number of oxidoreductases, hydrolases and transferases were repressed by glucose in Glc/Agar comparison (Fig. 2b). Comparison between *Sco*M145 and the *ScoZm* mutant revealed that the presence of a Glk protein, but not its enzyme activity, stimulated or repressed mostly the expression of oxidoreductases and transferases (Fig. 2b).

Considering their possible role in signal transduction, we have also looked for potential sensor kinases in this group. For the Glc/Agar comparison, regarding to agar, the sensor kinase gene *SCO6163* (log_2_ = −2.09) was down-regulated by glucose (Additional file 1: Table S1). In the *Sco*M14/*ScoZm* comparison, the putative sensor kinase gene *SCO6268* (log_2_ = 1.56) was up-regulated (Additional file 1: Table S2).

### Metabolic processes and catalysis

**Fig. 3:**
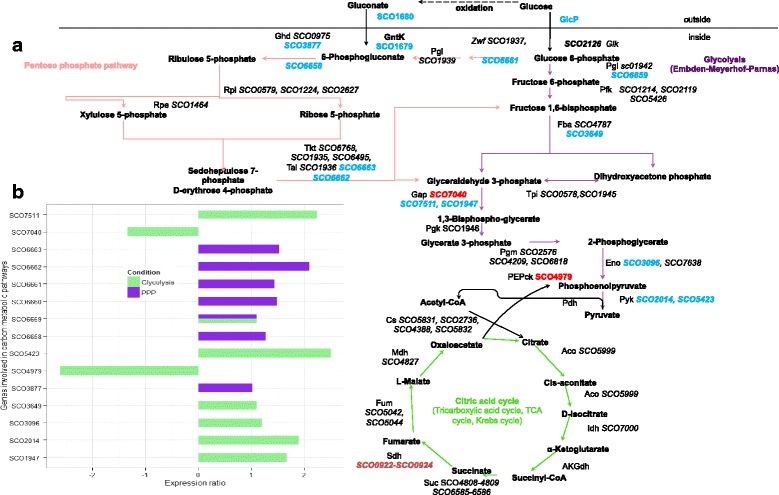
Profile of glucose dependent genes involved in both, glycolysis (*green bars*) and pentose phosphate pathways (*purple bars*). **a** Schematic representation of the central carbon metabolism pathways in *S. coelicolor*, in red are genes stimulated and in blue are genes repressed. **b** The comparison between conditions is indicated as ratios of Glc/Agar. Results are given as log2 ratios, thus a positive number indicates up-regulation and a negative number down-regulation, respectively

**Fig. 4:**
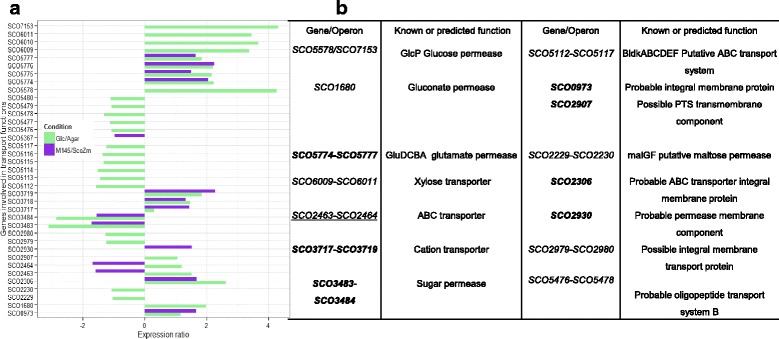
Selected genes encoding transporters and their expression profiles depending upon glucose and Glk presence. **a** Glc/Agar comparison reflects the glucose effect (*green bars*) and the M145/*ScoZm* comparison shows Glk dependence (*purple bars*). The comparison between conditions is indicated as ratio of Glc/Agar and *Sco*M145/*ScoZm*. Results are given as log2 ratio, thus a positive number indicates up-regulation and a negative down-regulation, respectively. **b** Putative products of regulated genes are shown. Genes that showed the same pattern of repression/stimulation across Glc/Agar and M145/*ScoZm* comparisons, are indicated in bold. Genes that exhibit an opposite pattern of stimulation or repression between comparisons are underlined

As previously mentioned glucose is mostly transported inside the cell by the GlcP transporter, most probably bound to ScGlk [19]. In this hypothesis, the glucose is transported and phosphorylated in a single step (Fig. 3a). In the Glc/Agar comparison, the genes *glcP1* (*SCO5578*), and *glcP2* (*SCO7153*) showed the highest stimulation exerted by glucose (20-fold increase, and ranked number 1 and 2, respectively) (Fig. 4a). The transcriptional regulation of *glcP* was dependent on the presence of glucose but ScGlk independent since in the *Sco*M145/*ScoZm* comparison no changes were detected for Glc transporters. Previous reports have demonstrated a constitutive expression of sc*glk* [19, 23]. In agreement with this result, we did not observe differential expression of sc*glk* in the Glc/Agar comparison. As expected, this difference was detected when comparing M145/*ScoZm* due to ScGlk deletion. No other glucokinases were found being regulated by glucose or ScGlk. Once glucose-6-phosphate is formed, it can enter to central carbon catabolic pathways (Fig. 3a).

#### Glycolysis and gluconeogenesis

Glycolysis or the Embden-Meyerhof-Parnas pathway (EMP) usually consists of ten reactions in which glucose is degraded to pyruvate (Fig. 3a). This pathway is present in all domains of life and hence is considered one of the most ancient pathways for sugar degradation. The expression of eight glycolytic steps involved in glucose degradation was up-regulated by glucose (Fig. 3b). This differential expression was not observed in the *Sco*M145/*ScoZm* comparison, suggesting that glycolysis proceeded more actively when glucose was present (compared to agar), but it was not affected by the absence of endogenous sc*glk* (*SCO2126*). In detail, two of the three isoforms of the glyceraldehyde-3-phosphate dehydrogenase genes (*SCO7511* and *SCO1947*) showed induction by glucose while *SCO7040* (glyceraldehyde-3-phosphate dehydrogenase) was glucose repressed. Expression of the *SCO4979* gene encoding the gluconeogenic phosphoenolpyruvate carboxykinasewas glucose repressed (log_2_ = −2.61) (Fig. 3b). The genes with greater changes were those encoding a pyruvate kinase isoform 2 (Pyk2) (*SCO5423*; log_2_ = 2.5) and phosphoenolpyruvate carboxykinase (PEPCK) (*SCO4979*; log_2_ = −2061), which were strongly induced and repressed by this carbohydrate, respectively (Fig. 3b). The large changes in the expression of these genes reflected their importance as regulatory points for the glucose/gluconeogenesis pathways. It is generally known that enzymes of metabolic pathways catalyzing irreversible steps are potential sites for regulatory controls. Pyk2 catalyzes the transfer of phosphate from phosphoenolpyruvate to ADP, producing pyruvate and ATP. This reaction has a large negative free energy change and represents the third regulated and irreversible enzyme of glycolysis.

The strong repression of glucose over the PEPCK gene whose product is involved in the conversion of oxalacetate to phosphoenolpyruvate, may reflect the strict control exerted by this carbohydrate, as this enzyme catalyzes the rate-controlling step of gluconeogenesis, and likely is the first committed step in all organisms [24, 25]. In *B. subtilis*, PEPCK is also reported to operate in reverse direction [25, 26] and might function in the anaplerotic node linking TCA cycle and glycolysis/gluconeogenesis.

#### The tricarboxylic acid cycle

The tricarboxylic acid cycle (TCA) is the final common pathway for molecules from which energy is generated. Molecules enter TCA as acetyl-CoA and leave as CO_2_. In the Glc/Agar comparison, just the putative succinate dehydrogenase complex encoded by *SCO0922*-*SCO0924* was induced, while in the tested conditions, the expression of other genes coding or enzymes of the TCA, remained unchanged, suggesting that the TCA cycle may be regulated mostly by the metabolic intermediates rather than by transcriptional regulation (Fig. 3a). Proteomic studies have previously suggested that the TCA enzymes are not regulated by protein expression [27] (Fig. 3a).

#### Pentose phosphate pathway

This pathway, also known as the phosphogluconate pathway or hexose monophosphate shunt, is another way to metabolize carbon sources like glucose, generating reducing power in the form of NADPH and C5 carbohydrates (Fig. 3a). The ribulose-5-phosphate is an important precursor of ATP, CoA, NAD^+^, FAD^+^, DNA and RNA production. In *S. coelicolor*, there are two major clusters encoding enzymes involved in the pentose phosphate pathway (PPP). In the first cluster, *SCO1935*-*SCO1942*, neither the presence of glucose nor the ScGlk have an effect on its expression level. The second cluster, *SCO6658*-*SCO6663* (encoding a 6-phosphogluconate dehydrogenase, glucose-6-phosphate isomerase, a hypothetical protein, glucose-6-phosphate 1-dehydrogenase, transaldolase and transketolase B, respectively) was entirely stimulated by glucose (Fig. 3b), and of these, the most noticeable change observed was in the *SCO6662* gene, encoding a transaldolase with a log_2_ = 2.11 (Fig. 3b). It has been proposed that this cluster may be involved in providing NADPH for secondary metabolite production [27, 28].

In concordance with the reported capacity of Glk from *Z. mobilis* to reestablish the glycolytic flux [16], no changes were observed in the transcriptomic data of glycolysis, PPP, TCA cycle or gluconeogenesis in the *Sco*M145/*ScoZm* comparison.

Therefore, regarding central carbon metabolism the glycolysis and PPP were highly activated only by glucose. Enzymes in the TCA cycle practically did not change, while gluconeogenesis was inhibited by glucose, supporting a preferential channeling of glucose to form essential building blocks. In this regard, a good correlation between our transcriptomic data and previously reported proteomics [27] data was observed.

Additionally, a putative gluconokinase (Glnk) coding gene (*SCO1679*) was also stimulated by glucose in the Glc/Agar comparison, while no changes were detected in the *Sco*M145/*ScoZm* comparison. The finding of up-regulation in the GlnK encoding gene *SCO1679* (log_2_ = 1.98), correlated well with a glucose positive regulation of *SCO1680* (log_2_ = 1.99), encoding a gluconate transporter (Fig. 3a). In *Streptomyces lividans*, the consumption of gluconate as a sole carbon source has been reported by [29]. However, to our knowledge, no studies on the regulation of the gluconate catabolic genes in *S. coelicolor* have been previously performed. In several genera of bacteria such as *Pseudomonas*, *Gluconobacter*, *Acetobacter*, and various fungi, gluconate is produced from glucose through a simple dehydrogenation reaction catalyzed by a glucose oxidase [30]. In general, bacterial growth on gluconate requires two specific enzymes: a gluconate permease (GntP) for extracellular gluconate incorporation and a gluconate kinase (GntK), which phosphorylates gluconate to 6-phosphogluconate [31]. In *Streptomyces*, gluconate can eventually be degraded by PPP to generate reducing power, needed for secondary metabolite biosynthesis [29]. In agreement with this observation, glucose activation of this PPP branch (Fig. 3a) was in synchrony with the over-expression of both *SCO3877* and *SCO6658* genes (gluconate transporter and gluconate kinase) (Fig. 3b).

### CCR at the operon-specific mechanisms

In many bacteria CCR may operate at operon-specific mechanisms by both a) inducer exclusion, i.e., prevention of the internalization of non-preferred carbon sources to thwart induction of their catabolic operons, and b) induction prevention, i.e., control of the activity of operon-specific transcription factors [32].

a) In the case of induction exclusion, approximately 10 % of the down or up-regulated genes from each transcriptome were implicated in transport functions (Fig. 4a). Glucose repression was detected over the maltose transporter *malG* and *malF* genes (*SCO2229*-*SCO2230*) and the putative operon *SCO3482*-*SCO3484* (coding for a sugar permease, an integral membrane transport protein and a sugar-binding protein) were repressed (Fig. 4b). It is noteworthy mentioning that only a limited number of sugar transporter genes were detected in this study, probably due to the lack of specific inducers in the system. Interestingly, the xylose transporter, (*SCO6009*-*SCO6011*) showed 10-fold stimulation, just after the GlcP transporter (Fig. 4a and b). This oddly up-regulation of the xylose transporter was also accompanied by changes in its transcriptional regulator Rok7B7 (log_2_ = 2.14) (Additional file 1: Table S1). Recently, it has been shown that the protein Rok7B7 is also involved in *S. coelicolor* CCR [33], but its function in this phenomenon remains unclear.

The genes involved in glutamate incorporation (*SCO5774*-*77*), encoding GluD, GluC, GluB and GluA, respectively), were strongly stimulated (about 16-fold up-regulation) by glucose (A and B). It was not surprising to find that glucose did not exert a repressive effect over the glutamate transporter since this amino acid is a preferred carbon source, even over glucose [11]. Besides, in the *Sco*M145/*ScoZm* comparison, this transporter was also up-regulated in *ScoZm* when compared to *Sco*M145. However, it is unclear why and how glucose stimulates the glutamate permease and how is the ScGlk effect mediated. Likely, this is the result of transcriptional changes in the arginine repressor, which will be discussed later.

For induction prevention, it is well known that glucose represses glycerol kinase (GlpK) production [10] by negatively influencing transcription of the glycerol-inducible *glPFKDX* operon [34]. This operon comprises genes for a putative glycerol transporter, a glycerol kinase, a glycerol-3-phosphate dehydrogenase, and a gene of unknown function. In the Glc/Agar comparison, the glycerol kinase encoded by *SCO1660* (log_2_ = −0.98) was repressed while the glycerol operon repressor protein (GylR) was up-regulated by glucose (log_2_ = 1.1). In addition, a transcriptional repression of *glpK* was observed, confirming the findings of Hindle and Smith [34] and supporting that the GylR effect is due to an operon specific mechanism rather than a pleiotropic one. Interestingly, no transcriptional changes were detected in this operon when comparing the *Sco*M145 with *ScoZm*, suggesting a ScGlk-independent repression of *glpK* or induction of *gylR*.

Since many operons, specific CCR mechanisms require the presence of inductor we choose a classical and widely model to evaluate the effect of glucose and ScGlk over the utilization of an alternative carbon source looking for transcriptional changes in agar metabolism.

### Utilization of an alternative carbon source

#### Agar metabolism

Agar utilization is one of the most widely used tests to assess for CCR. Agar is constituted by a family of complex polysaccharides found in the cell wall of some red algae cells. Primarily, this polymer consists of agarobiose (G-LA) and agaropectin. Agarobiose is formed of linear chains of alternating residues of 3-*O*-linked β-D-galactopyranose and 4-*O*-linked 3, 6-anhydro-α-L-galactose (LA) while agaropectin seems to be formed by sulfate esters, pyruvate acetal and methyl esters, but its exact structure remains obscure [35, 36].

**Fig. 5:**
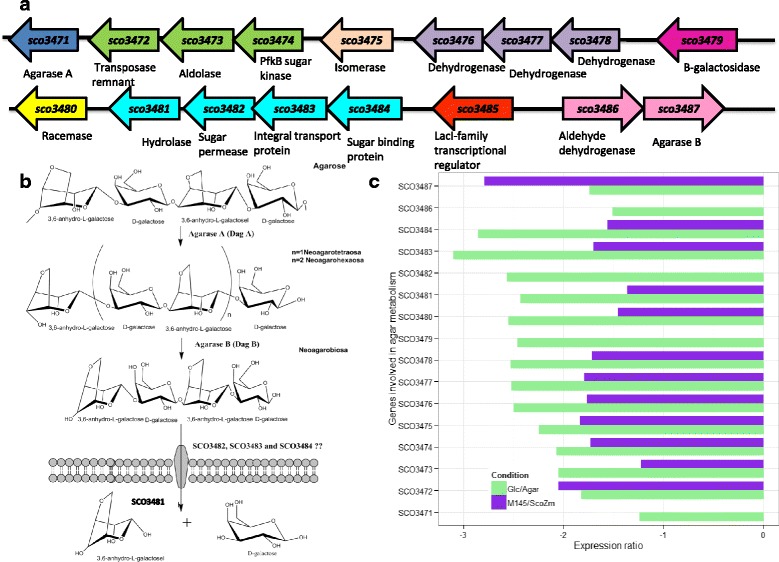
**a** Genomic context of a cluster possible involved in agar degradation. **b** The Agar metabolism. **c** Average expression changes observed among experimental conditions. Glc/Agar comparison reflects the glucose effect and M145/*ScoZm* comparison shows the Glk dependence (*green bars*). The comparison between these conditions is indicated as ratios of Glc/Agar (carbon dependence) and *Sco*M145/*Sco*Zm (Glk dependence in *purple bars*). Results are given as the log2 ratio, thus a positive number indicates up-regulation and a negative number down-regulation, respectively

The genomic context of a cluster involved in agar degradation is presented in Fig. 5a [37]. Complete agar hydrolysis produces monomeric sugars such as D-galactose (Gal), LA, and L-galactose-6-sulfate (Fig. 5b) [38]. Although used as a model to measure CCR, the *S. coelicolor* agarolytic pathway has not been widely studied. The first enzyme described in this pathway was an endo-type agarase that degrades agar into neoagarotetraose and neoagarohexaose, β-agarase A, DagA (coded by *SCO3471*). Its transcription, controlled by four different promoters, is induced by agar but repressed by glucose [16, 39]. A second agarase (Dag B), was recently identified. This enzyme (codified by *SCO3487*) is an exo and endo-type β-agarase that degrades agarose, neoagarotetraose and neoagarohexaose into neoagarobiose [35]. In the proposed agarolytic pathway DagA and DagB can hydrolyze agarose into neoagarobiose in a cooperative way [35]. Likely, neoagarobiose is transported into the cell by an unknown transporter and once inside the cell, a cytosol glycoside hydrolase (encoded by *SCO3481*) hydrolyze neoagarobiose into Gal and LA [36, 38]. The reconstructed agarolytic pathway proposed by Chi et al. [38] is summarized in Fig. 5b.

As expected, the genes encoding for agarases (*SCO3471* and *SCO3487*), were repressed by glucose in Glc/Agar comparison. This effect was observed in a large genomic region (*SCO3471*-*SCO3487*), except for the *SCO3485* that encodes for a LacI-transcriptional regulator (Fig. 5c). This region (Fig. 5a) contains nine possible transcription units (18 genes in 9 operons), although the function of only three of them has been described (DagA, DagB and SCO3481). Previous attempts to find the transcriptional regulator of DagA have failed [39]. However, in our tested conditions, no changes were detected in the only gene encoding a putative transcriptional regulator (*SCO3485*) present in this agar degradation cluster. The functions of this putative regulator are unknown, but apparently it is not transcriptionally regulated by glucose.

In the *Sco*M145/*ScoZm* comparison, genes from the agar degradation cluster showed higher repression in the *Sco*M145 strain compared to *ScoZm* (Fig. 5c), particularly that of *SCO3487* which codes for DagB (Fig. 5a) implying, as previously reported, a repressive effect dependent on ScGlk *per se*. It is hard to establish the mechanism by which the ScGlk protein itself could exert a regulatory function. As mentioned before, at the central carbon metabolism (glycolysis), and hence the metabolites produced, operates in a similar way when endogenous ScGlk is present and when is substituted by a non-related Glk. So the repression, at least that of agar metabolism, most be not dependent on the glycolytic flux, as previously proposed [16], but dependent on the expression of DNA binding proteins and its interactions with ScGlk. It is becoming evident that besides the specific operon regulators, the presence of other pleiotropic or general regulators such as the recently described Rok7B7 [33] may be important in understanding the CCR in *Streptomyces* and also in establishing the ScGlk effect. In the next section, the transcriptional changes in genes coding for putative transcriptional regulators responsive to glucose and ScGlk are described.

**Fig. 6:**
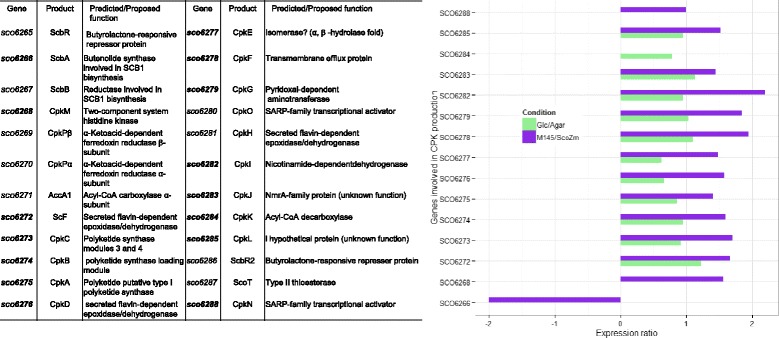
Genes and its predicted products involved in CPK synthesis (*on the left*) (adapted from Gómez-Escribano et al. [47]). *On the right*, transcriptomic expression of the CPK genes. Glc/Agar comparison (*green bars*) reflects the glucose effect on CPK production and the M145/*ScoZm* comparison (*purple bars*) shows its Glk dependence. Comparisons between these conditions are indicated as ratios of Glc/Agar (carbon dependence) and *Sco*M145/*ScoZm* (Glk dependence). Results are given as log2 ratio, thus positive numbers indicate up-regulation and negative numbers down-regulation, respectively

Interestingly, in this study, a possible transporter associated with neoagarobiose incorporation (encoded by *SCO3482*-*84*), was found (Fig. 6c). Since this chromosomal region is implicated in agar metabolism, genes *SCO3482*-*84* showed the same expression pattern observed for agarases and genes in this region seemed to respond to a common regulation. The gene products of SCO3482 and SCO3483 have 7 transmembrane domains (TMs) and are therefore predicted to be membrane proteins. On the other hand, SCO3484 does not have any TM and, therefore, it is predicted to be the sugar binding protein (http://www.membranetransport.org). Likely SCO3484 is the transporter associated with neoagarabiose internalization. Regarding to agar utilization, this is the first report describing the genes and transcriptomic changes in a cluster involved in agar metabolism.

### CCR and transcription regulators

Prokaryotic cells can adapt and respond to environmental conditions by modifying their genome expression pattern; therefore, as previously proposed, describing the transcriptional dynamics mediated by regulatory proteins could be an important clue to unraveling CCR mechanisms. In model organisms, transcriptional regulators operate both global and operon-specific mechanisms. In our study, around 15 % of all differentially expressed genes in Glc/Agar and *Sco*M145/*ScoZm* comparisons, comprised GO regulation of metabolic processes and stimuli responses (Fig. 3). Transcriptional factors stimulated by glucose were found among the MerR, LacI, TetR, GntR, MarR and AsnC families, as well as sigma factors (Table 1). On the other hand, transcriptional factors repressed by glucose included members of the GntR, MarR, and DeoR families, but also sigma and anti-sigma factors (Table 1). Down-regulated transcriptional regulators with known functions included: the RNA polymerase sigma factor SigU (σU), the anti-sigma factor RsuA, the sporulation transcription factor WhiH, as well as the RNA polymerase sigma factor BldN. Up-regulated transcriptional regulators were: the regulatory protein (GlnR), the glycerol operon regulatory protein (GylR), and the regulatory protein Rok7B7 previously discussed.

**Table 1:** Transcriptional regulators differentially expressed in the Glc/Agar comparison

Transcriptional factors down-regulated in Glc/Agar		Transcriptional factors up-regulated in Glc/Agar	
Gene	Family or putative function	Gene	Family or putative function
*SCO1541*	Regulator	*SCO0140*	MerR family transcriptional regulator
*SCO1699*	Transcriptional regulator	*SCO2489*	TetR family transcriptional regulator
*SCO2209*	Transcriptional regulator	*SCO2935*	Transcriptional regulator
*SCO3134*	Two-component system response regulator	*SCO3361*	Transcriptional regulator AsnC
*SCO3933*	Regulatory protein	*SCO3423*	Regulator
*SCO3986*	GntR family transcriptional regulator	*SCO3810*	GntR family transcriptional regulator
*SCO4122*	MarR family transcriptional regulator	*SCO3943*	Transcriptional regulator
*SCO4412*	Regulatory protein	*SCO4020*	Two component system response regulator
*SCO4677*	Regulatory protein	*SCO4158*	LacI-family regulatory protein
*SCO4920*	DeoR family transcriptional regulator	*SCO4640*	TetR family transcriptional regulator
*SCO5785*	Two-component system response regulator	*SCO4850*	TetR family transcriptional regulator
*SCO5811*	Transcriptional regulator	*SCO5413*	MarR-transcriptional regulator
*SCO5819*	Sporulation transcription factor	*SCO5552*	Regulator
*SCO6162*	Two-component system response regulator	*SCO5982*	Regulator
*SCO6992*	Regulatory protein	*SCO7424*	MarR family transcriptional regulator
*SCO7727*	MarR family regulatory protein	*SCO1658*	Glycerol operon regulatory protein GylR
*SCO2954*	RNA polymerase sigma factor SigU (σ^U^)	*SCO2950*	Nucleoid associated protein hupA
*SCO2953*	Anti-sigma factor RsuA	*SCO4159*	Regulatory protein GlnR
*SCO5819*	Sporulation transcription factor WhiH	*SCO4762*	Chaperone GroLE1
*SCO3323*	RNA polymerase sigma factor BldN	*SCO6008*	Regulatory protein Rok7B7

The transcriptional factors activated by glucose can be divided into three groups: a) pleiotropic regulators, b) regulators of specific operons and c) DNA-associated proteins.

Regarding pleiotropic regulators, the GlnR regulator functions as the main nitrogen regulator [40], but it is also involved in different metabolic processes such as carbon metabolism, synthesis of catabolic enzymes, and secondary metabolism [41]. The recently described regulator Rok7B7 (*SCO6008*) was activated by glucose in the *Sco*M145 background, following the same activation pattern as the XylFGH operon. Likely, Rok7B7 may act as a positive regulator. Thus, in addition to regulating the xylose operon, Rok7B7 activates morphological development and actinorhodine production [33]. On the contrary, this regulator blocked the calcium dependent antibiotic and prodigiosin formation. Rok7B7 is also known to be involved in the CCR process [33].

Regarding regulators of specific operons, the only specific regulator gene observed with differential expression was the *gylR* gene (encoding a glycerol operon repressor). This regulator was stimulated by glucose and in agreement with this effect, the glycerol kinase gene (*SCO1660*) was repressed.

On DNA associated proteins, HupA expression was stimulated by glucose. HupA is a protein found in vegetative mycelium growing in liquid culture [42] and involved in gene expression, DNA protection (recombination or repair) and nucleoid structuring. Additionally, transcription of the chaperonine GroEL1 was also stimulated by glucose and was heat shock activated [43]. An interesting regulator with an unknown function is SCO6162, a probable member of the luxR family (two-component system response regulator), which showed more than 20-fold down-regulation as well as its cognate kinase (differentially expressed by log_2_ = −2.09). Markedly, the divergent gene *SCO6164* (encoding a hypothetical protein), exhibited the highest glucose repression (log_2_ = –5.26).

In the *Sco*M145/*ScoZm* comparison, the four genes encoding regulatory proteins that showed stimulation belonged to the GntR (SCO3264, SCO3986), LuxR families (SCO4261) and a transcriptional activator (SCO6288) (Additional file 1: Table S2). The two regulators belonging to the GntR family, the SCO3264, and SCO3986, were highly overexpressed (log2 = 2.5 and 3.77, respectively). Remarkably, none of these up-regulated transcriptional factors have been previously described. The *in silico* prediction of the putative targets of *SCO3264* has revealed a large set of sensitive genes involved in primary and secondary metabolism. Actually, in a *S. coelicolor* ∆*SCO3264* mutant constructed in our lab, a premature and high-level production of secondary metabolites was observed (Manzo et al., unpublished results).

On the other side, in the group of transcriptional down-expressed regulators, the arginine metabolism and its regulator ArgR (*SCO1576*), deserve special attention (Table 2) [44]. In a recent transcriptomic and proteomic comparison between *Sco*M145 and its ∆*argR* mutant, the versatile functions of this regulator were demonstrated [44]. Beyond its regulatory role in the arginine and pyrimidine biosynthesis, ArgR was also able to regulate some different transcriptional factors from different families such as DeoR, AraC and GntR [44]. Therefore, stimulation or repression of ArgR affected secondary metabolite production, morphology and synthesis of proteases and peptidases. The versatile functions of this regulator and its higher expression in the absence of ScGlk suggest a complex regulatory network. To make this panorama more complex, when *Sco*M145/*ScoZm* were compared, none of the differentially expressed genes of the putative transcriptional regulators seemed to be the transcriptional regulators found by Pérez-Redondo [44].

**Table 2:** Differentially expressed genes found in the *Sco*M145/*ScoZm* comparison

Gene name	Product	Function
*SCO0016*		Hypothetical protein
*SCO0138*		Short chain dehydrogenase
*SCO0555*		Membrane-bound oxidoreductase
*SCO0556*		Hypothetical protein
*SCO1570*	argH, SCL24.06c	Argininosuccinate lyase [EC:4.3.2.1]
*SCO1576*	argR, SCL24.12c	Arginine repressor
*SCO1577*	argD, SCL24.13c	Acetonitrile aminotransferase [EC:2.6.1.11]
*SCO1578*	argB, SCL24.14c	Acetylglutamate kinase [EC:2.7.2.8]
*SCO1579*	argJ, SCL24.15c	Putative glutamate N-acetyltransferase [EC:2.3.1.1 2.3.1.35]
*SCO1580*	argC	N-Acetyl-gamma-glutamyl-phosphate reductase [EC:1.2.1.38]
*SCO1815*	fabG, SCI28.09c	Probable 3-oxacyl-(acyl-carrier-protein) reductase [EC:1.1.1.100]
*SCO2126*	glk, SC6E10.20c	Glucokinase [EC:2.7.1.2]
*SCO2296*		Integral membrane protein
*SCO2513*		Hypothetical protein
*SCO2930*		Putative permease membrane component
*SCO2986*		Hypothetical protein
*SCO3138*	galT, SCE66.17c	Galactose-1-phosphate uridylyltransferase [EC:2.7.7.10]
*SCO3411*		Possible membrane protein
*SCO3413*		Transcriptional regulator
*SCO3985*		Hypothetical protein
*SCO4173*		Hypothetical protein
*SCO4174*		Integral membrane protein
*SCO4175*		Hypothetical protein
*SCO4317*		Hypothetical protein
*SCO4903*		Hypothetical protein
*SCO4950*		Nitrate reductase gamma chain NarI3
*SCO5025*		Transcriptional regulator
*SCO5026*		Hypothetical protein
*SCO5367*		ATP synthase A chain [EC:3.6.3.14]
*SCO5536*		Hypothetical protein
*SCO5839*		Hypothetical protein
*SCO5976*	arcB, StBAC16H6.11	Ornithine carbamoyltransferase [EC:2.1.3.3]
*SCO6266*		ScbA protein
*SCO6268*		Histidine kinase
*SCO6288*		Regulatory protein
*SCO7036*	argG, SC4G1.02	Argininosuccinate synthase [EC:6.3.4.5]
*SCO7262*		Hypothetical protein
*SCO7530*		Regulatory protein
*SCO7586*		Oxidoreductase
*SCO7587*		Integral membrane protein
*SCO7698*		MerR-family transcriptional regulator

### Role of ScGlk in carbon catabolite repression

As previously mentioned, the transcriptional response to glucose (Glc/Agar comparison) included 651 differentially expressed genes while in the ScGlk response (*Sco*M145/*ScoZm* comparison) 134 differentially expressed genes were identified. From the total of differentially expressed genes in *Sco*M145/*ScoZm* comparison, 91 were included in the Glc/Agar comparison (this information can be seen in Additional file 1: Figure S1). This result conferred only a limited regulatory function to this enzyme in CCR, since specifically affected only 43 out of a total of 779 genes. This contrasted with previous results, which proposed a significant role of Glk in CCR [10, 14, 19, 23]. As seen in Additional file 1: Table S3 several of this shared genes were involved in transport functions (*SCO2306*, *SCO2463*-*2464*, *SCO3717*, *SCO3719*, *SCO5774*-*5777*) and agar metabolism (*SCO3471*-*SCO3485*, *SCO3486*-*3487*). Concerning transport functions, these included genes encoding ABC transporters (*SCO2306*, *SCO2463*-*2464*), cation transporters like *SCO3717*-*SCO3719*, amino acid transporters as the glutamate permease (*SCO5774*-*SCO5777*) and also the transporter putatively associated with neoagarabiose internalization (SCO3483-SCO3484). The genes encoding the ABC transporter (*SCO2463*-*SCO2464*) were stimulated by glucose in the wild-type strain, but repressed in the *Sco*M145/*ScoZm* comparison. As mentioned previously, the genes involved in agar metabolism were repressed by glucose and also were affected by ScGlk.

Aside from primary metabolic pathways and transport systems, significant transcriptional changes in the yellow cryptic polyketide (yCPK) cluster was also observed in both transcriptomes, and, therefore, deserved special mention. The repressive effects exerted by glucose on differentiation and secondary metabolite production are well known ([27], Romero et al. unpublished data). However, in our experimental conditions, relative to agar, the *cpk c*luster was up-regulated by glucose. Previously, Pawlick et al. [45] reported that the presumptive product of the CpK polyketide synthase is a yellow compound secreted into the medium. Its production depended on the medium composition, inoculum density and required the absence of glucose. In parallel, Gottelt et al. [46] reported in *S. coelicolor* that CPK production is enhanced by glutamate supplementation. Recently, in a *S. coelicolor* engineered strain, the product of this cluster has been named coelimycin P1 and suggested to be a glutamate adduct [47].

In the Glc/Agar comparison, glucose exerted a stimulatory effect on the genes *SCO6272*-*SCO6279* (mostly polyketide synthases) and *SCO6282*-*SCO6285* (encoding a dehydrogenase, a decarboxylase and two hypothetical proteins) (Fig. 6). Additionally, in the *Sco*M145/*ScoZm* comparison, *SCO6268* (encoding a putative histidine kinase) and *SCO6288* (encoding a SARP-transcriptional activator), also showed increased expression in the *Sco*M145 strain, relative to the *ScoZm* mutant and this effect was not observed in the Glc/Agar comparison. Although Pawlick et al. [45] previously reported that glucose exerts a negative effect on the synthesis of yCPK, in our experimental conditions this carbohydrate exerted an up-regulation on the *cpk* genes. Interestingly, in the absence of ScGlk a reduced expression of *cpk* genes was observed in *ScoZm*, relative to *Sco*M145. In the model proposed by Gubbens et al. [27], CpK synthesis is activated by glucose + mannitol and repressed by glucose + fructose. It would be interesting to test the *ScoZm* in glucose + fructose or any other carbon source combinations to evaluate their effect on the *cpk* cluster expression. As Gubbens et al. [27] anticipated for the cpk secondary metabolite production, the influence of Glk likely involves a complex regulatory mechanism, also dependent on nutritional signals.

From the proposed genes involved in the CPK synthesis, the only one that showed a down-regulation in *ScoM145*, relative to *ScoZm*, was *SCO6266*, which encodes a butenolide synthase (Fig. 6). Additionally, in the *Sco*M145/*ScoZm* comparison, *SCO6268* (encoding a putative histidine kinase) and *SCO6288* (encoding a SARP-transcriptional activator), also showed increased expression in the *Sco*M45 strain, relative to the *ScoZm* mutant and this effect was not observed in the Glc/Agar comparison. Depending on the used carbon source, proteomic studies have also reported the differential expression of proteins of this cluster [27].

Regarding the genes that were exclusively found in the *Sco*M145/*ScoZm* comparison (Table 2), as previously discussed, the gene encoding the versatile regulator ArgR (*SCO1576*) was down-regulated in the *Sco*M145 strain (Table 1), when compared to *ScoZm*. It is interesting to note that the absence of ScGlk (SCO2126) somehow generates a higher transcription of the gene encoding the regulator ArgR in the *ScoZm* strain, relative to *Sco*M145. In agreement with the up-regulation of ArgR genes, the arginine biosynthetic pathway showed down-regulation on the mutant or, in other words, the arginine biosynthetic genes were up-regulated in the *ScoZm* when compared *Sco*M145 (Table 2).

### RT-PCR validation of selected genes up- or down-regulated by glucose or ScGlk

Microarray data were validated by RT-qPCR utilizing glucose-stimulated (*rok7b7* and *glcP*) and repressed (*dagA*, *SCO6162* and *SCO6164*) genes in Glc/Agar comparison. To look for suitable reference genes, the sigma factor gene *hrdB* [42], the beta chain from DNA polymerase III (*dnaN*), the DNA gyrase subunit A gene (*gyrA*), the recombinase A gene (*recA*) and the DNA-directed RNA polymerase alpha chain (*rpoA*) [48] were utilized. To select the most stable reference genes, the BestKeeper program was utilized [49]. This program calculates a BestKeeper index, which is the geometric mean of all housekeeping raw CT values. Pearson correlations between individual genes and the BestKeeper index were calculated and reported as the BestKeeper correlation coefficient. Genes with the highest correlation coefficient were considered the most stably expressed. In our conditions, *recA*, *rpoA*, and *gyr*A were selected as reference genes due to their high BestKeeper index and low standard deviation (Additional file 1: Figure S3). The geometrically averaged reference genes were used to normalize the following target genes: *dagA*, *glcP*, *rok7b7*, *SCO6162* and *SCO6164*. The relative transcript profile (Additional file 1: Figure S3) of the target genes corresponded well to the microarray analysis, confirming our results on glucose regulation. Also, *glcP* and *rok7B7* were largely induced by glucose, while expression of *dagA*, *SCO6162* and *SCO6164*, were largely repressed. Their neighboring genes (*SCO6160*-*SCO6167 and SCO6173*) also were down-regulated by glucose. The SCO6162 and its cognate kinase SCO6163 constitute a two-component system. The response regulator (SCO6162) belongs to the LuxR family of transcriptional regulators and is conserved in many *Streptomyces* species (http://blast.ncbi.nlm.nih.gov/). Its domain architecture is present in many activators. The two component systems are important to sense and respond to the environment. A great unexplored area in *Streptomyces* CCR is the potential role of two-component systems. In other soil living or environmental strains such *Pseudomonas*, *Sinorhizobium* and *Bacillus*, two-component systems are important in CCR control [50–52].

## Conclusions

Glucose can elicit significant metabolic and transcriptional changes in *S. coelicolor*. Whereas glucose influx and its flow through glycolysis and pentose phosphate pathway were stimulated, gluconeogenesis was inhibited. Glucose also triggered carbon catabolic repression by repressing a number of transporter systems and repressing transcription of enzymes needed for the utilization of secondary carbon sources. Additionally, 40 DNA binding proteins, including 31 transcriptional regulators and four two component systems were regulated by glucose and probably were involved in the signals and the effects elicited by this carbon source.

The ScGlk enzyme *per se* was required for the glucose repression, at least in the agar model proposed by Angel et al. [16]. In this regard, our results confirm and update the agar model of CCR in *Streptomyces* and its dependence on ScGlk *per se*. Nevertheless, the expected regulatory function was not as global as thought before, but likely may be accompanied or coordinated by other transcriptional regulators. The remaining transcription factors, whose expression was affected by glucose or ScGlk with unknown functions, are an interesting area to describe in the *S. coelicolor* CCR. Currently, we are constructing regulatory networks (Romero et al. unpublished results) to find out the possible regulatory relations between genes regulated by glucose and by ScGlk. Understanding the functions of the glucose-targeted transcriptional regulators and their transcriptional network may lead to unraveling one of the most fundamental regulatory mechanisms in the *Streptomyces* metabolism.

## Methods

### Bacterial strains and plasmids

Bacterial strains and plasmids used in this work are listed in Table 3. All *Streptomyces* strains were stored as spore suspensions in 20 % (v/v) glycerol at −20 °C. *E. coli* DH5-α [53] was used for routine cloning procedures, while *E. coli* ET12567 [54] was used to obtain unmethylated DNA for intergeneric conjugations. For *Streptomyces* strains carrying the pIJ402 and pUNAMZm, thiostrepton was used at a final concentration of 10 μg/ml.

**Table 3:** Strains and plasmids used in this work

Strains/plasmids	Characteristics	Reference
*S. coelicolor* M145	SCP1^−^ SCP2^−^	[61]
*S. coelicolor* ∆*glk*	SCP1^−^ SCP2^−^ *glk*::*aacc4*	This work
*S. coelicolo*r Zm	*S. coelicolor* ∆*glk*, harboring the plasmid pUNAMZm	This work
*S. coelicolo*r 702	*S. coelicolor* ∆*glk*, harboring the plasmid pIJ702	This work
*E. coli* ET12567	F-*dam*-13::Tn9 *dcm*-6 *hsd*M *hsd*R *zjj*-202::Tn10 *rec*F143 *gal*K2 *gal*T22 *ara*-14 *lac*Y1 *xyl*-5 *leu*B6 *thi*-1 *ton*A31 *rps*L136 *his*G4 *tsx*-78 *mtl*-1 *gln*V44	[62]
*E. coli* DH5α		Invitrogen
Plasmids		
pIJ702	Derived from pIJ101 with an estimated copy number of approximately 50	[16]; John Innes Centre
pIJ2442	Derivative of pUC19 containing the1.7 pb PstI-XbaI *Z. mobilis glk* fragment	[16]; John Innes Centre
pUNAM Zm	Plasmid pIJ702 harboring the 1.7 pb PstI-XbaI *Z. mobilis glk* fragment from pIJ2442	This work

### Media and growth conditions

*E. coli* strains were grown in Luria-Bertani medium [53]. All media and routine *Streptomyces* techniques are described in the *Streptomyces* manual [54].

For carbon utilization and microarrays, glycerol spore suspensions (10^8^ ml^−1^) were thawed, sedimented (6000 rpm for 5 min), washed once with distilled water and resuspended in 20 ml 2× YT medium and incubated at 30 °C for 8–10 h at 30 °C in 250-ml flasks at 250 rpm. Pre-germinated spores were harvested by centrifugation and resuspended in NMMP minimal medium (10 ml) to give an initial OD of 0.05 at 490 nm. They were resuspended in 50 ml of NMMP supplemented with either 1 % (w/v) agar or 0.5 % glucose and 0.5 % agar, as carbon sources. The agar was partially hydrolyzed as previous described [39]. Glk activity was measured in cultures grown in TSB medium. Samples were taken once the exponential growth phase was reached (12 h for *S. coelicolor* and 16 h for *ScoZm*).

### Glucose kinase activity

Qualitative in vivo visualization of Glk activity was performed in BPG Agar (BPG) medium, which contained (g L^−1^) peptone 5.0, meat extract 3.0, glucose 10.0, bromocresol purple 0.025 and agar 20.0, at pH 7.0. Activity was observed as a change in the colony color from purple to yellow, due to the organic acids produced from glucose catabolism. Also, Glk activity was spectrophotometrically measured by monitoring NADP reduction (ε = 6.22 cm^−1^ mM^−1^) in a glucose-6-phosphate dehydrogenase coupled reaction, as reported previously [55].

### Construction of the *S. coelicolor* ∆*glk* mutant

The *glk* gene (*SCO2126*) of *S. coelicolor* M145 (*Sco*M145) was replaced by the apramycin resistance cassette *aacC4* using the PCR targeting procedure of Gust et al. [56]. For this purpose, the cosmid StC6E10, carrying the *glk* gene was utilized. After recombination, the resulting ∆*glk* putative mutants were verified by PCR and sequenced to confirm their replacement. Subsequently, their Glk activity was determined [55].

For heterologous complementation, the plasmid pIJ2442 (John Innes Centre, UK) was digested with *Pst*I and *Sac*I to generate a 1.7 Kb fragment containing the *Z. mobilis glk* gene. The *Z. mobilis* fragment was subcloned into the plasmid pIJ702 producing the pUNAMZm plasmid. By using the PEG-assisted protoplast transformation technique [54], plasmids pIJ702 and pUNAMZm were used to transform the ∆*glk* mutant, generating the *Sco702* (with an empty plasmid) and *ScoZm* strains (with the inserted *Z. mobilis glk*), respectively.

### RNA isolation and DNA microarray analysis

*S. coelicolor* strains used for RNA extraction were grown as previously mentioned. Cultures were grown at 250 rpm and 30 °C, until reaching the exponential growth phase was reached (12 h for *S. coelicolor* and 16 h for *ScoZm*). At these times, cultures were collected by filtration on a Whatman filter paper number 4. Filtered mycelium was stabilized with the RNAprotect reagent from Qiagen, according to manufacturer instructions. RNA integrity was checked on an Agilent 2100 Bioanalyzer (Agilent Technologies) and an agarose gel. Four independent biological replicates of each condition were prepared. Amplifying in the absence of a reverse transcriptase checked the lack of DNA. cDNA preparation, labeling, and hybridization were performed as previously described by Lewis et al. [57], and in http://www.surrey.ac.uk/fhms/microarrays/Downloads/Protocols/Strep_hyb_protocol_1005.pdf). Total RNA was labeled with Cy3-dCTP or Cy5-dCTP. To remove any dye bias, a “dye-balance” (dye-swap) was performed. For microarray analysis, the 104 K x-60mer whole-genome *Streptomyces* array (Agilent Technologies) was utilized [57], and it comprises almost 104,000 unique 60-mers with an average spacing of 30 nucleotides [57]. To detect differentially expressed genes (up or down regulated), the filtered data sets were analyzed using the rank product analysis [21].

The microarray design and data can be found in the ArrayExpress database (www.ebi.ac.uk/arrayexpress) [E-MTAB-3602, E-MTAB-3603 for *Sco*M145/*ScoZm* and Glc/Agar comparisons, respectively].

### Annotation, pathways and GO analyzes

A ranked gene list was produced from microarray data using a pfp value of <0.15. The resultant list was submitted to Protein Center (Thermo®). This software generates a list of ontology terms (GO). The same ranked list was used for pathway discovery using the Kyoto Encyclopedia of Genes and Genomes (KEGG) (http://www.genome.jp/kegg/) and the Database Collection (BioCyc) (http://biocyc.org) websites. Also, information from Surrey Analysis Tools (http://strep-microarray.sbs.surrey.ac.uk/) was useful in Additional file 1: Table S1 and Additional file 1: Table S2 construction.

### RT-qPCR

To verify the output from the microarray analysis, a quantitative RT-PCR analysis was performed. The RNA samples were treated as the microarray samples. cDNA was synthesized using SuperScript® III Reverse Transcriptase (Invitrogen), following manufacturer’s instructions. Each reaction contained 1 μg RNA, random hexamers and the outcome cDNAs were utilized as templates for RT-qPCR assays.

RT-qPCR was performed in a 10 μL volume reaction containing 1 μL diluted cDNA with a final primer concentration of 0.5 μM, and 1× Master Mix. A three-step amplification protocol was performed in the StepOne Real Time PCR System (Life Technologies, USA), using the MaximaR SYBR Green/ROX qPCR Master Mix (2×) kit (Life Technologies, USA). An initial one cycle denaturation step was performed at 95 °C for 10 min. Subsequently, 40 cycles of 30 s at 95 °C for target amplification, 60 cycles of 30 s at 60 °C for annealing and then an extension for 30 s at 72 °C. After 40 amplification cycles, the quality of PCR products was evaluated using melt curve analysis. Reactions were performed in triplicate, and Ct values were averaged. Replicates and negative controls were included to detect contamination. Standard curves were used to evaluate primer efficiency. All RT-qPCR experiments fulfilled the MIQE guidelines (Minimum Information for Publication of Quantitative Real-Time PCR Experiments) [58, 59]. The primers were designed using the PrimerQuest tool from Integrated DNA Technologies. Nucleotide sequences of these primers are shown in Additional file 1: Table S4 of the supplementary material. The length of all utilized oligonucleotides (forward and reverse), was between 18 and 21 nucleotides, with GC content between 45 to 60 % and Tm values between 58 to 60 °C. The final size of PCR products was from 90 to 120 bp.

The quantification technique used to analyze the data was that proposed by Pfaffl [60]. The BestKeeper software [49] was used to select the most stable genes utilized as references. To increase the procedure robustness, data were normalized using the reference genes *rpoA*, *gyrA* and *recA*.

### Ethical approval and consent to participate

Not applicable.

### Consent for publication

Not applicable.

### Availability of data and materials

The microarray design and data are available in the ArrayExpress database (www.ebi.ac.uk/arrayexpress) [E-MTAB-3602, E-MTAB-3603 for *Sco*M145/*ScoZm* and Glc/Agar comparisons, respectively].

## Additional file

Additional file 1: Figure S1. This file contains the qualitative visualization of Glk activity on BPG Agar medium. **Figure S2.** This file contains a diagram summarizing the microarrays output and their intersection. **Figure S3.** This file contains the RT-qPCR validation of selected differentially expressed genes. **Table S1.** Complete dataset of differentially expressed genes at statistically significant levels (pfp values ≤0.15) of *S. coelicolor* cells grown in 0.5 % glucose + 0.5 % agar, relative to cells grown in 1 % agar. **Table S2.** Data set of differentially expressed genes at statistically significant levels (pfp values ≤0.15) in the *Sco*M145 wild-type strain, relative to the *ScoZm* mutant. **Table S3.** Including differentially expressed genes shared between Glc/Agar and *Sco*M145/*ScoZm* comparisons. **Table S4.** Lists oligonucleotides used in this study. (PDF 1450 kb)

## Abbreviations

Aco: acotinase
AKGdh: α-ketoglutarate dehydrogenase
ArgR: arginine repressor
CcpA: catabolite control protein
CCR: carbon catabolite repression
Crp: cAMP-receptor protein
Cs: citrate synthase
DagA: agarase A
DagB: agarase B
EMP: embden-meyerhof-parnas pathway
Eno: enolase
Fba: fructose-1,6 biphosphate aldolase
Fum: fumarase
Gap: glyceraldehyde-3 phosphate dehydrogenase
Ghd: 6-phosphogluconate dehydrogenase
G-LA: agarobiose
Glc: glucose
Gnk: gluconokinase
GntK: gluconate kinase
GntP: gluconate permease
GTA: global transcriptomic analysis
Idh: isocitrate dehydrogenase
LA: 6-anhydro-α-l-galactose
malG and malF: maltose transporter subunits G and F
Mdh: malate dehydrogenase
MFS: major facilitator system
Pdh: pyruvate dehydrogenase complex
PEPc: phosphoenolpyruvate carboxylase
PEPck: phosphoenolpyruvate carboxykinase
Pfk: 6-phosphofructokinase
Pgi: phosphoglucose isomerase
Pgk: phosphoglycerate kinase
Pgl: 6-phosphogluconate-δ-lactone
Pgm: phosphoglycerate mutase
PPP: pentose phosphate pathway
PTS: phosphoenolpyruvate-dependent phosphotransferase system
Pyk: pyruvate kinase
ROK: signature (repressor, ORF, kinase)
RPA: rank products analysis method
Rpe: ribose-5-phosphate epimerase
Rpi: ribose-5-phosphate isomerase
ScGlk: glucokinase from *streptomyces coelicolor*
Sdh: succinate dehydrogenase
Suc: succinyl CoA synthetase
Tal: transaldolase
TCA: tricarboxylic acid cycle
Tkt: transketolase
Tpi: triosephosphate isomerase
*Zm*Glkv: glucokinase from *zimomonas mobilis*
Zwf: glucose-6-phosphate dehydrogenase
