# Supplementary material for: Transcriptomic analysis of a classical model of carbon catabolite regulation in Streptomyces coelicolor
Source: BMC Microbiol. 2016 Apr 27;16:77. doi: 10.1186/s12866-016-0690-y (PMC4848846; doi:10.1186/s12866-016-0690-y)
Supplement: Additional file 1: Figure S1. — This file contains the qualitative visualization of Glk activity on BPG Agar medium. Figure S2. This file contains a diagram summarizing the microarrays output and their intersection. Figure S3. This file contains the RT-qPCR validation of selected differentially expressed genes. Table S1. Complete dataset of differentially expressed genes at statistically significant levels (pfp values ≤0.15) of S. coelicolor cells grown in 0.5 % glucose + 0.5 % agar, relative to cells grown in 1 % agar. Table S2. Data set of differentially expressed genes at statistically significant levels (pfp values ≤0.15) in the ScoM145 wild-type strain, relative to the ScoZm mutant. Table S3. Including differentially expressed genes shared between Glc/Agar and ScoM145/ScoZm comparisons. Table S4. Lists oligonucleotides used in this study. (PDF 1450 kb) [file 12866_2016_690_MOESM1_ESM.pdf]

Supplemental information to the manuscript:

Transcriptomic analysis of a classical model of carbon catabolic regulation in *Streptomyces coelicolor*

Authors: Alba Romero-Rodríguez, Diana Rocha, Beatriz Ruiz-Villafan, Víctor Tierrafría, Romina Rodríguez-Sanoja, Daniel Segura and Sergio Sánchez

-
- *Corresponding author: Sergio Sánchez e-mail: sersan@biomedicas.unam.mx
- Tel.: 525556229199 Fax: 525556228920

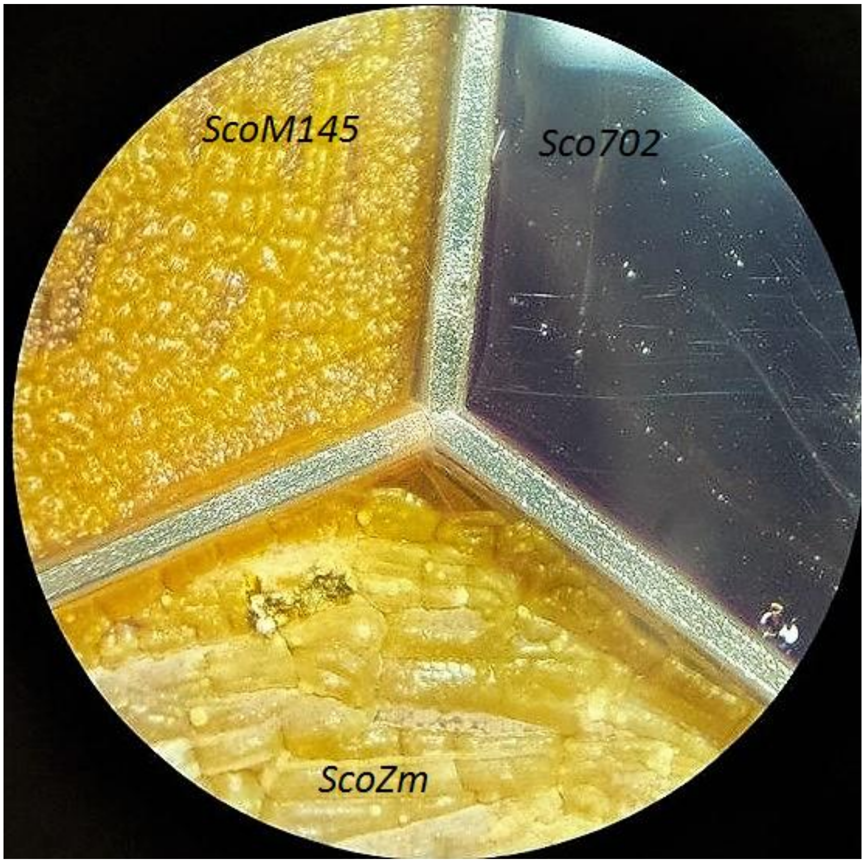

Figure S1. Qualitative visualization of Glk activity on BPG Agar medium after 48 h incubation. Strains: *Sco*M145, *ScoZm* (∆*scoglk*/*glk* from *Z. mobilis*) and *Sco*702 (∆*glk*).

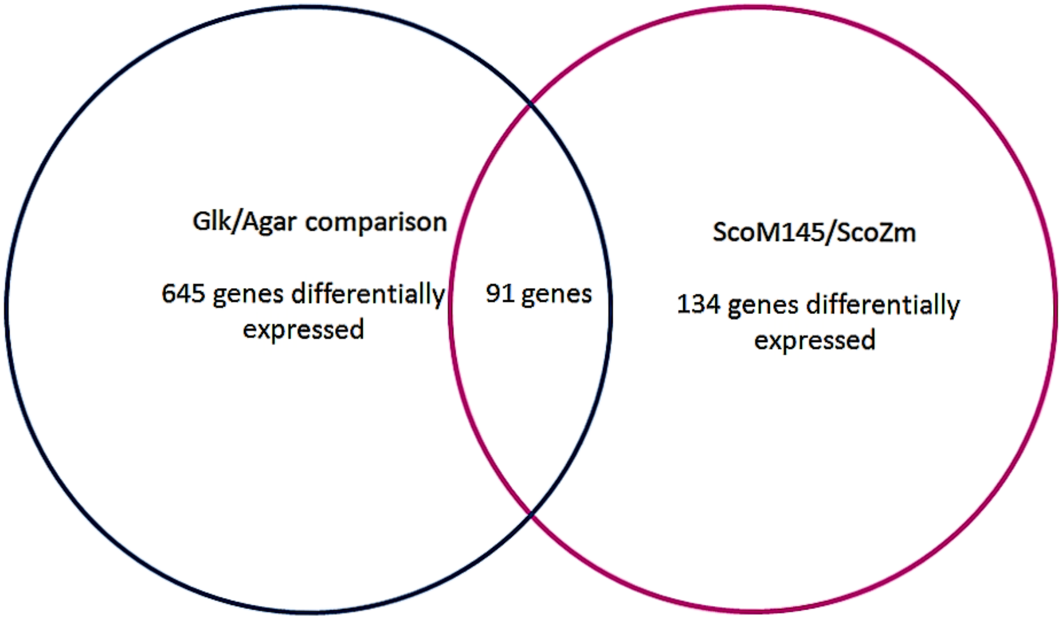

Fig. S2. Diagram summarizing the microarrays output and its intersection. In the Glc/ Agar comparison, 91 differentially expressed genes were detected, while in the Glk response (ScoM145/ScoZm), 134 differentially expressed genes were identified. From the total of differentially expressed genes in *Sco*M145/*ScoZm*, 91 were included in Glc/Agar.

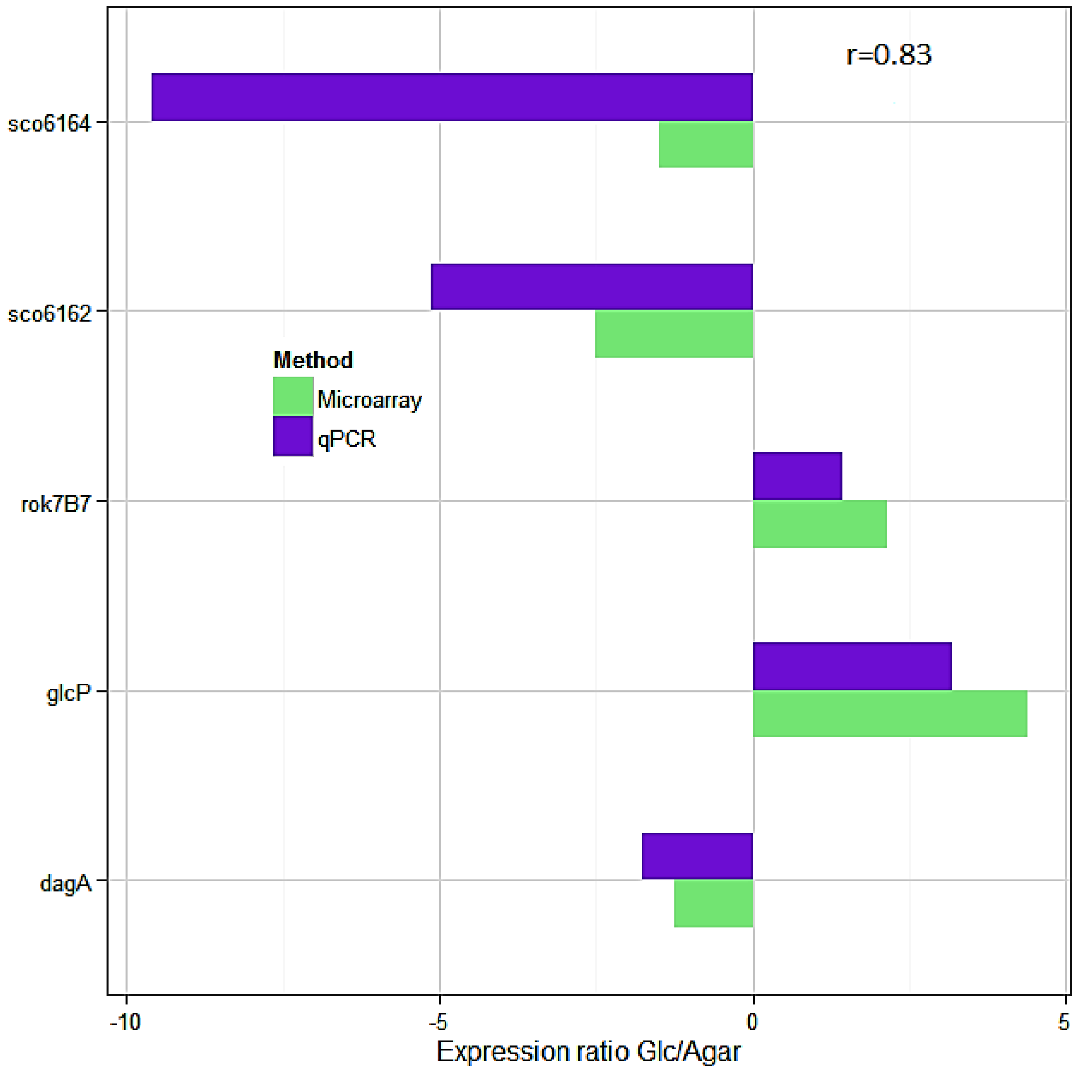

Figure S3. RT-qPCR validation of selected differentially expressed genes in Gluc/Agar (purple bars), compared to their relative transcript profile (green bars). The comparison between these conditions is indicated as ratio of Glc/Agar. Results are given as log2 ratio, thus a positive number indicates up-regulation and negative numbers down-regulation, respectively. r= Pearson correlation.

Table S1. Complete dataset of differentially expressed genes at statistically significant levels (pfp values ≤0.15) of *S. coelicolor* cells grown in 0.5% glucose+ 0.5% agar, relative to cells grown in 1% agar

**Gene name**	**Rank of down-regulated genes**	**pfp value**	**Log(2) Average**	**GO Biological Process / Molecular Function**	**Product/Function**
SCO0010	328	0.11104	-1.08		SCJ30.05; hypothetical protein (secreted protein); [GeneDB:SCO0010] [NCBI-GI:21218592] [NCBI-GeneID:1095439] [UniProt:Q9S1Y4]
SCO0043	306	0.09621	-1.10	metabolic process, catalytic activity	SCJ4.24c; hypothetical protein; [GeneDB:SCO0043] [NCBI-GI:21218622] [NCBI-GeneID:1095476] [UniProt:Q9S1U9]
SCO0090	336	0.11688	-1.00		SCJ11.19c; transposase; [GeneDB:SCO0090] [NCBI-GI:21218659] [NCBI-GeneID:1095518] [UniProt:Q9RI85]
SCO0108	153	0.02745	-1.23		SCJ11.37; hypothetical protein; [GeneDB:SCO0108] [NCBI-GI:21218672] [NCBI-GeneID:1095533] [UniProt:Q9RI69]
SCO0141	320	0.10703	-1.12		SCJ33.05c; calcium-binding protein; [GeneDB:SCO0141] [NCBI-GI:21218700] [NCBI-GeneID:1095565] [UniProt:Q9RIX2]
SCO0237	97	0.01175	-1.87	metabolic process, catalytic activity	SCJ9A.16; oxidoreductase; [GeneDB:SCO0237] [NCBI-GI:21218790] [NCBI-GeneID:1095661] [UniProt:Q9S1Q4]
SCO0256	143	0.02538	-1.52	metabolic process, catalytic activity	SCF20.02; short chain oxidoreductase; [GeneDB:SCO0256] [NCBI-GI:21218808] [NCBI-GeneID:1095680] [UniProt:Q9S2E4]
SCO0257	43	0.00279	-2.32	metabolic process, catalytic activity	SCF20.03; hypothetical protein; K07045; [GeneDB:SCO0257] [NCBI-GI:21218809] [NCBI-GeneID:1095681] [UniProt:Q9S2E3]
SCO0258	236	0.05818	-1.28		SCF20.04; hypothetical protein; [GeneDB:SCO0258] [NCBI-GI:21218810] [NCBI-GeneID:1095682] [UniProt:Q9S2E2]
SCO0268	72	0.00722	-1.95		SCF1.10; hypothetical protein; [GeneDB:SCO0268] [NCBI-GI:21218819] [NCBI-GeneID:1095692] [UniProt:Q9RK94]
SCO0315	66	0.00485	-1.83	metabolic process, catalytic activity	SC5G9.24c; decarboxylase; [GeneDB:SCO0315] [NCBI-GI:21218863] [NCBI-GeneID:1095739] [UniProt:Q9RL03]
SCO0381	226	0.05350	-1.22	metabolic process, catalytic activity	SCF62.07; glycosyl transferase; [GeneDB:SCO0381] [NCBI-GI:21218924] [NCBI-GeneID:1095804] [UniProt:Q9RJK7]
SCO0382	164	0.03091	-1.39	metabolic process, catalytic activity, nucleotide binding	SCF62.08; UDP-glucose/GDP-mannose family dehydrogenase (putative secreted protein) [EC:1.1.1.132]; K00066 GDPmannose 6-dehydrogenase; [GeneDB:SCO0382] [NCBI-GI:21218925] [NCBI-GeneID:1095805] [UniProt:Q9RJK6]

SCO0383	135	0.02422	-1.51	metabolic process	SCF62.09; hypothetical protein; [GeneDB:SCO0383] [NCBI-GI:21218926] [NCBI-GeneID:1095806] [UniProt:Q9RJK5]
SCO0384	359	0.13836	-0.99	metabolic process, catalytic activity, metal ion binding	SCF62.10; hypothetical protein; [GeneDB:SCO0384] [NCBI-GI:21218927] [NCBI-GeneID:1095807] [UniProt:Q9RJK4]
SCO0385	312	0.10333	-1.08	metabolic process	SCF62.11; hypothetical protein; [GeneDB:SCO0385] [NCBI-GI:21218928] [NCBI-GeneID:1095808] [UniProt:Q9RJK3]
SCO0386	299	0.09508	-1.09	metabolic process, catalytic activity, nucleotide binding	SCF62.12; putative asparagine synthetase [EC:6.3.5.4]; K01953 asparagine synthase (glutamine-hydrolysing); [GeneDB:SCO0386] [NCBI-GI:21218929] [NCBI-GeneID:1095809] [UniProt:Q9RJK2]
SCO0387	185	0.03897	-1.36	metabolic process, catalytic activity, nucleotide binding	SCF62.13; bi-domain oxidoreductase; [GeneDB:SCO0387] [NCBI-GI:21218930] [NCBI-GeneID:1095810] [UniProt:Q9RJK1]
SCO0388	339	0.11817	-1.04	metabolic process, catalytic activity	SCF62.14; hypothetical protein; [GeneDB:SCO0388] [NCBI-GI:21218931] [NCBI-GeneID:1095811] [UniProt:Q9RJK0]
SCO0389	148	0.02615	-1.46		SCF62.15; lipoprotein; [GeneDB:SCO0389] [NCBI-GI:21218932] [NCBI-GeneID:1095812] [UniProt:Q9RJJ9]
SCO0390	215	0.04865	-1.27		SCF62.16; hypothetical protein; [GeneDB:SCO0390] [NCBI-GI:21218933] [NCBI-GeneID:1095813] [UniProt:Q9RJJ8]
SCO0391	316	0.10449	-1.08	metabolic process, catalytic activity	SCF62.17; transferase; [GeneDB:SCO0391] [NCBI-GI:21218934] [NCBI-GeneID:1095814] [UniProt:Q9RJJ7]
SCO0392	103	0.01320	-1.73	metabolic process, catalytic activity	SCF62.18; methyltransferase; [GeneDB:SCO0392] [NCBI-GI:21218935] [NCBI-GeneID:1095815] [UniProt:Q9RJJ6]
SCO0393	84	0.00929	-1.83	metabolic process, catalytic activity	SCF62.19; putative transferase [EC:2.7.7.33]; K00978 glucose-1-phosphate cytidylyltransferase; [GeneDB:SCO0393] [NCBI-GI:21218936] [NCBI-GeneID:1095816] [UniProt:Q9RJJ5]
SCO0394	79	0.00886	-1.86		SCF62.20; hypothetical protein; [GeneDB:SCO0394] [NCBI-GI:21218937] [NCBI-GeneID:1095817] [UniProt:Q9RJJ4]
SCO0395	69	0.00580	-1.99	metabolic process, catalytic activity	SCF62.21; epimerase/dehydratase; [GeneDB:SCO0395] [NCBI-GI:21218938] [NCBI-GeneID:1095818] [UniProt:Q9RJJ3]
SCO0396	59	0.00356	-2.16	metabolic process, catalytic activity	SCF62.22; hypothetical protein; [GeneDB:SCO0396] [NCBI-GI:21218939] [NCBI-GeneID:1095819] [UniProt:Q9RJJ2]
SCO0397	337	0.11706	-1.05		SCF62.23; integral membrane protein; [GeneDB:SCO0397] [NCBI-GI:21218940] [NCBI-GeneID:1095820] [UniProt:Q9RJJ1]
SCO0398	105	0.01343	-1.77	metabolic process,	SCF62.24; glycosyl transferase; [GeneDB:SCO0398] [NCBI-GI:21218941] [NCBI-

				catalytic activity	GeneID:1095821] [UniProt:Q9RJJ0]
SCO0399	120	0.01717	-1.72		SCF62.25; hypothetical protein; [GeneDB:SCO0399] [NCBI-GI:21218942] [NCBI-GeneID:1095822] [UniProt:Q9RJI9]
SCO0400	129	0.01907	-1.68	metabolic process, catalytic activity	SCF62.26; putative epimerase [EC:5.1.3.13]; K01790 dTDP-4-dehydrorhamnose 3.5-epimerase; [GeneDB:SCO0400] [NCBI-GI:21218943] [NCBI-GeneID:1095823] [UniProt:Q9RJI8]
SCO0401	265	0.07038	-1.23	metabolic process, catalytic activity	SCF62.27; glutamate-1-semialdehyde 2.1-aminomutase; [GeneDB:SCO0401] [NCBI-GI:21218944] [NCBI-GeneID:1095824] [UniProt:Q9RJI7]
SCO0441	295	0.08678	-1.15	metabolic process, catalytic activity	SCF51A.19; hypothetical protein; K07160; [GeneDB:SCO0441] [NCBI-GI:21218983] [NCBI-GeneID:1095864] [UniProt:Q9RL45]
SCO0572	119	0.01706	-1.81	transporter activity	SC5G5.04c; putative cytosine permease; K03457 nucleobase:cation symporter-1. NCS1 family; [GeneDB:SCO0572] [NCBI-GI:21219106] [NCBI-GeneID:1095995] [UniProt:Q93RZ8]
SCO0584	80	0.00913	-1.85	metabolic process, catalytic activity, metal ion binding	SCF55.08c; cytochrome P450; [GeneDB:SCO0584] [NCBI-GI:21219117] [NCBI-GeneID:1096007] [UniProt:Q9RJQ6]
SCO0585	60	0.00350	-2.07		cvnD11. SCF55.09c; ATP/GTP binding protein; [GeneDB:SCO0585] [NCBI-GI:21219118] [NCBI-GeneID:1096008] [UniProt:Q9RJQ5]
SCO0586	93	0.00989	-1.79		cvnC11. SCF55.10c; hypothetical protein; [GeneDB:SCO0586] [NCBI-GI:21219119] [NCBI-GeneID:1096009] [UniProt:Q9RJQ4]
SCO0587	195	0.04226	-1.34		cvnB11. SCF55.11c; hypothetical protein; [GeneDB:SCO0587] [NCBI-GI:21219120] [NCBI-GeneID:1096010] [UniProt:Q9RJQ3]
SCO0588	139	0.02446	-1.57	metabolic process, catalytic activity, nucleotide binding	cvnA11. SCF55.12c; sensor kinase; [GeneDB:SCO0588] [NCBI-GI:21219121] [NCBI-GeneID:1096011] [UniProt:Q9RJQ2]
SCO0644	39	0.00282	-2.59		SCF91.04c.; membrane protein; [GeneDB:SCO0644] [NCBI-GI:21219174] [NCBI-GeneID:1096067] [UniProt:Q9RJC0]
SCO0682	258	0.06725	-1.25	DNA Binding	SCF15.03c; hypothetical protein; [GeneDB:SCO0682] [NCBI-GI:21219211] [NCBI-GeneID:1096105] [UniProt:Q9RK34]
SCO0683	325	0.10751	-1.09		SCF15.04c; hypothetical protein; K07131; [GeneDB:SCO0683] [NCBI-GI:21219212] [NCBI-GeneID:1096106] [UniProt:Q9RK33]
SCO0684	296	0.08875	-1.12		SCF15.05c; hypothetical protein; [GeneDB:SCO0684] [NCBI-GI:21219213] [NCBI-GeneID:1096107] [UniProt:Q9RK32]
SCO0685	281	0.07972	-1.19		SCF15.06c; hypothetical protein; [GeneDB:SCO0685] [NCBI-GI:21219214] [NCBI-GeneID:1096108] [UniProt:Q9RK31]

SCO0732	99	0.01202	-1.78	metabolic process, catalytic activity	3SC5B7.10; secreted protease; [GeneDB:SCO0732] [NCBI-GI:21219257] [NCBI-GeneID:1096155] [UniProt:Q9EWT9]
SCO0746	372	0.14567	-1.03	metabolic process, catalytic activity, metal ion binding	SCF81.05; serine/threonine protein kinase (putative secreted protein); [GeneDB:SCO0746] [NCBI-GI:21219270] [NCBI-GeneID:1096169] [UniProt:Q9RJE6]
SCO0752	37	0.00297	-2.51	metabolic process, catalytic activity	salO. SCF81.11c; protease precursor; [GeneDB:SCO0752] [NCBI-GI:21219276] [NCBI-GeneID:1096175] [UniProt:Q9RJE0]
SCO0930	8	0.00000	-3.29		SCM10.18c; lipoprotein; [GeneDB:SCO0930] [NCBI-GI:21219449] [NCBI-GeneID:1096353] [UniProt:Q9RCY0]
SCO0932	220	0.05118	-1.40		SCM10.20; integral membrane protein; [GeneDB:SCO0932] [NCBI-GI:21219451] [NCBI-GeneID:1096355] [UniProt:Q9RCX8]
SCO0944	362	0.14028	-1.11		SCM10.32; hypothetical protein; [GeneDB:SCO0944] [NCBI-GI:21219463] [NCBI-GeneID:1096367] [UniProt:Q9RCW6]
SCO0955	42	0.00286	-2.27		SCM11.10c; hypothetical protein; [GeneDB:SCO0955] [NCBI-GI:21219473] [NCBI-GeneID:1096378] [UniProt:Q9RIV1]
SCO0961	253	0.06312	-1.20	metabolic process, catalytic activity, nucleotide binding	glgC. SCM11.16c; glucose-1-phosphate adenylyltransferase [EC:2.7.7.27]; K00975 glucose-1-phosphate adenylyltransferase; [GeneDB:SCO0961] [NCBI-GI:21219479] [NCBI-GeneID:1096384] [UniProt:P72394]
SCO0992	331	0.11628	-1.11	metabolic process, catalytic activity	2SCG2.05; cysteine synthase; [GeneDB:SCO0992] [NCBI-GI:21219509] [NCBI-GeneID:1096415] [UniProt:Q9EX46]
SCO1064	371	0.14606	-0.90	transporter activity	SCG22.10c; putative sugar transport integral membrane protein; K02025 multiple sugar transport system permease protein; [GeneDB:SCO1064] [NCBI-GI:21219579] [NCBI-GeneID:1096487] [UniProt:Q9K435]
SCO1087	29	0.00138	-2.48		2SCG4.03c; putative aldolase [EC:4.1.2.5]; K01620 threonine aldolase; [GeneDB:SCO1087] [NCBI-GI:21219602] [NCBI-GeneID:1096510] [UniProt:Q9K3R1]
SCO1088	20	0.00100	-2.80	metabolic process, catalytic activity	2SCG4.04c; oxidoreductase; [GeneDB:SCO1088] [NCBI-GI:21219603] [NCBI-GeneID:1096511] [UniProt:Q9K3R0]
SCO1089	7	0.00000	-3.35		2SCG4.05c; hypothetical protein 2SCG405c; [GeneDB:SCO1089] [NCBI-GI:21219604] [NCBI-GeneID:1096512] [UniProt:Q9K3Q9]
SCO1113	330	0.11355	-0.99		2SCG38.06; hypothetical protein; [GeneDB:SCO1113] [NCBI-GI:21219627] [NCBI-GeneID:1096536] [UniProt:Q9EX13]
SCO1121	19	0.00105	-2.90		2SCG38.14; secreted protein; [GeneDB:SCO1121] [NCBI-GI:21219635] [NCBI-GeneID:1096544] [UniProt:Q9EX05]
SCO1157	137	0.02401	-1.59		cvnD3. SCG8A.11c; ATP/GTP binding protein; [GeneDB:SCO1157] [NCBI-GI:21219670] [NCBI-GeneID:1096580] [UniProt:Q9KZI8]

SCO1158	147	0.02578	-1.60		cvnC3. SCG8A.12c; hypothetical protein; [GeneDB:SCO1158] [NCBI-GI:21219671] [NCBI-GeneID:1096581] [UniProt:Q9KZI7]
SCO1159	142	0.02507	-1.59		cvnB3. SCG8A.13c; hypothetical protein; [GeneDB:SCO1159] [NCBI-GI:21219672] [NCBI-GeneID:1096582] [UniProt:Q9KZI6]
SCO1160	98	0.01163	-1.80		cvnA3. SCG8A.14c; hypothetical protein; [GeneDB:SCO1160] [NCBI-GI:21219673] [NCBI-GeneID:1096583] [UniProt:Q9KZI5]
SCO1161	150	0.02627	-1.58		SCG8A.15c; integral membrane protein; [GeneDB:SCO1161] [NCBI-GI:21219674] [NCBI-GeneID:1096584] [UniProt:Q9KZI4]
SCO1163	348	0.12328	-1.02		SCG8A.17c; hypothetical protein; [GeneDB:SCO1163] [NCBI-GI:21219676] [NCBI-GeneID:1096586] [UniProt:Q9KZI2]
SCO1174	366	0.14227	-1.04	metabolic process, catalytic activity	SCG11A.05. thcA; aldehyde dehydrogenase [EC:1.2.1.3]; K00128 aldehyde dehydrogenase (NAD+); [GeneDB:SCO1174] [NCBI-GI:21219685] [NCBI-GeneID:1096597] [UniProt:Q9RJZ6]
SCO1189	378	0.14902	-1.02		SCG11A.20; hypothetical protein; [GeneDB:SCO1189] [NCBI-GI:21219700] [NCBI-GeneID:1096612] [UniProt:Q9RJY1]
SCO1241	363	0.14080	-1.03		2SCG1.16c; hypothetical protein; [GeneDB:SCO1241] [NCBI-GI:21219750] [NCBI-GeneID:1096664] [UniProt:Q9FCC6]
SCO1294	12	0.00083	-3.04	metabolic process, catalytic activity,	SCBAC36F5.05c; cystathionine gamma-synthase; [GeneDB:SCO1294] [NCBI-GI:21219801] [NCBI-GeneID:1096717] [UniProt:Q93IX6]
SCO1328	332	0.11645	-1.15		2SCG61.10c; hypothetical protein; [GeneDB:SCO1328] [NCBI-GI:21219834] [NCBI-GeneID:1096751] [UniProt:Q9K405]
SCO1329	261	0.06969	-1.13		2SCG61.11c; hypothetical protein; [GeneDB:SCO1329] [NCBI-GI:21219835] [NCBI-GeneID:1096752] [UniProt:Q9K404]
SCO1330	239	0.05904	-1.28	metabolic process, catalytic activity	2SCG61.12c; fatty acid CoA ligase; [GeneDB:SCO1330] [NCBI-GI:21219836] [NCBI-GeneID:1096753] [UniProt:Q9K403]
SCO1356	75	0.00760	-2.14	metabolic process, transport	2SCG61.38; iron sulphur protein (secreted protein); [GeneDB:SCO1356] [NCBI-GI:21219862] [NCBI-GeneID:1096779] [UniProt:Q9K3X8]
SCO1357	15	0.00067	-3.09	metabolic process, catalytic activity, nucleotide binding	2SCG61.39; hypothetical protein; [GeneDB:SCO1357] [NCBI-GI:21219863] [NCBI-GeneID:1096780] [UniProt:Q9K3X7]
SCO1359	194	0.04216	-1.31		2SCG61.41. SC10A9.01; integral membrane protein; [GeneDB:SCO1359] [NCBI-GI:32141140] [NCBI-GeneID:1096782] [UniProt:Q8CK31]
SCO1378	319	0.10661	-1.13	metabolic process, catalytic activity	SC10A9.20c; putative glycine dehydrogenase [EC:1.4.4.2]; K00282 glycine dehydrogenase subunit 1; K00283 glycine dehydrogenase subunit 2; [GeneDB:SCO1378] [NCBI-GI:21219883] [NCBI-GeneID:1096801] [UniProt:Q9AK84]

SCO1411	91	0.00923	-1.96	transporter activity	SC6D7.28c; transmembrane transport protein; [GeneDB:SCO1411] [NCBI-GI:21219914] [NCBI-GeneID:1096837] [UniProt:Q9RKX0]
SCO1412	55	0.00327	-2.24		SC6D7.27c; hypothetical protein; [GeneDB:SCO1412] [NCBI-GI:21219915] [NCBI-GeneID:1096838] [UniProt:Q9RKX1]
SCO1427	315	0.10432	-1.15		SC6D7.12c; hypothetical protein; [GeneDB:SCO1427] [NCBI-GI:21219930] [NCBI-GeneID:1096853] [UniProt:Q9RKY6]
SCO1428	121	0.01711	-1.73	metabolic process, catalytic activity, nucleotide binding	acd; acyl-CoA dehydrogenase [EC:1.3.99.-]; K00257; [GeneDB:SCO1428] [NCBI-GI:21219931] [NCBI-GeneID:1096854] [UniProt:Q9RKY7]
SCO1474	373	0.14552	-1.03		SCL6.31; hypothetical protein; [GeneDB:SCO1474] [NCBI-GI:21219976] [NCBI-GeneID:1096900] [UniProt:Q9L0Y5]
SCO1541	175	0.03680	-1.45		SCL2.31; regulator; [GeneDB:SCO1541] [NCBI-GI:21220041] [NCBI-GeneID:1096967] [UniProt:Q9L268]
SCO1563	65	0.00462	-2.00	metabolic process, DNA binding	SCL11.19c; acetyltransferase; [GeneDB:SCO1563] [NCBI-GI:21220062] [NCBI-GeneID:1096994] [UniProt:Q9L1B9]
SCO1573	18	0.00111	-2.96	metabolic process, catalytic activity	SCL24.09; oxidoreductase (membrane protein); [GeneDB:SCO1573] [NCBI-GI:21220071] [NCBI-GeneID:1097004] [UniProt:Q9L1A8]
SCO1574	216	0.04926	-1.33		SCL24.10; hypothetical protein; [GeneDB:SCO1574] [NCBI-GI:21220072] [NCBI-GeneID:1097005] [UniProt:Q9L1A7]
SCO1575	96	0.01010	-1.80	cell organization and biogenesis, metabolic process	SCL24.11; putative thiamine biosynthesis lipoprotein precursor; K03734 thiamine biosynthesis lipoprotein; [GeneDB:SCO1575] [NCBI-GI:21220073] [NCBI-GeneID:1097006] [UniProt:Q9L1A6]
SCO1590	346	0.12283	-1.03	metabolic process	SCI35.12c; secreted protein; [GeneDB:SCO1590] [NCBI-GI:21220087] [NCBI-GeneID:1097021] [UniProt:O88050]
SCO1626	157	0.02911	-1.63	metabolic process, catalytic activity, metal ion binding	SCI41.09c; cytochrome P450; [GeneDB:SCO1626] [NCBI-GI:21220122] [NCBI-GeneID:1097057] [UniProt:Q9RJ75]
SCO1627	85	0.00918	-1.91		cvnD9. SCI41.10c; ATP-GTP binding protein; [GeneDB:SCO1627] [NCBI-GI:21220123] [NCBI-GeneID:1097058] [UniProt:Q9RJ74]
SCO1628	172	0.03227	-1.55		cvnC9. SCI41.11c; hypothetical protein; [GeneDB:SCO1628] [NCBI-GI:21220124] [NCBI-GeneID:1097059] [UniProt:Q9RJ73]
SCO1629	108	0.01676	-1.78		cvnB9. SCI41.12c; hypothetical protein; [GeneDB:SCO1629] [NCBI-GI:21220125] [NCBI-GeneID:1097060] [UniProt:Q9RJ72]

SCO1630	127	0.01874	-1.78		cvnA9. SCI41.13c; integral membrane protein; [GeneDB:SCO1630] [NCBI-GI:21220126] [NCBI-GeneID:1097061] [UniProt:Q9RJ71]
SCO1660	367	0.14253	-0.98	metabolic process, catalytic activity	glpK. SCI52.02; putative glycerol kinase [EC:2.7.1.30]; K00864 glycerol kinase; [GeneDB:SCO1660] [NCBI-GI:21220156] [NCBI-GeneID:1097091] [UniProt:Q9ADA7]
SCO1699	243	0.05922	-1.27	metabolic process, regulation of biological process, DNA Binding	SCI30A.20c; transcriptional regulator; [GeneDB:SCO1699] [NCBI-GI:21220194] [NCBI-GeneID:1097130] [UniProt:Q9S253]
SCO1700	177	0.03712	-1.45		SCI30A.21c; hypothetical protein; [GeneDB:SCO1700] [NCBI-GI:21220195] [NCBI-GeneID:1097131] [UniProt:Q9S252]
SCO1705	227	0.05335	-1.22	metabolic process, catalytic activity, metal ion binding	SCI30A.26c; alcohol dehydrogenase (zinc-binding); [GeneDB:SCO1705] [NCBI-GI:21220200] [NCBI-GeneID:1097136] [UniProt:Q9S247]
SCO1706	200	0.04320	-1.29	metabolic process, catalytic activity	SCI30A.27c; putative aldehyde dehydrogenase [EC:1.2.1.3]; K00128 aldehyde dehydrogenase (NAD+); [GeneDB:SCO1706] [NCBI-GI:21220201] [NCBI-GeneID:1097137] [UniProt:Q9S246]
SCO1722	377	0.14878	-0.96		SCI11.11c; hypothetical protein; [GeneDB:SCO1722] [NCBI-GI:21220216] [NCBI-GeneID:1097153] [UniProt:Q9S2A8]
SCO1773	32	0.00125	-2.46	metabolic process, catalytic activity	SCI51.13c; putative L-alanine dehydrogenase [EC:1.4.1.1]; K00259 alanine dehydrogenase; [GeneDB:SCO1773] [NCBI-GI:21220265] [NCBI-GeneID:1097204] [UniProt:Q9S227]
SCO1810	250	0.06288	-1.22		SCI28.04c; hypothetical protein; [GeneDB:SCO1810] [NCBI-GI:21220300] [NCBI-GeneID:1097244] [UniProt:Q9S279]
SCO1823	107	0.01589	-1.63		SCI8.08c; integral membrane protein; [GeneDB:SCO1823] [NCBI-GI:21220312] [NCBI-GeneID:1097257] [UniProt:Q9RJ45]
SCO1860	178	0.03713	-1.57		SCI39.07c; secreted protein; [GeneDB:SCO1860] [NCBI-GI:21220348] [NCBI-GeneID:1097294] [UniProt:Q93RW6]
SCO1903	217	0.05120	-1.23	stimulus response	SCI7.21; transport associated protein; [GeneDB:SCO1903] [NCBI-GI:21220390] [NCBI-GeneID:1097337] [UniProt:Q9X9W9]
SCO2016	384	0.15010	-0.94	metabolic process, catalytic activity	SC7H2.30c; monooxygenase; [GeneDB:SCO2016] [NCBI-GI:21220498] [NCBI-GeneID:1097450] [UniProt:Q9S2I7]
SCO2113	256	0.06652	-1.36	catalytic activity, metal ion binding	bfr. SC6E10.07; putative bacterioferritin; K03594 bacterioferritin; [GeneDB:SCO2113] [NCBI-GI:21220591] [NCBI-GeneID:1097547] [UniProt:Q9S2N0]
SCO2207	35	0.00171	-2.48		SC10B7.02; hypothetical secreted protein; [GeneDB:SCO2207] [NCBI-GI:21220679] [NCBI-GeneID:1097640] [UniProt:Q9X955]
SCO2209	203	0.04365	-1.33	metabolic process,	SC10B7.04; transcriptional regulator; [GeneDB:SCO2209] [NCBI-GI:21220681]

				regulation of biological process	[NCBI-GeneID:1097642] [UniProt:Q9X957]
SCO2217	4	0.00000	-3.91		SC10B7.12; secreted protein; [GeneDB:SCO2217] [NCBI-GI:21220689] [NCBI-GeneID:1097650] [UniProt:Q9KZ20]
SCO2218	68	0.00559	-2.15		SC10B7.13c; lipoprotein; [GeneDB:SCO2218] [NCBI-GI:21220690] [NCBI-GeneID:1097651] [UniProt:Q9KZ19]
SCO2229	327	0.11064	-1.03	transporter activity	malG. SC10B7.24c; putative maltose permease; K10110 maltose/maltodextrin transport system permease protein; [GeneDB:SCO2229] [NCBI-GI:21220701] [NCBI-GeneID:1097662] [UniProt:Q9KZ08]
SCO2230	311	0.10122	-1.06	transporter activity	malF. SC10B7.25c; putative maltose permease; K10109 maltose/maltodextrin transport system permease protein; [GeneDB:SCO2230] [NCBI-GI:21220702] [NCBI-GeneID:1097663] [UniProt:Q9KZ07]
SCO2408	88	0.00932	-1.91	metabolic process, catalytic activity	SC4A7.36; aminotransferase; [GeneDB:SCO2408] [NCBI-GI:21220875] [NCBI-GeneID:1097842] [UniProt:Q9RDM9]
SCO2494	23	0.00130	-2.71	metabolic process, catalytic activity, nucleotide binding	SC7A8.33c; putative pyruvate phosphate dikinase [EC:2.7.9.1]; K01006 pyruvate.orthophosphate dikinase; [GeneDB:SCO2494] [NCBI-GI:21220957] [NCBI-GeneID:1097928] [UniProt:Q9L2C5]
SCO2495	50	0.00280	-2.34		SC7A8.34. SCC82.01; hypothetical protein; [GeneDB:SCO2495] [NCBI-GI:32141171] [NCBI-GeneID:1097929] [UniProt:Q8CJZ4]
SCO2529	78	0.00859	-1.82	metabolic process, catalytic activity	SCC117.02; metalloprotease [EC:3.4.24.-]; K01417; [GeneDB:SCO2529] [NCBI-GI:21220989] [NCBI-GeneID:1097963] [UniProt:Q9L2L8]
SCO2530	83	0.00928	-1.80		SCC117.03; hypothetical protein; [GeneDB:SCO2530] [NCBI-GI:21220990] [NCBI-GeneID:1097964] [UniProt:Q9L2L7]
SCO2560	297	0.08923	-1.15	metabolic process, catalytic activity, nucleotide binding	SCC77.27c; hypothetical protein; [GeneDB:SCO2560] [NCBI-GI:21221019] [NCBI-GeneID:1097994] [UniProt:Q9RDD1]
SCO2572	262	0.06966	-1.22		SCC123.10; integral membrane protein; [GeneDB:SCO2572] [NCBI-GI:21221031] [NCBI-GeneID:1098006] [UniProt:Q9RDL4]
SCO2573	134	0.02366	-1.60	metabolic process, catalytic activity, metal ion binding	SCC123.11; oxidoreductase; [GeneDB:SCO2573] [NCBI-GI:21221032] [NCBI-GeneID:1098007] [UniProt:Q9RDL3]
SCO2717	361	0.13920	-1.13		SCC46.02c; small membrane protein; [GeneDB:SCO2717] [NCBI-GI:21221171] [NCBI-GeneID:1098151] [UniProt:Q9L1J9]
SCO2718	240	0.05892	-1.20		SCC46.03c; secreted protein; [GeneDB:SCO2718] [NCBI-GI:21221172] [NCBI-GeneID:1098152] [UniProt:Q9L1J8]

SCO2719	292	0.08421	-1.07		SCC46.04; secreted protein; [GeneDB:SCO2719] [NCBI-GI:21221173] [NCBI-GeneID:1098153] [UniProt:Q9L1J7]
SCO2726	62	0.00387	-2.06	metabolic process, catalytic activity	msdA. SCC46.11c; methylmalonic acid semialdehyde dehydrogenase [EC:1.2.1.27]; K00140 methylmalonate-semialdehyde dehydrogenase; [GeneDB:SCO2726] [NCBI-GI:21221180] [NCBI-GeneID:1098160] [UniProt:Q9L1J1]
SCO2727	140	0.02507	-1.59		SCC46.12c; hypothetical protein; [GeneDB:SCO2727] [NCBI-GI:21221181] [NCBI-GeneID:1098161] [UniProt:Q9L1J0]
SCO2770	357	0.13555	-0.95	metabolic process, catalytic activity, metal ion binding	SCC105.01c. SCC57A.41c. speB; agmatinase [EC:3.5.3.11]; K01480 agmatinase; [GeneDB:SCO2770] [NCBI-GI:32141181] [NCBI-GeneID:1098204] [UniProt:Q8CJY5]
SCO2774	280	0.07911	-1.12	metabolic process, catalytic activity, nucleotide binding	acdH2. SCC105.05c; acyl-CoA dehydrogenase [EC:1.3.99.3]; K00249 acyl-CoA dehydrogenase; [GeneDB:SCO2774] [NCBI-GI:21221225] [NCBI-GeneID:1098208] [UniProt:Q9L079]
SCO2776	22	0.00091	-2.83		accD1. SCC105.07; acetyl/propionyl CoA carboxylase. beta subunit [EC:6.4.1.3]; K01966 propionyl-CoA carboxylase beta chain; [GeneDB:SCO2776] [NCBI-GI:21221227] [NCBI-GeneID:1098210] [UniProt:Q9L077]
SCO2777	158	0.02918	-1.48	metabolic process, catalytic activity, metal ion binding	accC. SCC105.08; acetyl/propionyl CoA carboxylase alpha subunit [EC:6.3.4.14]; K01946 biotin carboxylase; [GeneDB:SCO2777] [NCBI-GI:21221228] [NCBI-GeneID:1098211] [UniProt:Q9L076]
SCO2778	34	0.00147	-2.61		hmgL. SCC105.09; hydroxymethylglutaryl-CoA lyase [EC:4.1.3.4]; K01640 hydroxymethylglutaryl-CoA lyase; [GeneDB:SCO2778] [NCBI-GI:21221229] [NCBI-GeneID:1098212] [UniProt:Q9L075]
SCO2779	3	0.00000	-3.90	metabolic process, catalytic activity, nucleotide binding	acdH. SCC105.10; acyl-CoA dehydrogenase; [GeneDB:SCO2779] [NCBI-GI:21221230] [NCBI-GeneID:1098213] [UniProt:Q9XCG6]
SCO2788	95	0.00989	-1.81		SCC105.19; hypothetical protein; [GeneDB:SCO2788] [NCBI-GI:21221239] [NCBI-GeneID:1098222] [UniProt:Q9L066]
SCO2789	100	0.01250	-1.85	metabolic process, catalytic activity	glmS2. SCC105.20; glucosamine-fructose-6-phosphate aminotransferase [EC:2.6.1.16]; K00820 glucosamine--fructose-6-phosphate aminotransferase (isomerizing); [GeneDB:SCO2789] [NCBI-GI:21221240] [NCBI-GeneID:1098223] [UniProt:Q9L065]
SCO2805	114	0.01632	-1.67		2SCC13.13; hypothetical protein; [GeneDB:SCO2805] [NCBI-GI:21221256] [NCBI-GeneID:1098239] [UniProt:Q9F3H6]
SCO2822	294	0.08673	-1.11	metabolic process,	SCBAC17F8.13c; decarboxylase; [GeneDB:SCO2822] [NCBI-GI:21221272] [NCBI-

				catalytic activity	GeneID:1098256] [UniProt:Q93J75]
SCO2823	279	0.07849	-1.11	metabolic process, catalytic activity	SCBAC17F8.14c; decarboxylase; [GeneDB:SCO2823] [NCBI-GI:21221273] [NCBI-GeneID:1098257] [UniProt:Q93J74]
SCO2826	228	0.05338	-1.30		SCBAC17F8.17; hypothetical protein; [GeneDB:SCO2826] [NCBI-GI:21221276] [NCBI-GeneID:1098260] [UniProt:Q93J71]
SCO2883	355	0.13251	-0.98	metabolic process, catalytic activity, metal ion binding	SCE6.20; cytochrome P450; [GeneDB:SCO2883] [NCBI-GI:21221332] [NCBI-GeneID:1098317] [UniProt:Q9KZR8]
SCO2953	161	0.02975	-1.48		SCE59.12c; hypothetical protein; [GeneDB:SCO2953] [NCBI-GI:21221398] [NCBI-GeneID:1098386] [UniProt:Q9L1U2]
SCO2954	28	0.00143	-2.60	metabolic process, regulation of biological process, DNA binding	SCE59.13c; putative RNA polymerase sigma factor; K03088 RNA polymerase sigma-70 factor. ECF subfamily; [GeneDB:SCO2954] [NCBI-GI:21221399] [NCBI-GeneID:1098387] [UniProt:Q9L1U1]
SCO2976	56	0.00357	-2.16		SCE50.04c; hypothetical protein; [GeneDB:SCO2976] [NCBI-GI:21221420] [NCBI-GeneID:1098409] [UniProt:Q9L059]
SCO2977	183	0.03874	-1.42		SCE50.05; hypothetical protein; [GeneDB:SCO2977] [NCBI-GI:21221421] [NCBI-GeneID:1098410] [UniProt:Q9L058]
SCO2979	254	0.06323	-1.24	transporter activity	SCE50.07; putative integral membrane transport protein; K10233 alpha-glucoside transport system permease protein; [GeneDB:SCO2979] [NCBI-GI:21221423] [NCBI-GeneID:1098412] [UniProt:Q9L056]
SCO2980	231	0.05398	-1.27	transporter activity	SCE50.08; putative integral membrane transport protein; K10234 alpha-glucoside transport system permease protein; [GeneDB:SCO2980] [NCBI-GI:21221424] [NCBI-GeneID:1098413] [UniProt:Q9L055]
SCO2999	40	0.00300	-2.22	metabolic process, catalytic activity	SCE33.01c. SCE99.06c; hypothetical protein; [GeneDB:SCO2999] [NCBI-GI:32141188] [NCBI-GeneID:1098432] [UniProt:Q8CJY0]
SCO3051	64	0.00406	-2.20	metabolic process, catalytic activity, nucleotide binding	fadE. SCBAC19G2.06c; acyl-CoA dehydrogenase [EC:1.3.99.-]; K00257; [GeneDB:SCO3051] [NCBI-GI:21221493] [NCBI-GeneID:1098484] [UniProt:Q7AKM9]
SCO3112	235	0.05511	-1.31		SCE41.21c; hypothetical protein; [GeneDB:SCO3112] [NCBI-GI:21221551] [NCBI-GeneID:1098546] [UniProt:Q9F2N8]
SCO3134	266	0.07034	-1.20	cell communication, metabolic process, DNA binding	SCE66.13c; two-component system response regulator; [GeneDB:SCO3134] [NCBI-GI:21221572] [NCBI-GeneID:1098568] [UniProt:Q9K3T0]

SCO3263	369	0.14268	-1.64		SCE39.13c; hypothetical protein; [GeneDB:SCO3263] [NCBI-GI:21221696] [NCBI-GeneID:1098697] [UniProt:Q9X8D6]
SCO3265	349	0.12307	-1.85		SCE39.15c; hypothetical protein; [GeneDB:SCO3265] [NCBI-GI:21221698] [NCBI-GeneID:1098699] [UniProt:Q9X8D8]
SCO3266	271	0.07111	-1.93		SCE39.16c; hypothetical protein; [GeneDB:SCO3266] [NCBI-GI:21221699] [NCBI-GeneID:1098700] [UniProt:Q9X8D9]
SCO3268	263	0.06981	-1.97		SCE39.18c; secreted protein; [GeneDB:SCO3268] [NCBI-GI:21221701] [NCBI-GeneID:1098702] [UniProt:Q9X8E1]
SCO3288	144	0.02521	-1.70		SCE15.05; integral membrane protein; [GeneDB:SCO3288] [NCBI-GI:21221720] [NCBI-GeneID:1098722] [UniProt:Q9X880]
SCO3289	89	0.00933	-2.02		SCE15.06; large membrane protein; [GeneDB:SCO3289] [NCBI-GI:21221721] [NCBI-GeneID:1098723] [UniProt:Q9X881]
SCO3290	141	0.02511	-1.77		SCE15.07; hypothetical protein; [GeneDB:SCO3290] [NCBI-GI:21221722] [NCBI-GeneID:1098724] [UniProt:Q9X882]
SCO3323	210	0.04610	-1.37	metabolic process, DNA binding	SCE68.21; putative RNA polymerase sigma factor; K03088 RNA polymerase sigma-70 factor.ECF subfamily; [GeneDB:SCO3323] [NCBI-GI:21221754] [NCBI-GeneID:1098757] [UniProt:Q9WX11]
SCO3328	181	0.03801	-1.34	metabolic process, DNA binding	SCE68.26c; hypothetical protein; [GeneDB:SCO3328] [NCBI-GI:21221759] [NCBI-GeneID:1098762] [UniProt:Q9WX06]
SCO3362	57	0.00351	-2.11	transporter activity	SCE94.13; hypothetical protein; [GeneDB:SCO3362] [NCBI-GI:21221791] [NCBI-GeneID:1098799] [UniProt:Q9X8L9]
SCO3365	188	0.04064	-1.31	metabolic process, DNA binding	SCE94.16c; hypothetical protein; [GeneDB:SCO3365] [NCBI-GI:21221794] [NCBI-GeneID:1098802] [UniProt:Q9X8M2]
SCO3471	169	0.03207	-1.24	metabolic process, catalytic activity	dagA. SCE65.07c; extracellular agarase precursor [EC:3.2.1.81]; K01219 agarase; [GeneDB:SCO3471] [NCBI-GI:21221895] [NCBI-GeneID:1098908] [UniProt:P07883]
SCO3472	128	0.01875	-1.82	metabolic process, DNA binding	SCE65.08c; transposase remnant; [GeneDB:SCO3472] [NCBI-GI:21221896] [NCBI-GeneID:1098909] [UniProt:Q9RKG5]
SCO3473	71	0.00732	-2.23		SCE65.09c; putative aldolase [EC:4.1.2.14 4.1.3.16]; K01625 2-dehydro-3-deoxyphosphogluconate aldolase; K01650 4-hydroxy-2-oxoglutarate aldolase; [GeneDB:SCO3473] [NCBI-GI:21221897] [NCBI-GeneID:1098910] [UniProt:Q9RKG4]
SCO3474	92	0.00913	-2.07		SCE65.10c; sugar kinase; [GeneDB:SCO3474] [NCBI-GI:21221898] [NCBI-GeneID:1098911] [UniProt:Q9RKG3]
SCO3475	67	0.00507	-2.24	metabolic process,	SCE65.11c; putative galactonate dehydratase protein [EC:4.2.1.6]; K01684

				catalytic activity	galactonate dehydratase; [GeneDB:SCO3475] [NCBI-GI:21221899] [NCBI-GeneID:1098912] [UniProt:Q9RKG2]
SCO3476	49	0.00286	-2.50		SCE65.12c; putative short-chain dehydrogenase [EC:1.1.1.125]; K00065 2-deoxy-D-gluconate 3-dehydrogenase; [GeneDB:SCO3476] [NCBI-GI:21221900] [NCBI-GeneID:1098913] [UniProt:Q9RKG1]
SCO3477	48	0.00271	-2.52	metabolic process, catalytic activity, metal ion binding	SCE65.13c; dehydrogenase; [GeneDB:SCO3477] [NCBI-GI:21221901] [NCBI-GeneID:1098914] [UniProt:Q9RKG0]
SCO3478	45	0.00267	-2.53		SCE65.14c; dehydrogenase; [GeneDB:SCO3478] [NCBI-GI:21221902] [NCBI-GeneID:1098915] [UniProt:Q9RKF9]
SCO3479	52	0.00327	-2.46	metabolic process, catalytic activity	SCE65.15c; putative beta-galatosidase [EC:3.2.1.23]; K01190 beta-galactosidase; [GeneDB:SCO3479] [NCBI-GI:21221903] [NCBI-GeneID:1098916] [UniProt:Q9RKF8]
SCO3480	38	0.00289	-2.55	metabolic process, catalytic activity, metal ion binding	SCE65.16c; putative racemase [EC:5.1.2.2]; K01781 mandelate racemase; [GeneDB:SCO3480] [NCBI-GI:21221904] [NCBI-GeneID:1098917] [UniProt:Q9RKF7]
SCO3481	51	0.00333	-2.43	metabolic process, catalytic activity	SCE65.17c; hypothetical protein; [GeneDB:SCO3481] [NCBI-GI:21221905] [NCBI-GeneID:1098918] [UniProt:Q9RKF6]
SCO3482	41	0.00293	-2.56	transporter activity	SCE65.18c; putative sugar-permease; K02026 multiple sugar transport system permease protein; [GeneDB:SCO3482] [NCBI-GI:21221906] [NCBI-GeneID:1098919] [UniProt:Q9RKF5]
SCO3483	14	0.00071	-3.09	transporter activity	SCE65.19c; putative integral membrane transport protein; K02025 multiple sugar transport system permease protein; [GeneDB:SCO3483] [NCBI-GI:21221907] [NCBI-GeneID:1098920] [UniProt:Q9RKF4]
SCO3484	25	0.00160	-2.85	transporter activity	SCE65.20c; putative secreted sugar-binding protein; K02027 multiple sugar transport system substrate-binding protein; [GeneDB:SCO3484] [NCBI-GI:21221908] [NCBI-GeneID:1098921] [UniProt:Q9RKF3]
SCO3486	149	0.02638	-1.51	metabolic process, catalytic activity, metal ion binding	SCE65.22; putative aldehyde dehydrogenase [EC:1.2.1.22]; K00138 lactaldehyde dehydrogenase; [GeneDB:SCO3486] [NCBI-GI:21221910] [NCBI-GeneID:1098923] [UniProt:Q9RKF1]
SCO3487	26	0.00154	-2.79		SCE65.23; hydrolase; [GeneDB:SCO3487] [NCBI-GI:21221911] [NCBI-GeneID:1098924] [UniProt:Q9RKF0]
SCO3527	192	0.04135	-1.23		SCE2.08; hypothetical protein; [GeneDB:SCO3527] [NCBI-GI:21221948] [NCBI-GeneID:1098963] [UniProt:Q9X8A3]

SCO3540	354	0.12972	-1.00		SCH5.02c. slpD; proteinase (secreted protein); [GeneDB:SCO3540] [NCBI-GI:21221959] [NCBI-GeneID:1098976] [UniProt:Q7AKL3]
SCO3603	308	0.09789	-1.03	transporter activity	SC66T3.14c; putative Na(+)/H(+) antiporter; K03316 monovalent cation:H+ antiporter. CPA1 family; [GeneDB:SCO3603] [NCBI-GI:21222019] [NCBI-GeneID:1099039] [UniProt:Q9XAJ9]
SCO3604	356	0.13399	-1.02		SC66T3.15c; hypothetical protein; [GeneDB:SCO3604] [NCBI-GI:21222020] [NCBI-GeneID:1099040] [UniProt:Q9XAJ8]
SCO3622	212	0.04811	-1.30	metabolic process, catalytic activity	SC66T3.33; hypothetical protein; [GeneDB:SCO3622] [NCBI-GI:21222038] [NCBI-GeneID:1099058] [UniProt:Q9XAI0]
SCO3656	269	0.07086	-1.20		SCH10.34c; hypothetical protein; [GeneDB:SCO3656] [NCBI-GI:21222071] [NCBI-GeneID:1099092] [UniProt:Q9X8S3]
SCO3713	191	0.04099	-1.36		SCH35.11; hypothetical protein; [GeneDB:SCO3713] [NCBI-GI:21222126] [NCBI-GeneID:1099149] [UniProt:Q9X8Z5]
SCO3714	264	0.06970	-1.25		SCH35.10; putative transposase; K07496 putative transposase; [GeneDB:SCO3714] [NCBI-GI:21222127] [NCBI-GeneID:1099150] [UniProt:Q9X8Z6]
SCO3800	229	0.05332	-1.21	metabolic process, catalytic activity, nucleotide binding	SCAC2.08. SCGD3.01; putative acyl-CoA dehydrogenase [EC:1.3.99.-]; K00257; [GeneDB:SCO3800] [NCBI-GI:32141216] [NCBI-GeneID:1099236] [UniProt:Q8CJV6]
SCO3816	360	0.13917	-0.99	metabolic process, DNA binding	bkdB1. SCGD3.17c; putative branched-chain alpha keto acid dehydrogenase E1 beta subunit [EC:1.2.4.1]; K00162 pyruvate dehydrogenase E1 component. beta subunit; [GeneDB:SCO3816] [NCBI-GI:21222226] [NCBI-GeneID:1099252] [UniProt:Q9XA61]
SCO3817	208	0.04500	-1.33	metabolic process, catalytic activity	bkdA1. SCGD3.18c; putative branched-chain alpha keto acid dehydrogenase E1 alpha subunit [EC:1.2.4.1]; K00161 pyruvate dehydrogenase E1 component. alpha subunit; [GeneDB:SCO3817] [NCBI-GI:21222227] [NCBI-GeneID:1099253] [UniProt:Q9XA60]
SCO3835	252	0.06278	-1.20	metabolic process, catalytic activity	SCH69.05; dehydrogenase; [GeneDB:SCO3835] [NCBI-GI:21222244] [NCBI-GeneID:1099271] [UniProt:Q9XA29]
SCO3933	102	0.01333	-1.78	metabolic process, transport	SCQ11.16; regulatory protein; [GeneDB:SCO3933] [NCBI-GI:21222339] [NCBI-GeneID:1099369] [UniProt:Q9X9T5]
SCO3982	131	0.02145	-2.19		SCBAC25E3.19; hypothetical protein; [GeneDB:SCO3982] [NCBI-GI:21222386] [NCBI-GeneID:1099418] [UniProt:Q93J25]

SCO3983	189	0.04074	-2.10		SCBAC25E3.20; hypothetical protein; [GeneDB:SCO3983] [NCBI-GI:21222387] [NCBI-GeneID:1099419] [UniProt:Q93J24]
SCO3986	163	0.03055	-2.17	metabolic process, regulation of biological process, response to stimulus, DNA Binding	SCBAC25E3.23; GntR-family transcriptional regulator; [GeneDB:SCO3986] [NCBI-GI:21222390] [NCBI-GeneID:1099422] [UniProt:Q93J21]
SCO3987	184	0.03864	-1.97		SCBAC25E3.24; hypothetical protein; [GeneDB:SCO3987] [NCBI-GI:21222391] [NCBI-GeneID:1099423] [UniProt:Q93J20]
SCO3988	207	0.04483	-1.63		SCBAC25E3.25; hypothetical protein; [GeneDB:SCO3988] [NCBI-GI:21222392] [NCBI-GeneID:1099424] [UniProt:Q93J19]
SCO4053	290	0.08297	-1.20	metabolic process, regulation of biological process, response to stimulus, DNA Binding	2SCD60.19; putative transport integral membrane protein; K07156; K07245 putative copper resistance protein D; [GeneDB:SCO4053] [NCBI-GI:21222456] [NCBI-GeneID:1099489] [UniProt:Q9AK64]
SCO4089	171	0.03228	-1.39	metabolic process, catalytic activity	SCD25.25c. vdh; valine dehydrogenase [EC:1.4.1.-]; K00271; [GeneDB:SCO4089] [NCBI-GI:21222491] [NCBI-GeneID:1099526] [UniProt:Q06539]
SCO4122	343	0.12020	-1.03	metabolic process, regulation of biological process, response to stimulus, DNA Binding stimulus response	SCD72A.08; MarR-family transcriptional regulator; [GeneDB:SCO4122] [NCBI-GI:21222521] [NCBI-GeneID:1099559] [UniProt:Q9KYE8]
SCO4187	111	0.01658	-1.62		2SCD46.01c. SCD66.24c; hypothetical protein; [GeneDB:SCO4187] [NCBI-GI:32141229] [NCBI-GeneID:1099627] [UniProt:Q8CJU4]
SCO4189	117	0.01615	-1.87		2SCD46.03c; hypothetical protein; [GeneDB:SCO4189] [NCBI-GI:21222585] [NCBI-GeneID:1099629] [UniProt:Q7AKJ8]
SCO4301	344	0.12320	-0.99	metabolic process, regulation of biological process, response to stimulus, DNA Binding stimulus response	SCD95A.34c; DNA-binding protein; [GeneDB:SCO4301] [NCBI-GI:21222694] [NCBI-GeneID:1099741] [UniProt:Q9KXU0]
SCO4412	267	0.07019	-1.20		SC6F11.10; regulatory protein; [GeneDB:SCO4412] [NCBI-GI:21222801] [NCBI-GeneID:1099852] [UniProt:Q9F376]
SCO4458	221	0.05109	-1.26		SCD6.36c. SCD65.01c; lipoprotein; [GeneDB:SCO4458] [NCBI-GI:32141237] [NCBI-

					GeneID:1099898] [UniProt:Q8CJT7]
SCO4561	318	0.10635	-1.11		SCD16A.22; NLP/P60 family protein; [GeneDB:SCO4561] [NCBI-GI:21222944] [NCBI-GeneID:1100001] [UniProt:Q9XAQ3]
SCO4562	225	0.05320	-1.29	metabolic process, catalytic activity	nuoA. SCD16A.21c; NADH dehydrogenase I chain A [EC:1.6.5.3]; K00330 NADH dehydrogenase I chain A; [GeneDB:SCO4562] [NCBI-GI:21222945] [NCBI-GeneID:1100002] [UniProt:Q9XAQ4]
SCO4563	167	0.03192	-1.48	metabolic process, catalytic activity, metal ion binding	nuoB. SCD16A.20c; NADH dehydrogenase I chain B [EC:1.6.5.3]; K00331 NADH dehydrogenase I chain B; [GeneDB:SCO4563] [NCBI-GI:21222946] [NCBI-GeneID:1100003] [UniProt:Q9XAQ5]
SCO4564	145	0.02586	-1.60	catalytic activity, metal ion binding	nuoC. SCD16A.19c; NADH dehydrogenase I chain C [EC:1.6.5.3]; K00332 NADH dehydrogenase I chain C; [GeneDB:SCO4564] [NCBI-GI:21222947] [NCBI-GeneID:1100004] [UniProt:Q9XAQ6]
SCO4565	101	0.01257	-1.89	catalytic activity, metal ion binding	nuoD. SCD16A.18c; NADH dehydrogenase I chain D [EC:1.6.5.3]; K00333 NADH dehydrogenase I chain D; [GeneDB:SCO4565] [NCBI-GI:21222948] [NCBI-GeneID:1100005] [UniProt:Q9XAQ7]
SCO4566	125	0.01888	-1.77	metabolic process, catalytic activity, nucleotide binding	nuoE. SCD16A.17c; NADH dehydrogenase I chain E [EC:1.6.5.3]; K00334 NADH dehydrogenase I chain E; [GeneDB:SCO4566] [NCBI-GI:21222949] [NCBI-GeneID:1100006] [UniProt:Q9XAQ8]
SCO4567	110	0.01673	-1.81	metabolic process, catalytic activity, metal ion binding	nuoF. SCD16A.16c; NADH dehydrogenase I chain F [EC:1.6.5.3]; K00335 NADH dehydrogenase I chain F; [GeneDB:SCO4567] [NCBI-GI:21222950] [NCBI-GeneID:1100007]
SCO4568	176	0.03682	-1.46	metabolic process, catalytic activity, metal ion binding	nuoG. SCD16A.15c; NADH dehydrogenase I chain G [EC:1.6.5.3]; K00336 NADH dehydrogenase I chain G; [GeneDB:SCO4568] [NCBI-GI:21222951] [NCBI-GeneID:1100008]
SCO4569	112	0.01643	-1.82	metabolic process, catalytic activity	nuoH. SCD16A.14c; NADH dehydrogenase I chain H [EC:1.6.5.3]; K00337 NADH dehydrogenase I chain H; [GeneDB:SCO4569] [NCBI-GI:21222952] [NCBI-GeneID:1100009] [UniProt:Q9XAR1]
SCO4570	94	0.01000	-2.00	metabolic process, catalytic activity, metal	nuoI. SCD16A.13c; NADH dehydrogenase I chain I [EC:1.6.5.3]; K00338 NADH dehydrogenase I chain I; [GeneDB:SCO4570] [NCBI-GI:21222953] [NCBI-

				ion binding	GeneID:1100010] [UniProt:Q9XAR2]
SCO4571	90	0.00933	-1.98	metabolic process, catalytic activity	nuoJ. SCD16A.12c; NADH dehydrogenase I chain J [EC:1.6.5.3]; K00339 NADH dehydrogenase I chain J; [GeneDB:SCO4571] [NCBI-GI:21222954] [NCBI-GeneID:1100011] [UniProt:Q9XAR3]
SCO4572	77	0.00870	-2.04	catalytic activity,metal ion binding	nuoK. SCD16A.11c; NADH dehydrogenase I chain K [EC:1.6.5.3]; K00340 NADH dehydrogenase I chain K; [GeneDB:SCO4572] [NCBI-GI:21222955] [NCBI-GeneID:1100012] [UniProt:Q9XAR4]
SCO4573	179	0.03782	-1.46	metabolic process, catalytic activity	nuoL. SCD16A.10c; NADH dehydrogenase I chain L [EC:1.6.5.3]; K00341 NADH dehydrogenase I chain L; [GeneDB:SCO4573] [NCBI-GI:21222956] [NCBI-GeneID:1100013]
SCO4574	58	0.00362	-2.21	metabolic process, catalytic activity	nuoM. SCD16A.09c; NADH dehydrogenase I chain M [EC:1.6.5.3]; K00342 NADH dehydrogenase I chain M; [GeneDB:SCO4574] [NCBI-GI:21222957] [NCBI-GeneID:1100014] [UniProt:Q9XAR6]
SCO4575	170	0.03212	-1.47	catalytic activity,metal ion binding	nuoN. SCD16A.08c; NADH dehydrogenase I chain N [EC:1.6.5.3]; K00343 NADH dehydrogenase I chain N; [GeneDB:SCO4575] [NCBI-GI:21222958] [NCBI-GeneID:1100015] [UniProt:Q9XAR7]
SCO4581	282	0.07965	-1.25	metabolic process, catalytic activity	SCD16A.02c; hypothetical protein; K07024; [GeneDB:SCO4581] [NCBI-GI:21222964] [NCBI-GeneID:1100021] [UniProt:Q9XAS3]
SCO4675	276	0.07743	-1.17		SCD40A.21c; hypothetical protein; [GeneDB:SCO4675] [NCBI-GI:21223056] [NCBI-GeneID:1100116] [UniProt:Q9L0J0]
SCO4676	222	0.05108	-1.24		SCD31.01c. SCD40A.22c; hypothetical protein; [GeneDB:SCO4676] [NCBI-GI:32141244] [NCBI-GeneID:1100117] [UniProt:Q8CJT0]
SCO4677	116	0.01629	-1.66		SCD31.02c; regulatory protein; [GeneDB:SCO4677] [NCBI-GI:21223057] [NCBI-GeneID:1100118] [UniProt:Q9L0G5]
SCO4789	219	0.05114	-1.32		SCD63.21; integral membrane protein; [GeneDB:SCO4789] [NCBI-GI:21223168] [NCBI-GeneID:1100230] [UniProt:Q9L0G8]
SCO4790	232	0.05504	-1.24		SCD63.22c. SCD63A.01c; integral membrane protein; [GeneDB:SCO4790] [NCBI-GI:32141246] [NCBI-GeneID:1100231] [UniProt:Q8CJS9]
SCO4793	124	0.01863	-1.67		SCD63A.04c; NPL/P60 family secreted protein; [GeneDB:SCO4793] [NCBI-GI:21223171] [NCBI-GeneID:1100234] [UniProt:Q9KY71]
SCO4799	196	0.04240	-1.37	metabolic process,	SCD63A.10c; secreted lipase; [GeneDB:SCO4799] [NCBI-GI:21223177] [NCBI-

				catalytic activity	GeneID:1100240] [UniProt:Q9KY65]
SCO4800	104	0.01317	-1.73	metabolic process, catalytic activity, metal ion binding	icmB. SCD63A.11; isobutiryl CoA mutase. small subunit [EC:5.4.99.2]; K01849 methylmalonyl-CoA mutase.C-terminal domain; [GeneDB:SCO4800] [NCBI-GI:21223178] [NCBI-GeneID:1100241] [UniProt:Q9KY64]
SCO4894	270	0.07085	-1.13		2SCK8.20c; hypothetical protein 2SCK820c; [GeneDB:SCO4894] [NCBI-GI:21223268] [NCBI-GeneID:1100335] [UniProt:Q9AK32]
SCO4920	251	0.06295	-1.26	metabolic process, regulation of biological process, DNA Binding	SCK13.12; DeoR-family transcriptional regulator; [GeneDB:SCO4920] [NCBI-GI:21223294] [NCBI-GeneID:1100361] [UniProt:Q9AD80]
SCO4930	130	0.02008	-1.63	metabolic process, catalytic activity	SCK13.22; putative enoyl-CoA hydratase [EC:4.2.1.17]; K01692 enoyl-CoA hydratase; [GeneDB:SCO4930] [NCBI-GI:21223304] [NCBI-GeneID:1100371] [UniProt:Q9EWW0]
SCO4974	310	0.10126	-1.05	metabolic process, catalytic activity, metal ion binding	2SCK31.34; deaminase; [GeneDB:SCO4974] [NCBI-GI:21223347] [NCBI-GeneID:1100415] [UniProt:Q9ADJ9]
SCO4979	24	0.00125	-2.61	metabolic process, catalytic activity	2SCK36.02; phosphoenolpyruvate carboxykinase (GTP) [EC:4.1.1.32]; K01596 phosphoenolpyruvate carboxykinase (GTP); [GeneDB:SCO4979] [NCBI-GI:21223352] [NCBI-GeneID:1100420] [UniProt:Q93JL5]
SCO5028	122	0.01820	-1.65		SCK7.01; putative ATP-binding protein; K07175 PhoH-like ATPase; [GeneDB:SCO5028] [NCBI-GI:21223401] [NCBI-GeneID:1100469] [UniProt:Q9FBP8]
SCO5047	285	0.08105	-1.17		SCK7.20c; fructose-1.6-bisphosphatase II / sedoheptulose-1.7-bisphosphatase [EC:3.1.3.11]; K02446 fructose-1.6-bisphosphatase II; [GeneDB:SCO5047] [NCBI-GI:21223420] [NCBI-GeneID:1100488] [UniProt:Q9FBN2]
SCO5054	204	0.04422	-1.30		SCK7.27; hypothetical protein; [GeneDB:SCO5054] [NCBI-GI:21223427] [NCBI-GeneID:1100495] [UniProt:Q9FBM5]
SCO5071	54	0.00333	-2.44		SCBAC20F6.14c; hydroxylacyl-CoA dehydrogenase; [GeneDB:SCO5071] [NCBI-GI:21223443] [NCBI-GeneID:1100512] [UniProt:Q53925]
SCO5072	323	0.10768	-1.13	metabolic process, catalytic activity, nucleotide binding	SCBAC20F6.15; hydroxylacyl-CoA dehydrogenase [EC:1.1.1.-]; K05556 hydroxylacyl-CoA dehydrogenase; [GeneDB:SCO5072] [NCBI-GI:21223444] [NCBI-GeneID:1100513] [UniProt:Q53926]
SCO5073	255	0.06329	-1.21	metabolic process, catalytic activity, metal	SCBAC20F6.16; putative oxidoreductase [EC:1.6.5.5]; K00344 NADPH2:quinone reductase; [GeneDB:SCO5073] [NCBI-GI:21223445] [NCBI-GeneID:1100514]

				ion binding	[UniProt:Q53927]
SCO5074	82	0.00902	-1.88		SCBAC20F6.17; dehydratase; [GeneDB:SCO5074] [NCBI-GI:21223446] [NCBI-GeneID:1100515] [UniProt:Q9ADD7]
SCO5079	113	0.01628	-1.62	metabolic process, catalytic activity	actVA4. SCBAC28G1.05; hypothetical protein; [GeneDB:SCO5079] [NCBI-GI:21223450] [NCBI-GeneID:1100520] [UniProt:Q53906]
SCO5080	201	0.04348	-1.29	metabolic process, catalytic activity, nucleotide binding	actVA5. SCBAC28G1.06; hydrolase; [GeneDB:SCO5080] [NCBI-GI:21223451] [NCBI-GeneID:1100521] [UniProt:Q53907]
SCO5081	180	0.03811	-1.35		actVA6. SCBAC28G1.07; hypothetical protein; [GeneDB:SCO5081] [NCBI-GI:21223452] [NCBI-GeneID:1100522] [UniProt:Q53908]
SCO5086	123	0.01837	-1.62	metabolic process, catalytic activity	actIII. SCBAC28G1.12c; ketoacyl reductase [EC:1.3.1.-]; K00224; [GeneDB:SCO5086] [NCBI-GI:21223457] [NCBI-GeneID:1100527] [UniProt:P16544]
SCO5089	70	0.00714	-2.06	metabolic process	actIORF3. SCBAC28G1.15; actinorhodin polyketide synthase acyl carrier protein; K05553 3-oxoacyl-ACP synthase acyl carrier protein; [GeneDB:SCO5089] [NCBI-GI:21223460] [NCBI-GeneID:1100530] [UniProt:Q02054]
SCO5112	173	0.03474	-1.57	transporter activity	bldKA. SCBAC31E11.08; BldKA. ABC transport system integral membrane protein; [GeneDB:SCO5112] [NCBI-GI:21223483] [NCBI-GeneID:1100553] [UniProt:Q93IU3]
SCO5113	205	0.04473	-1.43	transporter activity	bldKB. SCBAC31E11.09; BldKB. putative ABC transport system lipoprotein; K02035 peptide/nickel transport system substrate-binding protein; [GeneDB:SCO5113] [NCBI-GI:21223484] [NCBI-GeneID:1100554] [UniProt:Q93IU2]
SCO5114	193	0.04197	-1.51	transporter activity	bldKC. SCBAC31E11.10; BldKC. putative ABC transport system integral membrane protein; K02033 peptide/nickel transport system permease protein; [GeneDB:SCO5114] [NCBI-GI:21223485] [NCBI-GeneID:1100555] [UniProt:Q93IU1]
SCO5115	268	0.07007	-1.32	transporter activity	bldKD. SCBAC31E11.11; BldKD. putative ABC transporter intracellular ATPase subunit; K02031 peptide/nickel transport system ATP-binding protein; [GeneDB:SCO5115] [NCBI-GI:21223486] [NCBI-GeneID:1100556] [UniProt:Q93IU0]
SCO5116	257	0.06681	-1.34	metabolic process, transport	bldKE. SC9E12.01. SCBAC31E11.12; putative peptide transport system ATP-binding subunit; K02032 peptide/nickel transport system ATP-binding protein; [GeneDB:SCO5116] [NCBI-GI:32141254] [NCBI-GeneID:1100557] [UniProt:Q8CJS2]

SCO5117	224	0.05223	-1.23	transport	SC9E12.02; putative peptide transport system secreted peptide-binding protein; K02035 peptide/nickel transport system substrate-binding protein; [GeneDB:SCO5117] [NCBI-GI:21223487] [NCBI-GeneID:1100558] [UniProt:Q9F353]
SCO5125	335	0.11719	-0.99	DNA Binding	SC9E12.10c; hypothetical protein; [GeneDB:SCO5125] [NCBI-GI:21223495] [NCBI-GeneID:1100566] [UniProt:Q9F345]
SCO5174	162	0.02963	-1.48	metabolic process, catalytic activity	SCP8.37; transferase; [GeneDB:SCO5174] [NCBI-GI:21223543] [NCBI-GeneID:1100615] [UniProt:Q9FBI5]
SCO5175	151	0.02629	-1.51		SCP8.38; integral membrane protein; [GeneDB:SCO5175] [NCBI-GI:21223544] [NCBI-GeneID:1100616] [UniProt:Q9FBI4]
SCO5176	155	0.02858	-1.50	metabolic process, catalytic activity	SCP8.39; reductase; [GeneDB:SCO5176] [NCBI-GI:21223545] [NCBI-GeneID:1100617] [UniProt:Q9FBI3]
SCO5177	159	0.02937	-1.48		2SC3B6.01; hypothetical protein 2SC3B601; [GeneDB:SCO5177] [NCBI-GI:21223546] [NCBI-GeneID:1100618] [UniProt:Q9FCL1]
SCO5254	47	0.00277	-2.34	metabolic process, catalytic activity, metal ion binding	2SC7G11.16c. sodN; superoxide dismutase [EC:1.15.1.1]; K00518 superoxide dismutase; [GeneDB:SCO5254] [NCBI-GI:21223621] [NCBI-GeneID:1100695] [UniProt:P80735]
SCO5311	46	0.00261	-2.35		SC6G9.22; secreted protein; [GeneDB:SCO5311] [NCBI-GI:21223674] [NCBI-GeneID:1100751] [UniProt:Q9XAG0]
SCO5350	260	0.06827	-1.23		SCBAC5H2.19; hypothetical protein; [GeneDB:SCO5350] [NCBI-GI:21223711] [NCBI-GeneID:1100790] [UniProt:Q9ADB8]
SCO5385	351	0.12544	-0.99	metabolic process, catalytic activity, nucleotide binding	2SC6G5.29; putative 3-hydroxybutyryl-coA dehydrogenase [EC:1.1.1.157]; K00074 3-hydroxybutyryl-CoA dehydrogenase; [GeneDB:SCO5385] [NCBI-GI:21223745] [NCBI-GeneID:1100825] [UniProt:Q9K4C6]
SCO5390	248	0.06246	-1.28	metabolic process, catalytic activity	2SC6G5.34c; alkanal monooxygenase (luciferase); [GeneDB:SCO5390] [NCBI-GI:21223750] [NCBI-GeneID:1100830] [UniProt:Q9K4C1]
SCO5398	309	0.09835	-1.07		SC8F4.02c; hypothetical protein; [GeneDB:SCO5398] [NCBI-GI:21223758] [NCBI-GeneID:1100838] [UniProt:Q9L2C2]
SCO5399	61	0.00393	-2.02	metabolic process, catalytic activity	SC8F4.03; probable acetoacetyl-coA thiolase [EC:2.3.1.9]; K00626 acetyl-CoA C-acetyltransferase; [GeneDB:SCO5399] [NCBI-GI:21223759] [NCBI-GeneID:1100839] [UniProt:Q9L2C1]
SCO5400	118	0.01653	-1.67	cell organization and biogenesis, metabolic	SC8F4.04; putative transport system kinase [EC:2.7.-.-]; K07588 LAO/AO transport system kinase; [GeneDB:SCO5400] [NCBI-GI:21223760] [NCBI-GeneID:1100840] [UniProt:Q9L2C0]

				process, transport	
SCO5415	33	0.00152	-2.47	metabolic process, DNA binding	icmA. SC8F4.19; isobutyryl-CoA mutase A [EC:5.4.99.2]; K01848 methylmalonyl-CoA mutase. N-terminal domain; [GeneDB:SCO5415] [NCBI-GI:21223775] [NCBI-GeneID:1100855] [UniProt:Q9X949]
SCO5420	324	0.10769	-1.03		SC8F4.24; cholesterol esterase; [GeneDB:SCO5420] [NCBI-GI:21223780] [NCBI-GeneID:1100860] [UniProt:Q9L2A2]
SCO5421	300	0.09483	-1.11		SC8F4.25; integral membrane protein; [GeneDB:SCO5421] [NCBI-GI:21223781] [NCBI-GeneID:1100861] [UniProt:Q9L2A1]
SCO5442	321	0.10766	-1.12	metabolic process, catalytic activity	SC6A11.18c; trehalose synthase; [GeneDB:SCO5442] [NCBI-GI:21223801] [NCBI-GeneID:1100882] [UniProt:Q9L1K3]
SCO5443	382	0.14990	-0.94	metabolic process, catalytic activity	pep1A; putative alpha-amylase [EC:3.2.1.-]; K01238; [GeneDB:SCO5443] [NCBI-GI:21223802] [NCBI-GeneID:1100883] [UniProt:Q9L1K2]
SCO5444	301	0.09571	-1.18	metabolic process, catalytic activity	glgP. SC3D11.01. SC6A11.20; putative glycogen phosphorylase [EC:2.4.1.1]; K00688 starch phosphorylase; [GeneDB:SCO5444] [NCBI-GI:32141263] [NCBI-GeneID:1100884] [UniProt:Q8CJR4]
SCO5447	109	0.01688	-1.64	metabolic process, catalytic activity, metal ion binding	SC3D11.04c; neutral zinc metalloprotease; [GeneDB:SCO5447] [NCBI-GI:21223805] [NCBI-GeneID:1100887] [UniProt:Q9L1F8]
SCO5459	302	0.09629	-1.08	metabolic process, catalytic activity	SC3D11.16; enoyl-coA hydratase; [GeneDB:SCO5459] [NCBI-GI:21223817] [NCBI-GeneID:1100899] [UniProt:Q9L1E6]
SCO5466	197	0.04284	-1.28	metabolic process, catalytic activity	SC3D11.23c; hydrolase; [GeneDB:SCO5466] [NCBI-GI:21223824] [NCBI-GeneID:1100906] [UniProt:Q9L1D9]
SCO5476	313	0.10447	-1.07	transporter activity	SC2A11.10; oligopeptide transport integral membrane protein; K02034 peptide/nickel transport system permease protein; [GeneDB:SCO5476] [NCBI-GI:21223833] [NCBI-GeneID:1100916] [UniProt:O86571]
SCO5477	284	0.08039	-1.11	transporter activity	SC2A11.11; putative oligopeptide-binding lipoprotein; K02035 peptide/nickel transport system substrate-binding protein; [GeneDB:SCO5477] [NCBI-GI:21223834] [NCBI-GeneID:1100917] [UniProt:O86572]
SCO5478	199	0.04256	-1.30	transporter activity	SC2A11.12; oligopeptide transport system integral membrane protein; K02033 peptide/nickel transport system permease protein; [GeneDB:SCO5478] [NCBI-GI:21223835] [NCBI-GeneID:1100918] [UniProt:O86573]

SCO5479	329	0.11179	-1.06	metabolic process, transport	SC2A11.13; oligopeptide ABC transporter ATP-binding protein; K02031 peptide/nickel transport system ATP-binding protein; [GeneDB:SCO5479] [NCBI-GI:21223836] [NCBI-GeneID:1100919] [UniProt:O86574]
SCO5480	303	0.09604	-1.10	metabolic process, transport	SC2A11.14; oligopeptide ABC transporter ATP-binding protein; K02032 peptide/nickel transport system ATP-binding protein; [GeneDB:SCO5480] [NCBI-GI:21223837] [NCBI-GeneID:1100920] [UniProt:O86575]
SCO5511	322	0.10748	-1.05	transporter activity	SC8D9.23; membrane associated phophodiesterase; [GeneDB:SCO5511] [NCBI-GI:21223867] [NCBI-GeneID:1100951] [UniProt:Q9Z568]
SCO5629	249	0.06249	-1.32	stimulus response	SC6A9.38; ATP/GTP-binding protein; [GeneDB:SCO5629] [NCBI-GI:21223984] [NCBI-GeneID:1101070] [UniProt:O86768]
SCO5644	182	0.03808	-1.38		SC6A9.23c; hypothetical protein; [GeneDB:SCO5644] [NCBI-GI:21223995] [NCBI-GeneID:1101083] [UniProt:O86755]
SCO5658	81	0.00901	-1.84		SC6A9.09c; polyamine-binding lipoprotein; [GeneDB:SCO5658] [NCBI-GI:21224009] [NCBI-GeneID:1101097] [UniProt:O86741]
SCO5667	305	0.09570	-1.10	transporter activity	SC8B7.11c; putative ABC-transporter polyamine-binding lipoprotein; K02055 spermidine/putrescine transport system substrate-binding protein; [GeneDB:SCO5667] [NCBI-GI:21224017] [NCBI-GeneID:1101106] [UniProt:O86832]
SCO5676	13	0.00077	-2.96	metabolic process, catalytic activity	gabT; putative 4-aminobutyrate aminotransferase [EC:2.6.1.19]; K00823 4-aminobutyrate aminotransferase; [GeneDB:SCO5676] [NCBI-GI:21224026] [NCBI-GeneID:1101115] [UniProt:O86823]
SCO5679	63	0.00397	-1.98	metabolic process, catalytic activity	SC5H4.03; putative aldehyde dehydrogenase [EC:1.2.1.3]; K00128 aldehyde dehydrogenase (NAD+); [GeneDB:SCO5679] [NCBI-GI:21224028] [NCBI-GeneID:1101118] [UniProt:Q9KYT6]
SCO5784	304	0.09586	-1.07	cell communication, metabolic process, regulation of biological process, response to stimulus, DNA binding	SC4H2.05; two-component sensor; [GeneDB:SCO5784] [NCBI-GI:21224129] [NCBI-GeneID:1101226] [UniProt:O69960]
SCO5785	237	0.05827	-1.26	cell communication, metabolic process,	SC4H2.06; two-component regulator; [GeneDB:SCO5785] [NCBI-GI:21224130] [NCBI-GeneID:1101227] [UniProt:O69961]

				regulation of biological process, response to stimulus, DNA binding stimulus response	
SCO5811	347	0.12303	-1.01	metabolic process, catalytic activity, DNA binding	SC4H2.32; transcriptional regulator; [GeneDB:SCO5811] [NCBI-GI:32141274] [NCBI-GeneID:1101253] [UniProt:Q8CJQ4]
SCO5819	247	0.06267	-1.23	cell communication, metabolic process, regulation of biological process, response to stimulus, DNA binding	sporulation transcription factor. WhiH; [GeneDB:SCO5819] [NCBI-GI:21224163] [NCBI-GeneID:1101261] [UniProt:O50536]
SCO5879	340	0.11809	-1.05	metabolic process, catalytic activity, nucleotide binding	redW; acyl-CoA dehydrogenase RedW; [GeneDB:SCO5879] [NCBI-GI:21224222] [NCBI-GeneID:1101321] [UniProt:O54143]
SCO5880	152	0.02638	-1.50		redY; RedY protein; [GeneDB:SCO5880] [NCBI-GI:21224223] [NCBI-GeneID:1101322] [UniProt:O54144]
SCO5884	278	0.07878	-1.20		SC3F7.04c; hypothetical protein; [GeneDB:SCO5884] [NCBI-GI:21224226] [NCBI-GeneID:1101326] [UniProt:O54147]
SCO5885	21	0.00095	-2.90		SC3F7.05c; hypothetical protein; [GeneDB:SCO5885] [NCBI-GI:21224227] [NCBI-GeneID:1101327] [UniProt:O54148]
SCO5886	272	0.07147	-1.24	metabolic process, catalytic activity	redR. SC3F7.06c; 3-oxoacyl-[acyl-carrier-protein] synthase II [EC:2.3.1.179]; K09458 3-oxoacyl-[acyl-carrier-protein] synthase II; [GeneDB:SCO5886] [NCBI-GI:21224228] [NCBI-GeneID:1101328] [UniProt:O54149]
SCO5887	246	0.06276	-1.31		redQ. SC3F7.07c; acyl carrier protein; K02078 acyl carrier protein; [GeneDB:SCO5887] [NCBI-GI:21224229] [NCBI-GeneID:1101329] [UniProt:O54150]
SCO5888	345	0.12290	-1.10	metabolic process, catalytic activity	redP. SC3F7.08; 3-oxoacyl-[acyl-carrier-protein] synthase [EC:2.3.1.180]; K00648 3-oxoacyl-[acyl-carrier-protein] synthase III; [GeneDB:SCO5888] [NCBI-GI:21224230] [NCBI-GeneID:1101330] [UniProt:O54151]
SCO5889	27	0.00148	-2.75		redO. SC3F7.09; hypothetical protein; [GeneDB:SCO5889] [NCBI-GI:21224231] [NCBI-GeneID:1101331] [UniProt:O54152]
SCO5890	187	0.03973	-1.50	metabolic process, catalytic activity	redN. SC3F7.10; 8-amino-7-oxononanoate synthase; [GeneDB:SCO5890] [NCBI-GI:21224232] [NCBI-GeneID:1101332] [UniProt:O54153]
SCO5891	233	0.05494	-1.31	metabolic process,	redM. St3F7.11; peptide synthase; [GeneDB:SCO5891] [NCBI-GI:21224233] [NCBI-

				catalytic activity, nucleotide binding	GeneID:1101333] [UniProt:O54154]
SCO5893	168	0.03185	-1.53	metabolic process, catalytic activity	redK. SC3F7.13; oxidoreductase; [GeneDB:SCO5893] [NCBI-GI:21224235] [NCBI-GeneID:1101335] [UniProt:O54156]
SCO5894	277	0.07791	-1.12	metabolic process, catalytic activity	redJ. SC3F7.14; thioesterase; [GeneDB:SCO5894] [NCBI-GI:21224236] [NCBI-GeneID:1101336] [UniProt:O54157]
SCO5895	166	0.03084	-1.50	metabolic process, catalytic activity	redI. SC3F7.15; methyltransferase; [GeneDB:SCO5895] [NCBI-GI:21224237] [NCBI-GeneID:1101337] [UniProt:O54158]
SCO5896	198	0.04263	-1.43	metabolic process, catalytic activity, nucleotide binding	redH. SC10A5.01. SC3F7.16; phosphoenolpyruvate-utilizing enzyme [EC:2.7.9.2]; K01007 pyruvate.water dikinase; [GeneDB:SCO5896] [NCBI-GI:32141277] [NCBI-GeneID:1101338] [UniProt:Q8CJQ2]
SCO5897	87	0.00931	-1.97	metabolic process, catalytic activity	redG. SC10A5.02; oxidase; [GeneDB:SCO5897] [NCBI-GI:21224238] [NCBI-GeneID:1101339] [UniProt:O54095]
SCO5898	283	0.07943	-1.17	metabolic process, catalytic activity	redF. SC10A5.03; probable membrane protein; [GeneDB:SCO5898] [NCBI-GI:21224239] [NCBI-GeneID:1101340] [UniProt:O54096]
SCO5932	376	0.14896	-1.04	metabolic process, catalytic activity	abfB; arabinofuranosidase [EC:3.2.1.55]; K01209 alpha-N-arabinofuranosidase; [GeneDB:SCO5932] [NCBI-GI:21224270] [NCBI-GeneID:1101374] [UniProt:O54161]
SCO5965	234	0.05521	-1.53		SC7H1.35; integral membrane protein; [GeneDB:SCO5965] [NCBI-GI:21224303] [NCBI-GeneID:1101407] [UniProt:O54194]
SCO5993	146	0.02589	-1.48		StBAC16H6.28; hypothetical protein; [GeneDB:SCO5993] [NCBI-GI:21224330] [NCBI-GeneID:1101435] [UniProt:Q93JD4]
SCO6026	213	0.04826	-1.24	metabolic process, catalytic activity	SC1C3.14c; fatty acid oxidation complex alpha-subunit; [GeneDB:SCO6026] [NCBI-GI:21224358] [NCBI-GeneID:1101467] [UniProt:O69856]
SCO6027	211	0.04725	-1.23	metabolic process, catalytic activity	SC1C3.15c; acetyl-coa acetyltransferase (thiolase); [GeneDB:SCO6027] [NCBI-GI:21224359] [NCBI-GeneID:1101468] [UniProt:O69857]
SCO6155	334	0.11713	-1.06	metabolic process, catalytic activity, metal ion binding	SC1A9.19; pyruvate dehydrogenase [EC:1.2.2.2]; K00156 pyruvate dehydrogenase (cytochrome); [GeneDB:SCO6155] [NCBI-GI:21224481] [NCBI-GeneID:1101596] [UniProt:Q9ZBT3]
SCO6160	6	0.00000	-3.46	transporter activity	SC1A9.24c; putative SecDF protein-export membrane protein; K03072 preprotein translocase SecD subunit; K03074 preprotein translocase SecF subunit; [GeneDB:SCO6160] [NCBI-GI:21224486] [NCBI-GeneID:1101601] [UniProt:Q9ZBS8]
SCO6161	16	0.00125	-2.87		SC1A9.25c; secreted protein; [GeneDB:SCO6161] [NCBI-GI:21224487] [NCBI-GeneID:1101602] [UniProt:Q9ZBS7]
SCO6162	36	0.00250	-2.50	cell communication,	SC1A9.26c; two-component system response regulator; [GeneDB:SCO6162] [NCBI-

				metabolic process, regulation of biological process, response to stimulus, DNA binding stimulus response	GI:21224488] [NCBI-GeneID:1101603] [UniProt:Q9ZBS6]
SCO6163	76	0.00789	-2.09	metabolic process, regulation of biological process, response to stimulus, DNA binding	SC1A9.27c; sensor kinase; [GeneDB:SCO6163] [NCBI-GI:21224489] [NCBI-GeneID:1101604] [UniProt:Q9ZBS5]
SCO6164	1	0.00000	-5.26		SC1A9.28c; putative DnaK suppressor protein; K06204 DnaK suppressor protein; [GeneDB:SCO6164] [NCBI-GI:21224490] [NCBI-GeneID:1101605] [UniProt:Q9ZBS4]
SCO6165	11	0.00000	-3.07		SC1A9.29c. SC6C5.01; hypothetical protein; [GeneDB:SCO6165] [NCBI-GI:32141289] [NCBI-GeneID:1101606] [UniProt:Q8CJP0]
SCO6166	5	0.00000	-3.64		SC6C5.02; hypothetical protein; [GeneDB:SCO6166] [NCBI-GI:21224491] [NCBI-GeneID:1101607] [UniProt:Q9ZBP3]
SCO6167	30	0.00133	-2.56		SC6C5.03; proline rich protein (membrane protein); [GeneDB:SCO6167] [NCBI-GI:21224492] [NCBI-GeneID:1101608] [UniProt:Q9ZBP2]
SCO6173	368	0.14245	-0.97	transporter activity	SC6C5.09; putative permease SC6C5.09; K06901 putative MFS transporter. AGZA family. xanthine/uracil permease; [GeneDB:SCO6173] [NCBI-GI:21224498] [NCBI-GeneID:1101614] [UniProt:Q9ZBN6]
SCO6205	364	0.14184	-1.00	metabolic process, catalytic activity	SC2G5.26c; putative dehydrogenase [EC:1.1.1.60]; K00042 2-hydroxy-3-oxopropionate reductase; [GeneDB:SCO6205] [NCBI-GI:21224529] [NCBI-GeneID:1101646] [UniProt:Q9Z597]
SCO6206	350	0.12386	-1.05	metabolic process, DNA binding	SC2G5.27c; putative oxidoreductase [EC:5.3.1.22]; K01816 hydroxypyruvate isomerase; [GeneDB:SCO6206] [NCBI-GI:21224530] [NCBI-GeneID:1101647] [UniProt:Q9Z596]
SCO6243	259	0.06776	-1.22	metabolic process, catalytic activity	aceB1. SCAH10.08c; malate synthase [EC:2.3.3.9]; K01638 malate synthase; [GeneDB:SCO6243] [NCBI-GI:21224564] [NCBI-GeneID:1101684] [UniProt:Q9RKU9]
SCO6375	206	0.04481	-1.26		SC4A2.11c; secreted protein; [GeneDB:SCO6375] [NCBI-GI:21224687] [NCBI-GeneID:1101816] [UniProt:O86667]
SCO6393	375	0.14891	-1.04	DNA Binding	SC3C8.12; transposase; [GeneDB:SCO6393] [NCBI-GI:21224705] [NCBI-GeneID:1101834] [UniProt:O69924]
SCO6414	156	0.02904	-1.47	metabolic process,	SC1A6.03; hypothetical protein; [GeneDB:SCO6414] [NCBI-GI:21224721] [NCBI-

				catalytic activity	GeneID:1101853] [UniProt:O69808]
SCO6416	314	0.10427	-1.07	metabolic process, catalytic activity	SC1A6.05; oxidoreductase; [GeneDB:SCO6416] [NCBI-GI:21224723] [NCBI-GeneID:1101855] [UniProt:O69810]
SCO6417	244	0.06045	-1.29	transporter activity	SC1A6.06; putative integral membrane transporter; K03457 nucleobase:cation symporter-1. NCS1 family; [GeneDB:SCO6417] [NCBI-GI:21224724] [NCBI-GeneID:1101856] [UniProt:O69811]
SCO6423	154	0.02799	-1.48	metabolic process, catalytic activity	SC1A6.12c; putative lipoate-protein ligase [EC:2.7.7.63]; K03800 lipoate-protein ligase A; [GeneDB:SCO6423] [NCBI-GI:21224730] [NCBI-GeneID:1101862] [UniProt:O69817]
SCO6424	44	0.00273	-2.44	response to stimulus	SC1A6.13c; two-component system sensor protein; [GeneDB:SCO6424] [NCBI-GI:21224731] [NCBI-GeneID:1101863] [UniProt:O69818]
SCO6469	352	0.12636	-1.01	metabolic process, catalytic activity	SC9C7.05c; putative acyl-CoA dehydrogenase [EC:1.3.99.-]; K00257; [GeneDB:SCO6469] [NCBI-GI:21224773] [NCBI-GeneID:1101908] [UniProt:Q9ZBK5]
SCO6470	333	0.11676	-1.06	metabolic process, catalytic activity	SC9C7.06c; hypothetical protein; [GeneDB:SCO6470] [NCBI-GI:21224774] [NCBI-GeneID:1101909] [UniProt:Q9ZBK4]
SCO6472	74	0.00743	-1.82	metabolic process, catalytic activity, metal ion binding	SC9C7.08c; coenzyme B12-dependent mutase [EC:5.4.99.2]; K01848 methylmalonyl-CoA mutase.N-terminal domain; K01849 methylmalonyl-CoA mutase. C-terminal domain; [GeneDB:SCO6472] [NCBI-GI:21224776] [NCBI-GeneID:1101911] [UniProt:Q9ZBK2]
SCO6473	31	0.00129	-2.44	metabolic process, catalytic activity	SC9C7.09c; crotonyl CoA reductase; [GeneDB:SCO6473] [NCBI-GI:21224777] [NCBI-GeneID:1101912] [UniProt:Q9ZBK1]
SCO6475	358	0.13827	-1.01	metabolic process, catalytic activity, nucleotide binding	SC9C7.11c; oxidoreductase; [GeneDB:SCO6475] [NCBI-GI:21224779] [NCBI-GeneID:1101914] [UniProt:Q9ZBJ9]
SCO6629	136	0.02419	-1.52	DNA binding	SC4G2.03; hypothetical protein; [GeneDB:SCO6629] [NCBI-GI:21224926] [NCBI-GeneID:1102068] [UniProt:O86676]
SCO6641	291	0.08368	-1.16		SC4G2.15c; hypothetical protein; [GeneDB:SCO6641] [NCBI-GI:21224938] [NCBI-GeneID:1102080] [UniProt:O86687]
SCO6647	53	0.00321	-2.22	metabolic process, catalytic activity	SC4G2.21c; integral membrane protein; [GeneDB:SCO6647] [NCBI-GI:21224944] [NCBI-GeneID:1102086] [UniProt:O86693]
SCO6650	2	0.00000	-4.13		SC4G2.24; hypothetical protein; [GeneDB:SCO6650] [NCBI-GI:21224947] [NCBI-GeneID:1102089] [UniProt:O86696]
SCO6651	9	0.00000	-3.14	metabolic process, catalytic activity	SC4G2.25. SC5A7.01; glycosyl transferase; [GeneDB:SCO6651] [NCBI-GI:32141310] [NCBI-GeneID:1102090] [UniProt:Q8CJM1]

SCO6652	17	0.00118	-3.03		SC5A7.02; hypothetical protein; [GeneDB:SCO6652] [NCBI-GI:21224948] [NCBI-GeneID:1102091] [UniProt:O88008]
SCO6653	73	0.00712	-2.09		SC5A7.03; hypothetical protein; [GeneDB:SCO6653] [NCBI-GI:21224949] [NCBI-GeneID:1102092] [UniProt:O88009]
SCO6655	10	0.00000	-3.14	metabolic process, catalytic activity, metal ion binding	ribA2. SC5A7.05; GTP cyclohydrolase II [EC:3.5.4.25]; K01497 GTP cyclohydrolase II; [GeneDB:SCO6655] [NCBI-GI:21224951] [NCBI-GeneID:1102094] [UniProt:O88011]
SCO6749	138	0.02435	-1.58		SC5F2A.32; hypothetical protein; [GeneDB:SCO6749] [NCBI-GI:21225043] [NCBI-GeneID:1102188] [UniProt:Q9X7Q5]
SCO6794	286	0.08213	-1.22		cvnA7. SC1A2.03; membrane protein; [GeneDB:SCO6794] [NCBI-GI:21225087] [NCBI-GeneID:1102233] [UniProt:Q9L238]
SCO6795	160	0.02931	-1.56		cvnB7. SC1A2.04; hypothetical protein; [GeneDB:SCO6795] [NCBI-GI:21225088] [NCBI-GeneID:1102234] [UniProt:Q9L237]
SCO6796	186	0.03919	-1.45		cvnC7. SC1A2.05; hypothetical protein; [GeneDB:SCO6796] [NCBI-GI:21225089] [NCBI-GeneID:1102235] [UniProt:Q9L236]
SCO6797	115	0.01626	-1.74		cvnD7. SC1A2.06; ATP/GTP binding protein; [GeneDB:SCO6797] [NCBI-GI:21225090] [NCBI-GeneID:1102236] [UniProt:Q9L235]
SCO6798	190	0.04100	-1.47		SC1A2.07; hypothetical protein; [GeneDB:SCO6798] [NCBI-GI:21225091] [NCBI-GeneID:1102237] [UniProt:Q9L234]
SCO6885	275	0.07462	-1.33	metabolic process, regulation of biological process, DNA binding, catalytic activity	SC7F9.37; putative DNA methylase [EC:2.1.1.72]; K07319 putative adenine-specific DNA-methyltransferase; [GeneDB:SCO6885] [NCBI-GI:21225176] [NCBI-GeneID:1102324] [UniProt:Q9L1N0]
SCO6906	274	0.07471	-1.16		SC1B2.12c; hypothetical protein; [GeneDB:SCO6906] [NCBI-GI:21225196] [NCBI-GeneID:1102345] [UniProt:Q9KYC4]
SCO6919	293	0.08539	-1.12		SC1B2.25c; hypothetical protein; [GeneDB:SCO6919] [NCBI-GI:21225207] [NCBI-GeneID:1102357] [UniProt:Q9KYB4]
SCO6941	209	0.04483	-1.39		cvnC8. SC1G8.13c; hypothetical protein; [GeneDB:SCO6941] [NCBI-GI:21225228] [NCBI-GeneID:1102379] [UniProt:Q9KYK0]
SCO6982	317	0.10420	-1.09		SC8F11.08; IolE protein; K03335 myo-inositol catabolism protein IolE; [GeneDB:SCO6982] [NCBI-GI:21225268] [NCBI-GeneID:1102420] [UniProt:Q9KZH0]
SCO6992	133	0.02353	-1.60	metabolic process, catalytic activity, metal ion binding	absR1. SC8F11.18c; regulatory protein; [GeneDB:SCO6992] [NCBI-GI:21225278] [NCBI-GeneID:1102430] [UniProt:Q9RGD7]
SCO7040	287	0.08345	-1.34	metabolic process,	gap2. SC4G1.06c; glyceraldehyde-3-phosphate dehydrogenase;

				catalytic activity, nucleotide binding	[GeneDB:SCO7040] [NCBI-GI:21225323] [NCBI-GeneID:1102478] [UniProt:Q9FC43]
SCO7079	370	0.14432	-0.99		SC3A4.05; pseudogene; [GeneDB:SCO7079] [NCBI-GeneID:1102517]
SCO7095	174	0.03500	-1.46	metabolic process, catalytic activity	SC3A4.21c; hydrolase; [GeneDB:SCO7095] [NCBI-GI:21225377] [NCBI-GeneID:1102533] [UniProt:Q9KZ37]
SCO7130	273	0.07341	-1.22		SC4B10.31; hypothetical protein; [GeneDB:SCO7130] [NCBI-GI:21225412] [NCBI-GeneID:1102568] [UniProt:Q9FC56]
SCO7131	288	0.08347	-1.08	metabolic process, catalytic activity, metal ion binding	SC4B10.32; lipase; [GeneDB:SCO7131] [NCBI-GI:21225413] [NCBI-GeneID:1102569] [UniProt:Q9FC55]
SCO7189	338	0.11737	-1.17		SC8A11.17c; hypothetical protein; [GeneDB:SCO7189] [NCBI-GI:21225468] [NCBI-GeneID:1102627] [UniProt:Q9FBZ3]
SCO7190	86	0.00942	-1.81	metabolic process, catalytic activity	SC8A11.18; hypothetical protein; [GeneDB:SCO7190] [NCBI-GI:21225469] [NCBI-GeneID:1102628] [UniProt:Q9FBZ2]
SCO7197	132	0.02182	-1.61	transporter activity	SC8A11.25c; amino acid transport integral membrane protein; [GeneDB:SCO7197] [NCBI-GI:21225476] [NCBI-GeneID:1102635] [UniProt:Q9FBY5]
SCO7208	341	0.11792	-1.01		SC2H12.07c; hypothetical protein; [GeneDB:SCO7208] [NCBI-GI:21225485] [NCBI-GeneID:1102646] [UniProt:Q9K477]
SCO7226	202	0.04337	-1.26		SC2H12.25; integral membrane protein; [GeneDB:SCO7226] [NCBI-GI:21225503] [NCBI-GeneID:1102664] [UniProt:Q9K459]
SCO7253	298	0.09010	-1.14	cell communication, metabolic process, stimulus response	SC5H1.39; hypothetical protein; [GeneDB:SCO7253] [NCBI-GI:21225530] [NCBI-GeneID:1102691] [UniProt:Q9X7U6]
SCO7271	383	0.14950	-0.99		SC5H1.21c; ion channel subunit; [GeneDB:SCO7271] [NCBI-GI:21225548] [NCBI-GeneID:1102709] [UniProt:Q9X7S9]
SCO7291	230	0.05348	-1.28	metabolic process, catalytic activity	SC5F8.01c. SC5H1.01; serine/threonine protein kinase; [GeneDB:SCO7291] [NCBI-GI:32141327] [NCBI-GeneID:1102729] [UniProt:Q8CJK5]
SCO7336	326	0.10908	-1.36		pepA. SC4G10.15c; hypothetical protein; [GeneDB:SCO7336] [NCBI-GI:21225611] [NCBI-GeneID:1102774] [UniProt:O54206]
SCO7337	289	0.08325	-1.12		pepA2. SC4G10.16; hypothetical protein; [GeneDB:SCO7337] [NCBI-GI:21225612] [NCBI-GeneID:1102775] [UniProt:O54205]

SCO7408	307	0.09687	-1.16	transporter activity	SC6D11.04c; probable solute-binding lipoprotein.; K02027 multiple sugar transport system substrate-binding protein; [GeneDB:SCO7408] [NCBI-GI:21225678] [NCBI-GeneID:1102846] [UniProt:Q9L151]
SCO7472	214	0.04879	-1.27		paaB. SCBAC17A6.05; putative phenylacetic acid degradation protein PaaB; K02610 phenylacetic acid degradation protein; [GeneDB:SCO7472] [NCBI-GI:21225740] [NCBI-GeneID:1102910] [UniProt:Q93JC4]
SCO7473	353	0.13006	-1.01		paaC. SCBAC17A6.06; putative phenylacetic acid degradation protein PaaC; K02611 phenylacetic acid degradation protein; [GeneDB:SCO7473] [NCBI-GI:21225741] [NCBI-GeneID:1102911] [UniProt:Q93JC3]
SCO7474	342	0.11906	-1.01		paaD. SCBAC17A6.07; putative phenylacetic acid degradation protein PaaD; K02612 phenylacetic acid degradation protein; [GeneDB:SCO7474] [NCBI-GI:21225742] [NCBI-GeneID:1102912] [UniProt:Q93JC2]
SCO7475	365	0.14233	-0.95	metabolic process, response to stimulus	SCBAC17A6.08; putative phenylacetic acid degradation NADH oxidoreductase PaaE; K02613 phenylacetic acid degradation NADH oxidoreductase; [GeneDB:SCO7475] [NCBI-GI:21225743] [NCBI-GeneID:1102913] [UniProt:Q93JC1]
SCO7524	374	0.14626	-0.96		SCBAC25F8.16; hypothetical protein; [GeneDB:SCO7524] [NCBI-GI:21225790] [NCBI-GeneID:1102962] [UniProt:Q93IZ6]
SCO7573	126	0.01889	-1.86	metabolic process, regulation of biological process	SC5F1.27c; anti-sigma factor antagonist; [GeneDB:SCO7573] [NCBI-GI:21225837] [NCBI-GeneID:1103011] [UniProt:Q9F3A2]
SCO7612	106	0.01387	-1.75		SC2H2.10c; hypothetical protein; [GeneDB:SCO7612] [NCBI-GI:21225874] [NCBI-GeneID:1103050] [UniProt:Q9F3E5]
SCO7643	238	0.05908	-1.56		SC10F4.16; hypothetical protein; [GeneDB:SCO7643] [NCBI-GI:21225904] [NCBI-GeneID:1103081] [UniProt:Q9F3P4]
SCO7700	223	0.05224	-1.26	metabolic process, catalytic activity, metal ion binding	SC1A4.08. SCBAC12C8.01; cyclase; [GeneDB:SCO7700] [NCBI-GI:32141341] [NCBI-GeneID:1103138] [UniProt:Q9F1Y6]
SCO7701	245	0.06147	-1.33	metabolic process, catalytic activity	SCBAC12C8.02; methyltransferase; [GeneDB:SCO7701] [NCBI-GI:21225960] [NCBI-GeneID:1103139] [UniProt:Q9F1Y5]
SCO7726	165	0.03079	-1.46	metabolic process, catalytic activity	SC8D11.17; hydrolase; [GeneDB:SCO7726] [NCBI-GI:21225985] [NCBI-GeneID:1103164] [UniProt:Q9AK02]
SCO7727	218	0.05119	-1.25	metabolic process, regulation of biological process	SC8D11.18c; MarR-family regulatory protein; [GeneDB:SCO7727] [NCBI-GI:21225986] [NCBI-GeneID:1103165] [UniProt:Q9AK01]

SCO7786	241	0.05913	-1.23		SC5E9.34c; hypothetical protein; [GeneDB:SCO7786] [NCBI-GI:21226037] [NCBI-GeneID:1103223] [UniProt:Q9EWK7]
SCO7837	242	0.05905	-1.31		SC8E7.34c; membrane protein; [GeneDB:SCO7837] [NCBI-GI:21226086] [NCBI-GeneID:1103275] [UniProt:Q9S1Y4]
**Gene**	**Rank of up-regulated genes**	**pfp value**	**Log(2) Average**	**GO Biological Process / Molecular Function**	**Product/Function**
SCO0074	165	0.06436	1.28		SCJ11.03; hypothetical protein; [GeneDB:SCO0074] [NCBI-GI:21218648] [NCBI-GeneID:1095505] [UniProt:Q9RIA1]
SCO0140	131	0.04305	1.49	metabolic process, regulation of biological process, DNA binding, nucleotide binding	SCJ33.04; MerR-family transcriptional regulator; [GeneDB:SCO0140] [NCBI-GI:21218699] [NCBI-GeneID:1095564] [UniProt:Q9RIX3]
SCO0171	249	0.14510	0.95	metabolic process, catalytic activity, metal ion binding	SCJ1.20; nicotinate phosphoribosyltransferase [EC:2.4.2.11]; K00763 nicotinate phosphoribosyltransferase; [GeneDB:SCO0171] [NCBI-GI:21218728] [NCBI-GeneID:1095595] [UniProt:Q9RIZ4]
SCO0231	146	0.05226	1.32	metabolic process, structural molecule activity	SCJ9A.10c; small hydrophobic hypothetical protein; [GeneDB:SCO0231] [NCBI-GI:21218784] [NCBI-GeneID:1095655] [UniProt:Q9S1R0]
SCO0446	194	0.09897	1.33		SCF51A.24c; hypothetical protein; [GeneDB:SCO0446] [NCBI-GI:21218988] [NCBI-GeneID:1095869] [UniProt:Q9RL40]
SCO0472	256	0.14766	1.02		SCF76.12c; secreted protein; [GeneDB:SCO0472] [NCBI-GI:21219012] [NCBI-GeneID:1095895] [UniProt:Q9RJG2]
SCO0476	105	0.02524	1.47	metabolic process, catalytic activity	SCF76.16; ABC transport protein. ATP-binding subunit; K02074 zinc/manganese transport system ATP-binding protein; [GeneDB:SCO0476] [NCBI-GI:21219016] [NCBI-GeneID:1095899] [UniProt:Q9RJF8]
SCO0489	51	0.00549	1.91		SCF34.08c; hypothetical protein; K05375 MbtH protein; [GeneDB:SCO0489] [NCBI-GI:21219027] [NCBI-GeneID:1095912] [UniProt:Q9RK17]
SCO0498	139	0.04885	1.39	metabolic process, catalytic activity	SCF34.17c; putative peptide monooxygenase [EC:1.13.12.-]; K00468; [GeneDB:SCO0498] [NCBI-GI:21219036] [NCBI-GeneID:1095921] [UniProt:Q9RK08]
SCO0499	252	0.14464	1.07		SCF34.18; formyltransferase; [GeneDB:SCO0499] [NCBI-GI:21219037] [NCBI-GeneID:1095922] [UniProt:Q9RK07]
SCO0694	250	0.14480	1.03		SCF42.04; hypothetical protein; [GeneDB:SCO0694] [NCBI-GI:21219222] [NCBI-GeneID:1096117] [UniProt:Q9L2K9]

SCO0715	181	0.08193	1.15		SCF42.25c; hypothetical protein; [GeneDB:SCO0715] [NCBI-GI:21219241] [NCBI-GeneID:1096138] [UniProt:Q9L2J4]
SCO0859	158	0.05911	1.35		SCM2.12c; membrane protein; [GeneDB:SCO0859] [NCBI-GI:21219380] [NCBI-GeneID:1096282] [UniProt:Q9RCV2]
SCO0860	61	0.00885	1.91	metabolic process, catalytic activity, transport, metal ion binding	SCM2.13c; probable cation-transporting ATPase [EC:3.6.3.-]; K01552; [GeneDB:SCO0860] [NCBI-GI:21219381] [NCBI-GeneID:1096283] [UniProt:Q9RCV1]
SCO0861	12	0.00083	3.03		SCM2.14c; secreted protein; [GeneDB:SCO0861] [NCBI-GI:21219382] [NCBI-GeneID:1096284] [UniProt:Q9RCV0]
SCO0862	74	0.01149	1.84		SCM2.15c; integral membrane protein; [GeneDB:SCO0862] [NCBI-GI:21219383] [NCBI-GeneID:1096285] [UniProt:Q9RCU9]
SCO0863	37	0.00324	2.31		SCM2.16c; integral membrane protein; [GeneDB:SCO0863] [NCBI-GI:21219384] [NCBI-GeneID:1096286] [UniProt:Q9RCU8]
SCO0865	64	0.01094	1.84		SCM2.18c; integral-membrane protein; [GeneDB:SCO0865] [NCBI-GI:21219386] [NCBI-GeneID:1096288] [UniProt:Q9RCU6]
SCO0866	118	0.03280	1.56	metabolic process, regulation of biological process, DNA binding	SCM2.19c; probable ECF-family sigma factor; K03088 RNA polymerase sigma-70 factor. ECF subfamily; [GeneDB:SCO0866] [NCBI-GI:21219387] [NCBI-GeneID:1096289] [UniProt:Q9RCU5]
SCO0867	172	0.06936	1.23		SCM2.20c; integral-membrane protein; [GeneDB:SCO0867] [NCBI-GI:21219388] [NCBI-GeneID:1096290] [UniProt:Q9RCU4]
SCO0888	63	0.01063	1.77	metabolic process, catalytic activity	SCM1.21; secreted protein; [GeneDB:SCO0888] [NCBI-GI:21219408] [NCBI-GeneID:1096311] [UniProt:Q9RD22]
SCO0922	221	0.11615	1.23		SCM10.10c; putative reductase iron-sulfur protein [EC:1.3.99.1]; K00240 succinate dehydrogenase iron-sulfur protein; [GeneDB:SCO0922] [NCBI-GI:21219441] [NCBI-GeneID:1096345] [UniProt:Q9RCY8]
SCO0923	137	0.04730	1.42		SCM10.11c; putative reductase flavoprotein subunit [EC:1.3.99.1]; K00239 succinate dehydrogenase flavoprotein subunit; [GeneDB:SCO0923] [NCBI-GI:21219442] [NCBI-GeneID:1096346] [UniProt:Q9RCY7]
SCO0924	84	0.01619	1.66	metabolic process, catalytic activity	SCM10.12c; putative cytochrome B subunit [EC:1.3.99.1]; K00241 succinate dehydrogenase cytochrome b-556 subunit; [GeneDB:SCO0924] [NCBI-GI:21219443] [NCBI-GeneID:1096347] [UniProt:Q9RCY6]
SCO0958	186	0.08559	1.13	metabolic process, catalytic activity	SCM11.13c; hypothetical protein; [GeneDB:SCO0958] [NCBI-GI:21219476] [NCBI-GeneID:1096381] [UniProt:Q9RIU8]
SCO0973	206	0.10757	1.16		SCM11.28; integral membrane protein; [GeneDB:SCO0973] [NCBI-GI:21219491]

					[NCBI-GeneID:1096396] [UniProt:Q9RIT4]
SCO0988	232	0.12754	1.09	metabolic process, catalytic activity	2SCG2.01; acetyltransferase; [GeneDB:SCO0988] [NCBI-GI:21219505] [NCBI-GeneID:1096411] [UniProt:Q8R5N4]
SCO0999	50	0.00500	1.98	metabolic process, catalytic activity, metal ion binding	2SCG2.12c. sodF2; superoxide dismutase [EC:1.15.1.1]; K00518 superoxide dismutase; [GeneDB:SCO0999] [NCBI-GI:21219516] [NCBI-GeneID:1096422] [UniProt:Q9X469]
SCO1033	243	0.13519	1.11		SCG20A.13c; hypothetical protein; [GeneDB:SCO1033] [NCBI-GI:21219549] [NCBI-GeneID:1096456] [UniProt:Q9K3M7]
SCO1067	185	0.08476	1.18		SCG22.13; integral membrane transport protein; [GeneDB:SCO1067] [NCBI-GI:21219582] [NCBI-GeneID:1096490] [UniProt:Q9K432]
SCO1138	81	0.01333	1.68		2SCG38.31; putative tricarboxylic transport membrane protein; K07795 putative tricarboxylic transport membrane protein; [GeneDB:SCO1138] [NCBI-GI:21219652] [NCBI-GeneID:1096561] [UniProt:Q9EWY8]
SCO1139	119	0.03294	1.42		2SCG38.32; integral membrane protein; [GeneDB:SCO1139] [NCBI-GI:21219653] [NCBI-GeneID:1096562] [UniProt:Q9EWY7]
SCO1140	151	0.05556	1.29		2SCG38.33; putative integral membrane protein; K07793 putative tricarboxylic transport membrane protein; [GeneDB:SCO1140] [NCBI-GI:21219654] [NCBI-GeneID:1096563] [UniProt:Q9EWY6]
SCO1250	33	0.00303	2.17	metabolic process, catalytic activity	2SCG1.25; acetyltransferase; [GeneDB:SCO1250] [NCBI-GI:21219759] [NCBI-GeneID:1096673] [UniProt:Q9FCB7]
SCO1364	115	0.03035	1.43	metabolic process	SC10A9.06c; hypothetical protein; [GeneDB:SCO1364] [NCBI-GI:21219869] [NCBI-GeneID:1096787] [UniProt:Q9AK98]
SCO1523	208	0.11111	1.18		SCL2.13c; pyridoxine biosynthesis protein [EC:4.-.-.-]; K06215 pyridoxine biosynthesis protein; [GeneDB:SCO1523] [NCBI-GI:21220023] [NCBI-GeneID:1096949] [UniProt:Q9L286]
SCO1550	219	0.11288	1.14		SCL11.06c; small membrane protein; [GeneDB:SCO1550] [NCBI-GI:21220049] [NCBI-GeneID:1096981] [UniProt:Q9L1D2]
SCO1658	236	0.13038	1.11	metabolic process, regulation of biological process, DNA binding	gylR. SCI46.03; glycerol operon regulatory protein; [GeneDB:SCO1658] [NCBI-GI:21220154] [NCBI-GeneID:1097089] [UniProt:P15360]
SCO1679	40	0.00300	1.98	metabolic process, catalytic activity, nucleotide binding	SCI52.21; putative gluconokinase [EC:2.7.1.12]; K00851 gluconokinase; [GeneDB:SCO1679] [NCBI-GI:21220175] [NCBI-GeneID:1097110] [UniProt:Q9AD88]
SCO1680	36	0.00333	1.99	metabolic process, catalytic activity	SCI30A.01. SCI52.22; putative gluconate permease; K03299 gluconate:H+ symporter. GntP family; [GeneDB:SCO1680] [NCBI-GI:32141150] [NCBI-

					GeneID:1097111] [UniProt:Q8CK22]
SCO1713	73	0.01164	1.76		SCI11.02c; hypothetical protein; [GeneDB:SCO1713] [NCBI-GI:21220207] [NCBI-GeneID:1097144] [UniProt:Q9S2B7]
SCO1714	238	0.13013	1.05		SCI11.03c; secreted protein; [GeneDB:SCO1714] [NCBI-GI:21220208] [NCBI-GeneID:1097145] [UniProt:Q9S2B6]
SCO1715	193	0.09715	1.13	metabolic process, catalytic activity	hgd. SCI11.04; putative homogentisate 1.2-dioxygenase [EC:1.13.11.5]; K00451 homogentisate 1.2-dioxygenase; [GeneDB:SCO1715] [NCBI-GI:21220209] [NCBI-GeneID:1097146] [UniProt:Q9S2B5]
SCO1792	128	0.04211	1.31	metabolic process, response to stimulus, DNA binding, catalytic activity	SCI51.32c; putative 3-methyladenine DNA glycosylase [EC:3.2.2.-]; K03652 3-methyladenine DNA glycosylase; [GeneDB:SCO1792] [NCBI-GI:21220284] [NCBI-GeneID:1097226] [UniProt:Q9S208]
SCO1947	68	0.01162	1.67		gap1. SCC54.07c; glyceraldehyde-3-phosphate dehydrogenase [EC:1.2.1.12]; K00134 glyceraldehyde 3-phosphate dehydrogenase; [GeneDB:SCO1947] [NCBI-GI:21220432] [NCBI-GeneID:1097381] [UniProt:Q9Z518]
SCO2014	46	0.00457	1.89	metabolic process, catalytic activity, metal ion binding	pyk1. SC7H2.28c; pyruvate kinase [EC:2.7.1.40]; K00873 pyruvate kinase; [GeneDB:SCO2014] [NCBI-GI:21220496] [NCBI-GeneID:1097448] [UniProt:Q9S2I9]
SCO2019	176	0.07773	1.25	metabolic process	SC7H2.33c; putative chorismate mutase [EC:5.4.99.5]; K04092 chorismate mutase; [GeneDB:SCO2019] [NCBI-GI:21220501] [NCBI-GeneID:1097453] [UniProt:Q9S2I4]
SCO2025	11	0.00091	2.96	metabolic process, catalytic activity, nucleotide binding	gltD. SC3A3.03c; putative glutamate synthase small subunit [EC:1.4.1.13]; K00266 glutamate synthase (NADPH) small chain; [GeneDB:SCO2025] [NCBI-GI:21220506] [NCBI-GeneID:1097459] [UniProt:Q9S2Z0]
SCO2026	9	0.00111	3.09	metabolic process, catalytic activity, protein binding	gltB. SC3A3.04c; putative glutamate synthase large subunit [EC:1.4.1.13]; K00265 glutamate synthase (NADPH) large chain; [GeneDB:SCO2026] [NCBI-GI:21220507] [NCBI-GeneID:1097460] [UniProt:Q9S2Y9]
SCO2114	242	0.13570	1.07		SC6E10.08c; hypothetical protein; [GeneDB:SCO2114] [NCBI-GI:21220592] [NCBI-GeneID:1097548] [UniProt:Q9S2M9]
SCO2146	49	0.00510	2.14	metabolic process, catalytic activity	SC6G10.19c; aminotransferase; [GeneDB:SCO2146] [NCBI-GI:21220623] [NCBI-GeneID:1097580] [UniProt:Q9X805]
SCO2164	136	0.04441	1.29		SC5F7.37c. St6G10A.02c; putative integral membrane efflux protein; K03296 hydrophobic/amphiphilic exporter-1 (mainly G- bacteria). HAE1 family; [GeneDB:SCO2164] [NCBI-GI:32141159] [NCBI-GeneID:1097597] [UniProt:Q8CK05]
SCO2210	226	0.12000	1.10	metabolic process, catalytic activity,	glnII. SC10B7.05; glutamine synthetase [EC:6.3.1.2]; K01915 glutamine synthetase; [GeneDB:SCO2210] [NCBI-GI:21220682] [NCBI-GeneID:1097643] [UniProt:Q9X958]

				nucleotide binding	
SCO2305	52	0.00577	2.03	metabolic process, catalytic activity, nucleotide binding	SCC30.13; putative ABC transporter ATP-binding subunit; K09687 antibiotic transport system ATP-binding protein; [GeneDB:SCO2305] [NCBI-GI:21220774] [NCBI-GeneID:1097739] [UniProt:Q9L008]
SCO2306	17	0.00059	2.62		SCC30.14; putative ABC transporter integral membrane protein; K01992 ABC-2 type transport system permease protein; [GeneDB:SCO2306] [NCBI-GI:21220775] [NCBI-GeneID:1097740] [UniProt:Q9L007]
SCO2307	195	0.10405	1.22	cell communication, metabolic process, nucleotide binding, signal transducer	SCC30.15; two-component sensor kinase; [GeneDB:SCO2307] [NCBI-GI:21220776] [NCBI-GeneID:1097741] [UniProt:Q9L006]
SCO2346	35	0.00314	1.99	metabolic process, catalytic activity	SCC8A.04c; putative hydrolase [EC:3.1.3.-]; K01112; [GeneDB:SCO2346] [NCBI-GI:21220814] [NCBI-GeneID:1097780] [UniProt:Q9KY44]
SCO2383	178	0.08146	1.17		SC4A7.11; secreted protein; [GeneDB:SCO2383] [NCBI-GI:21220850] [NCBI-GeneID:1097817] [UniProt:Q9RDQ1]
SCO2463	132	0.04371	1.51	metabolic process, catalytic activity, nucleotide binding, transporter activity	SC7A8.02; putative ABC transporter; K06147 ATP-binding cassette. subfamily B. bacterial; [GeneDB:SCO2463] [NCBI-GI:21220926] [NCBI-GeneID:1097897] [UniProt:Q9L2F3]
SCO2464	254	0.14713	1.20	metabolic process, catalytic activity, nucleotide binding, transporter activity	SC7A8.03; putative ABC transporter; K06147 ATP-binding cassette. subfamily B. bacterial; [GeneDB:SCO2464] [NCBI-GI:21220927] [NCBI-GeneID:1097898] [UniProt:Q9L2F2]
SCO2489	190	0.09111	1.16	metabolic process, regulation of biological process, DNA binding	SC7A8.28; TetR-family transcriptional regulator; [GeneDB:SCO2489] [NCBI-GI:21220952] [NCBI-GeneID:1097923] [UniProt:Q9L2D0]
SCO2505	100	0.02400	1.48		SCC121.08; putative ABC-transporter metal-binding lipoprotein; K09815 zinc transport system substrate-binding protein; [GeneDB:SCO2505] [NCBI-GI:21220966] [NCBI-GeneID:1097939] [UniProt:Q9L2H8]
SCO2528	39	0.00308	2.29	metabolic process, catalytic activity	leuA. SCC121.31c; 2-ispoprylmalate synthase [EC:2.3.3.13]; K01649 2-isopropylmalate synthase; [GeneDB:SCO2528] [NCBI-GI:32141173] [NCBI-GeneID:1097962] [UniProt:O31046]
SCO2591	91	0.02033	1.52		SCC88.02; secreted protein; [GeneDB:SCO2591] [NCBI-GI:21221049] [NCBI-GeneID:1098025] [UniProt:Q9L1I5]
SCO2633	21	0.00190	2.42	metabolic process,	SC8E4A.03. sodF; superoxide dismutase [Fe-Zn] [EC:1.15.1.1]; K00518 superoxide

				catalytic activity, metal ion binding	dismutase; [GeneDB:SCO2633] [NCBI-GI:21221090] [NCBI-GeneID:1098067] [UniProt:O51917]
SCO2683	134	0.04358	1.41	metabolic process, DNA binding	SCC61A.04c; single-strand DNA-binding protein; [GeneDB:SCO2683] [NCBI-GI:21221138] [NCBI-GeneID:1098117] [UniProt:Q9KYI9]
SCO2722	200	0.10665	1.16		SCC46.07c; putative integral membrane protein; K01992 ABC-2 type transport system permease protein; [GeneDB:SCO2722] [NCBI-GI:21221176] [NCBI-GeneID:1098156] [UniProt:Q9L1J4]
SCO2762	216	0.11213	1.14		SCC57A.33c; secreted protein; [GeneDB:SCO2762] [NCBI-GI:21221214] [NCBI-GeneID:1098196] [UniProt:Q9RDG6]
SCO2784	121	0.03421	1.37		SCC105.15; aceytltranferase; [GeneDB:SCO2784] [NCBI-GI:21221235] [NCBI-GeneID:1098218] [UniProt:Q9L070]
SCO2839	95	0.02000	1.73		SCE20.13c; lipoprotein; [GeneDB:SCO2839] [NCBI-GI:21221289] [NCBI-GeneID:1098273] [UniProt:Q9RDB2]
SCO2861	207	0.10768	1.28		SCE20.35.; hypothetical protein; [GeneDB:SCO2861] [NCBI-GI:21221311] [NCBI-GeneID:1098295] [UniProt:Q9RD90]
SCO2907	202	0.10688	1.07		SCE19A.07; putative PTS transmembrane component [EC:2.7.1.69]; K02803 PTS system. N-acetylglucosamine-specific IIB component; K02804 PTS system. N-acetylglucosamine-specific IIC component; [GeneDB:SCO2907] [NCBI-GI:21221354] [NCBI-GeneID:1098340] [UniProt:Q9S2H4]
SCO2935	233	0.12760	1.07	metabolic process, DNA binding	SCE19A.35c; transcriptional regulator; [GeneDB:SCO2935] [NCBI-GI:21221382] [NCBI-GeneID:1098368] [UniProt:Q9R3R2]
SCO2950	201	0.10741	1.08	cell organization and biogenesis, metabolic process, DNA binding	hup. SCE59.09c; DNA-binding protein Hu (hs1); [GeneDB:SCO2950] [NCBI-GI:21221395] [NCBI-GeneID:1098383] [UniProt:P0A3H5]
SCO3035	253	0.14735	0.97	metabolic process, catalytic activity, metal ion binding	SCE34.16; hypothetical protein; [GeneDB:SCO3035] [NCBI-GI:21221478] [NCBI-GeneID:1098468] [UniProt:Q9KZL0]
SCO3074	223	0.11834	1.07		SCE25.15c; integral membrane protein; [GeneDB:SCO3074] [NCBI-GI:21221515] [NCBI-GeneID:1098507] [UniProt:Q9KZ74]
SCO3089	26	0.00269	2.47	metabolic process, catalytic activity, nucleotide binding	SCE25.30; putative ABC transporter ATP-binding protein; K02003; [GeneDB:SCO3089] [NCBI-GI:21221529] [NCBI-GeneID:1098523] [UniProt:Q9KZ60]
SCO3090	31	0.00290	2.37		SCE25.31; putative ABC transporter integral membrane protein; K02004; [GeneDB:SCO3090] [NCBI-GI:21221530] [NCBI-GeneID:1098524] [UniProt:Q9KZ59]
SCO3091	168	0.06506	1.32		SCE25.32c; cyclopropane-fatty-acyl-phospholipid synthase [EC:2.1.1.79]; K00574

					cyclopropane-fatty-acyl-phospholipid synthase; [GeneDB:SCO3091] [NCBI-GI:21221531] [NCBI-GeneID:1098525] [UniProt:Q9KZ58]
SCO3096	169	0.06645	1.21	metabolic process, catalytic activity, metal ion binding	eno. SCE41.05c; enolase [EC:4.2.1.11]; K01689 enolase; [GeneDB:SCO3096] [NCBI-GI:21221535] [NCBI-GeneID:1098530] [UniProt:Q9F2Q3]
SCO3111	114	0.03035	1.55	metabolic process, catalytic activity, nucleotide binding	SCE41.20c; putative ABC transport system ATP-binding protein; K02003; [GeneDB:SCO3111] [NCBI-GI:21221550] [NCBI-GeneID:1098545] [UniProt:Q9F2N9]
SCO3151	78	0.01321	1.81	metabolic process, catalytic activity	SCE87.02c; putative deoxyribonuclease [EC:3.1.21.-]; K03424 Mg-dependent DNase; [GeneDB:SCO3151] [NCBI-GI:21221588] [NCBI-GeneID:1098585] [UniProt:Q9RKD6]
SCO3152	5	0.00000	3.59		SCE87.03c; hypothetical protein; [GeneDB:SCO3152] [NCBI-GI:21221589] [NCBI-GeneID:1098586] [UniProt:Q9RKD5]
SCO3185	239	0.13226	1.08		SCE22.02; putative Na+/H+ antiporter; K03316 monovalent cation:H+ antiporter. CPA1 family; [GeneDB:SCO3185] [NCBI-GI:21221620] [NCBI-GeneID:1098619] [UniProt:Q9KYW0]
SCO3306	210	0.11048	1.08	metabolic process, catalytic activity,	SCE68.04c; aminotransferase; [GeneDB:SCO3306] [NCBI-GI:21221737] [NCBI-GeneID:1098740] [UniProt:Q9WX27]
SCO3332	154	0.05838	1.35		SCE68.30c; hypothetical protein; [GeneDB:SCO3332] [NCBI-GI:21221763] [NCBI-GeneID:1098766] [UniProt:Q9WX02]
SCO3338	164	0.06384	1.19		SCE7.05c; putative integral membrane protein; K01992 ABC-2 type transport system permease protein; [GeneDB:SCO3338] [NCBI-GI:21221768] [NCBI-GeneID:1098775] [UniProt:Q9X8G2]
SCO3345	89	0.01989	1.69	metabolic process, catalytic activity,	SCE7.12c; dihydroxy acid dehydratase [EC:4.2.1.9]; K01687 dihydroxy-acid dehydratase; [GeneDB:SCO3345] [NCBI-GI:21221775] [NCBI-GeneID:1098782] [UniProt:O69198]
SCO3361	258	0.15050	1.01		SCE94.12c; AsnC-family transcriptional regulator; [GeneDB:SCO3361] [NCBI-GI:21221790] [NCBI-GeneID:1098798] [UniProt:Q9ZEP1]
SCO3375	222	0.11685	1.06		SCE94.26c; Lsr2-like protein; [GeneDB:SCO3375] [NCBI-GI:21221804] [NCBI-GeneID:1098812] [UniProt:Q9X8N1]
SCO3390	98	0.02082	1.70		SCE126.08c; two component sensor kinase; [GeneDB:SCO3390] [NCBI-GI:21221818] [NCBI-GeneID:1098827] [UniProt:Q9X851]
SCO3423	231	0.12688	1.05		SCE9.30; regulator; [GeneDB:SCO3423] [NCBI-GI:21221850] [NCBI-GeneID:1098860] [UniProt:Q9X8K2]
SCO3427	28	0.00250	2.27	metabolic process,	rpmE. SCE9.34c; putative 50S ribosomal protein L31; K02909 large subunit

				structural molecule activity	ribosomal protein L31; [GeneDB:SCO3427] [NCBI-GI:21221854] [NCBI-GeneID:1098864] [UniProt:Q9X8K6]
SCO3429	174	0.07149	1.28	metabolic process, structural molecule activity	rpmB. SCE9.36; putative 50S ribosomal protein L28; K02902 large subunit ribosomal protein L28; [GeneDB:SCO3429] [NCBI-GI:21221856] [NCBI-GeneID:1098866] [UniProt:Q9X8K8]
SCO3649	215	0.11121	1.11		fba. SCH10.27c; putative fructose 1.6-bisphosphate aldolase [EC:4.1.2.13]; K01624 fructose-bisphosphate aldolase. class II; [GeneDB:SCO3649] [NCBI-GI:21222064] [NCBI-GeneID:1099085] [UniProt:Q9X8R6]
SCO3704	167	0.06431	1.34		SCH35.20c; putative substrate-binding transport protein; K02020 molybdate transport system substrate-binding protein; [GeneDB:SCO3704] [NCBI-GI:21222117] [NCBI-GeneID:1099140] [UniProt:Q9X8Y6]
SCO3705	159	0.05987	1.38		SCH35.19c; putative ABC transporter membrane subunit; K02018 molybdate transport system permease protein; [GeneDB:SCO3705] [NCBI-GI:21222118] [NCBI-GeneID:1099141] [UniProt:Q9X8Y7]
SCO3712	150	0.05413	1.30	metabolic process, catalytic activity,	SCH35.12c; hydrolase; [GeneDB:SCO3712] [NCBI-GI:21222125] [NCBI-GeneID:1099148] [UniProt:Q9X8Z4]
SCO3717	241	0.13618	1.12	metabolic process, catalytic activity, metal ion binding	SCH35.07; putative cation transport system component [EC:3.6.3.12]; K01547 K+-transporting ATPase ATPase B chain; [GeneDB:SCO3717] [NCBI-GI:21222130] [NCBI-GeneID:1099153] [UniProt:Q9X8Z9]
SCO3718	92	0.02011	1.67	metabolic process, catalytic activity, nucleotide binding, transporter activity	SCH35.06; putative cation transport system component [EC:3.6.3.12]; K01546 K+-transporting ATPase ATPase A chain; [GeneDB:SCO3718] [NCBI-GI:21222131] [NCBI-GeneID:1099154] [UniProt:Q9X900]
SCO3719	60	0.00900	1.89		SCH35.05; small membrane protein; [GeneDB:SCO3719] [NCBI-GI:21222132] [NCBI-GeneID:1099155] [UniProt:Q9X901]
SCO3724	101	0.02426	1.45		SCH22A.02c; hypothetical protein; [GeneDB:SCO3724] [NCBI-GI:21222136] [NCBI-GeneID:1099160] [UniProt:Q9L0Y0]
SCO3793	198	0.10520	1.14		SCAC2.01; hypothetical protein; [GeneDB:SCO3793] [NCBI-GI:21222204] [NCBI-GeneID:1099229] [UniProt:Q9F325]
SCO3810	217	0.11226	1.00	metabolic process, regulation of biological process, DNA binding	SCGD3.11c; GntR-family transcriptional regulator; [GeneDB:SCO3810] [NCBI-GI:21222220] [NCBI-GeneID:1099246] [UniProt:Q9XA67]
SCO3877	234	0.12731	1.02	metabolic process, catalytic activity, nucleotide binding	SCH18.14c; putative 6-phosphogluconate dehydrogenase [EC:1.1.1.44]; K00033 6-phosphogluconate dehydrogenase; [GeneDB:SCO3877] [NCBI-GI:21222285] [NCBI-GeneID:1099313] [UniProt:Q53917]

SCO3888	113	0.02982	1.42	metabolic process, catalytic activity,	StH24.10; hypothetical protein; [GeneDB:SCO3888] [NCBI-GI:21222295] [NCBI-GeneID:1099324] [UniProt:Q9R3S2]
SCO3899	93	0.02011	1.59		SCH24.21c; myo-inositol-1-phosphate synthase [EC:5.5.1.4]; K01858 myo-inositol-1-phosphate synthase; [GeneDB:SCO3899] [NCBI-GI:21222306] [NCBI-GeneID:1099335] [UniProt:Q9X8T5]
SCO3900	102	0.02441	1.54		SCH24.22c; hypothetical protein; [GeneDB:SCO3900] [NCBI-GI:21222307] [NCBI-GeneID:1099336] [UniProt:Q9X8T6]
SCO3910	157	0.05904	1.27		SCH24.32c; hypothetical protein; [GeneDB:SCO3910] [NCBI-GI:21222317] [NCBI-GeneID:1099346] [UniProt:Q9X8U6]
SCO3918	261	0.15215	1.19	metabolic process, catalytic activity	SCH24.40c. SCQ11.01c; hypothetical protein; [GeneDB:SCO3918] [NCBI-GI:32141220] [NCBI-GeneID:1099354] [UniProt:Q8CJV3]
SCO3943	191	0.09063	1.14	metabolic process, regulation of biological process, DNA binding	rstP. SCD78.10c; transcriptional regulator; [GeneDB:SCO3943] [NCBI-GI:21222348] [NCBI-GeneID:1099379] [UniProt:O87640]
SCO3956	147	0.05211	1.34	metabolic process, catalytic activity, nucleotide binding	SCD78.23; putative ABC transporter ATP-binding protein; K01990 ABC-2 type transport system ATP-binding protein; [GeneDB:SCO3956] [NCBI-GI:21222361] [NCBI-GeneID:1099392] [UniProt:Q9ZBX6]
SCO3957	153	0.05758	1.31		SCD78.24; possible integral membrane protein; K01992 ABC-2 type transport system permease protein; [GeneDB:SCO3957] [NCBI-GI:21222362] [NCBI-GeneID:1099393] [UniProt:Q9ZBX5]
SCO3958	183	0.08421	1.21	metabolic process, catalytic activity, nucleotide binding	SCD78.25; ABC transporter ATP-binding protein; K01990 ABC-2 type transport system ATP-binding protein; [GeneDB:SCO3958] [NCBI-GI:21222363] [NCBI-GeneID:1099394] [UniProt:Q9ZBX4]
SCO3959	135	0.04444	1.41		SCD78.26; possible integral membrane protein; K01992 ABC-2 type transport system permease protein; [GeneDB:SCO3959] [NCBI-GI:21222364] [NCBI-GeneID:1099395] [UniProt:Q9ZBX3]
SCO3967	140	0.04857	1.38		SCBAC25E3.04c; conserved hypothetical membrane protein; K07040; [GeneDB:SCO3967] [NCBI-GI:21222371] [NCBI-GeneID:1099403] [UniProt:Q93J39]
SCO3968	189	0.08952	1.21		SCBAC25E3.05c; integral membrane protein; [GeneDB:SCO3968] [NCBI-GI:21222372] [NCBI-GeneID:1099404] [UniProt:Q93J38]
SCO3993	260	0.15200	1.06		SCBAC25E3.30c; hypothetical protein; [GeneDB:SCO3993] [NCBI-GI:21222396] [NCBI-GeneID:1099429] [UniProt:Q93J15]
SCO4005	47	0.00447	2.10	metabolic process, regulation of biological process, DNA binding	2SC10A7.09; RNA polymerase sigma factor; [GeneDB:SCO4005] [NCBI-GI:21222408] [NCBI-GeneID:1099441] [UniProt:Q9ADQ0]

SCO4020	156	0.05827	1.24	cell communication, metabolic process regulation, DNA binding, signal transducer activity	2SC10A7.24; putative two component system response regulator; K02483 two-component system. OmpR family. response regulator; [GeneDB:SCO4020] [NCBI-GI:21222423] [NCBI-GeneID:1099456] [UniProt:Q9ADN7]
SCO4021	182	0.08236	1.16	cell communication, metabolic process regulation, DNA binding, signal transducer activity	2SC10A7.25; putative two component system histidine kinase [EC:2.7.13.3]; K02484 two-component system. OmpR family. sensor kinase; [GeneDB:SCO4021] [NCBI-GI:21222424] [NCBI-GeneID:1099457] [UniProt:Q9ADN6]
SCO4054	214	0.11098	1.17		2SCD60.20; integral membrane protein; [GeneDB:SCO4054] [NCBI-GI:21222457] [NCBI-GeneID:1099490] [UniProt:Q9AK63]
SCO4157	235	0.12736	1.14	metabolic process, catalytic activity	SCD84.24c; protease; [GeneDB:SCO4157] [NCBI-GI:21222555] [NCBI-GeneID:1099597] [UniProt:Q9KZU4]
SCO4158	175	0.07303	1.17	metabolic process, regulation of biological process, DNA binding	SCD84.25c; putative LacI-family regulatory protein; [GeneDB:SCO4158] [NCBI-GI:21222556] [NCBI-GeneID:1099598] [UniProt:Q05954]
SCO4159	38	0.00316	2.03	cell communication, metabolic process regulation, DNA binding, signal transducer activity	glnR. SCD84.26c; transcriptional regulatory protein; [GeneDB:SCO4159] [NCBI-GI:21222557] [NCBI-GeneID:1099599] [UniProt:Q05943]
SCO4244	213	0.11061	1.13		SCD8A.17c; hypothetical protein; [GeneDB:SCO4244] [NCBI-GI:21222639] [NCBI-GeneID:1099684] [UniProt:Q9L0P7]
SCO4245	177	0.07768	1.27	metabolic process, metal ion binding	SCD8A.18c; hypothetical protein; K06903; [GeneDB:SCO4245] [NCBI-GI:21222640] [NCBI-GeneID:1099685] [UniProt:Q9L0P6]
SCO4246	143	0.05140	1.38		SCD8A.19c; hypothetical protein; [GeneDB:SCO4246] [NCBI-GI:21222641] [NCBI-GeneID:1099686] [UniProt:Q9L0P5]
SCO4247	77	0.01169	1.81	metabolic process	SCD8A.20c; hypothetical protein; [GeneDB:SCO4247] [NCBI-GI:21222642] [NCBI-GeneID:1099687] [UniProt:Q9L0P4]
SCO4248	54	0.00648	1.94		SCD8A.21c; hypothetical protein; [GeneDB:SCO4248] [NCBI-GI:21222643] [NCBI-GeneID:1099688] [UniProt:Q9L0P3]
SCO4251	32	0.00313	2.14		SCD8A.24c; secreted protein; [GeneDB:SCO4251] [NCBI-GI:21222646] [NCBI-GeneID:1099691] [UniProt:Q9L0P0]
SCO4252	56	0.00679	1.93		SCD8A.25c; hypothetical protein; [GeneDB:SCO4252] [NCBI-GI:21222647] [NCBI-

					GeneID:1099692] [UniProt:Q9L0N9]
SCO4253	42	0.00381	1.99		SCD8A.26c; hypothetical protein; K06907; [GeneDB:SCO4253] [NCBI-GI:21222648] [NCBI-GeneID:1099693] [UniProt:Q9L0N8]
SCO4256	109	0.02688	1.56		SCD8A.29; hydrolytic protein; [GeneDB:SCO4256] [NCBI-GI:21222651] [NCBI-GeneID:1099696] [UniProt:Q9L0N5]
SCO4257	96	0.02010	1.60		SCD8A.30; hydrolytic protein; [GeneDB:SCO4257] [NCBI-GI:21222652] [NCBI-GeneID:1099697] [UniProt:Q9L0N4]
SCO4258	88	0.01920	1.68		SCD8A.31; hydrolytic protein; [GeneDB:SCO4258] [NCBI-GI:21222653] [NCBI-GeneID:1099698] [UniProt:Q9L0N3]
SCO4259	192	0.09651	1.14		SCD8A.32c; AAA family ATPase; [GeneDB:SCO4259] [NCBI-GI:21222654] [NCBI-GeneID:1099699] [UniProt:Q9L0N2]
SCO4260	67	0.01179	1.68		SCD49.01c. SCD8A.33c; hypothetical protein; [GeneDB:SCO4260] [NCBI-GI:32141231] [NCBI-GeneID:1099700] [UniProt:Q8CJU2]
SCO4261	124	0.03839	1.39	metabolic process, regulation of biological process, DNA binding	SCD49.02c; response regulator; [GeneDB:SCO4261] [NCBI-GI:21222655] [NCBI-GeneID:1099701] [UniProt:Q9K4F1]
SCO4289	125	0.03824	1.38		SCD95A.22; secreted protein; [GeneDB:SCO4289] [NCBI-GI:21222682] [NCBI-GeneID:1099729] [UniProt:Q9KXV2]
SCO4293	10	0.00100	3.15		SCD95A.26; putative threonine synthase [EC:4.2.3.1]; K01733 threonine synthase; [GeneDB:SCO4293] [NCBI-GI:21222686] [NCBI-GeneID:1099733] [UniProt:Q9KXU8]
SCO4294	15	0.00067	2.62		SCD95A.27; hypothetical protein; [GeneDB:SCO4294] [NCBI-GI:21222687] [NCBI-GeneID:1099734] [UniProt:Q9KXU7]
SCO4366	130	0.04300	1.45	metabolic process, catalytic activity	SCD19.21c; putative phosphoserine aminotransferase [EC:2.6.1.52]; K00831 phosphoserine aminotransferase; [GeneDB:SCO4366] [NCBI-GI:21222757] [NCBI-GeneID:1099806] [UniProt:Q9F2Y1]
SCO4368	59	0.00831	1.96	metabolic process, catalytic activity	SCD19.23c; lipase (secreted protein); [GeneDB:SCO4368] [NCBI-GI:21222759] [NCBI-GeneID:1099808] [UniProt:Q9F2X9]
SCO4464	94	0.02000	1.50	metabolic process, catalytic activity	SCD65.07c; hydrolase; [GeneDB:SCO4464] [NCBI-GI:21222851] [NCBI-GeneID:1099904] [UniProt:Q9F2S5]
SCO4471	229	0.12358	1.10		SCD65.14; secreted protein; [GeneDB:SCO4471] [NCBI-GI:21222858] [NCBI-GeneID:1099911] [UniProt:Q9F2R8]
SCO4495	211	0.11104	1.13		SCD35.02; putative DNA polymerase related protein [EC:2.7.7.7]; K02334 DNA polymerase bacteriophage-type; [GeneDB:SCO4495] [NCBI-GI:21222880] [NCBI-GeneID:1099935] [UniProt:Q9L0U9]

SCO4584	227	0.11960	1.13		SCD20.02; hypothetical protein; [GeneDB:SCO4584] [NCBI-GI:21222967] [NCBI-GeneID:1100024] [UniProt:Q9F2X7]
SCO4640	209	0.11062	1.11	metabolic process, regulation of biological process, DNA binding	SCD82.11; TetR-family transcriptional regulator; [GeneDB:SCO4640] [NCBI-GI:21223022] [NCBI-GeneID:1100081] [UniProt:Q9L0M0]
SCO4657	259	0.15035	0.98	metabolic process, catalytic activity	SCD40A.03c; integral membrane protein; [GeneDB:SCO4657] [NCBI-GI:21223038] [NCBI-GeneID:1100098] [UniProt:Q9L0K6]
SCO4683	85	0.01600	1.57	metabolic process, catalytic activity	gdhA. SCD31.08; NADP-specific glutamate dehydrogenase [EC:1.4.1.4]; K00262 glutamate dehydrogenase (NADP+); [GeneDB:SCO4683] [NCBI-GI:21223063] [NCBI-GeneID:1100124] [UniProt:Q9L0F9]
SCO4762	228	0.12026	1.04	metabolic process, nucleotide binding protein, protein binding	groEL1; 60 kD chaperonin cpn60; K04077 chaperonin GroEL; [GeneDB:SCO4762] [NCBI-GI:21223141] [NCBI-GeneID:1100203] [UniProt:P40171]
SCO4850	251	0.14434	1.06	metabolic process, regulation of biological process, DNA binding	SC5G8.18c; TetR-family transcriptional regulator; [GeneDB:SCO4850] [NCBI-GI:21223226] [NCBI-GeneID:1100291] [UniProt:Q9KZ96]
SCO4851	25	0.00200	2.35		SC5G8.19c; hypothetical protein; [GeneDB:SCO4851] [NCBI-GI:21223227] [NCBI-GeneID:1100292] [UniProt:Q9KZ95]
SCO4852	41	0.00390	2.23		SC5G8.20c; integral membrane protein; [GeneDB:SCO4852] [NCBI-GI:21223228] [NCBI-GeneID:1100293] [UniProt:Q9KZ94]
SCO4904	170	0.06671	1.20		2SCK8.30c; integral membrane protein; [GeneDB:SCO4904] [NCBI-GI:21223278] [NCBI-GeneID:1100345] [UniProt:Q9AK22]
SCO4943	248	0.14327	1.05		2SCK31.03; oxidoreductase; [GeneDB:SCO4943] [NCBI-GI:21223316] [NCBI-GeneID:1100384] [UniProt:Q9EWV3]
SCO4945	110	0.02664	1.51	metabolic process, catalytic activity, metal ion binding	2SCK31.05; putative dehydrogenase [EC:1.1.1.2]; K00002 alcohol dehydrogenase (NADP+); [GeneDB:SCO4945] [NCBI-GI:21223318] [NCBI-GeneID:1100386] [UniProt:Q9EWF1]
SCO4947	187	0.08529	0.98	metabolic process, catalytic activity, metal ion binding	2SCK31.07. narG3; nitrate reductase alpha chain NarG3 [EC:1.7.99.4]; K00370 nitrate reductase 1. alpha subunit; [GeneDB:SCO4947] [NCBI-GI:21223320] [NCBI-GeneID:1100388] [UniProt:Q9EWF3]
SCO4954	199	0.10508	1.09		2SCK31.14c; hypothetical protein 2SCK3114c; [GeneDB:SCO4954] [NCBI-GI:21223327] [NCBI-GeneID:1100395] [UniProt:Q9ADK6]
SCO4955	152	0.05776	1.25		2SCK31.15c; ATP/GTP-binding protein; [GeneDB:SCO4955] [NCBI-GI:21223328] [NCBI-GeneID:1100396] [UniProt:Q9ADK5]
SCO5031	142	0.05063	1.28	metabolic process,	ahpD. SCK7.04c; alkyl hydroperoxide reductase system hypothetical protein; K04756

				response to stimulus, catalytic activity	alkyl hydroperoxide reductase subunit D; [GeneDB:SCO5031] [NCBI-GI:21223404] [NCBI-GeneID:1100472] [UniProt:Q7AKI6]
SCO5032	123	0.03659	1.41	metabolic process, antioxidant activity, catalytic activity,	ahpC. SCK7.05c; alkyl hydroperoxide reductase [EC:1.11.1.15]; K03386 peroxiredoxin (alkyl hydroperoxide reductase subunit C); [GeneDB:SCO5032] [NCBI-GI:21223405] [NCBI-GeneID:1100473] [UniProt:Q9FBP5]
SCO5129	141	0.04943	1.41	metabolic process, catalytic activity, nucleotide binding	SC9E12.14; putative ABC transporter ATP-binding protein; K01990 ABC-2 type transport system ATP-binding protein; [GeneDB:SCO5129] [NCBI-GI:21223499] [NCBI-GeneID:1100570] [UniProt:Q9F341]
SCO5162	122	0.03467	1.38		SCP8.25; putative integral membrane protein; K05595 multiple antibiotic resistance protein; [GeneDB:SCO5162] [NCBI-GI:21223531] [NCBI-GeneID:1100603] [UniProt:Q9FBJ6]
SCO5229	171	0.06977	1.18		SC7E4.26c; putative permease; K03307 solute:Na+ symporter. SSS family; [GeneDB:SCO5229] [NCBI-GI:21223597] [NCBI-GeneID:1100670] [UniProt:Q9K494]
SCO5230	133	0.04346	1.31		SC7E4.27c; integral membrane protein; [GeneDB:SCO5230] [NCBI-GI:21223598] [NCBI-GeneID:1100671] [UniProt:Q9K493]
SCO5257	82	0.01524	1.65	metabolic process, catalytic activity	2SC7G11.19. metZ; methyltransferase; [GeneDB:SCO5257] [NCBI-GI:21223624] [NCBI-GeneID:1100698] [UniProt:Q9F3K8]
SCO5258	126	0.04143	1.34	metabolic process, catalytic activity, nucleotide binding	2SC7G11.20c. atrC; ATP-binding protein [EC:3.6.3.21]; K02028 polar amino acid transport system ATP-binding protein; [GeneDB:SCO5258] [NCBI-GI:21223625] [NCBI-GeneID:1100699] [UniProt:Q9F3K7]
SCO5285	255	0.14773	0.94	metabolic process, response o stimulus, DNA binding	lon. SCCB12.09; ATP-dependent protease [EC:3.4.21.53]; K01338 ATP-dependent Lon protease; [GeneDB:SCO5285] [NCBI-GI:21223651] [NCBI-GeneID:1100726] [UniProt:Q9EVK2]
SCO5299	104	0.02529	1.49		SC6G9.34; hypothetical protein; [GeneDB:SCO5299] [NCBI-GI:21223662] [NCBI-GeneID:1100739] [UniProt:Q9XAE8]
SCO5300	53	0.00604	2.06		SC6G9.33; hypothetical protein; [GeneDB:SCO5300] [NCBI-GI:21223663] [NCBI-GeneID:1100740] [UniProt:Q9XAE9]
SCO5379	220	0.11641	1.03	metabolic process, regulation of biological process	2SC6G5.23; hypothetical protein; [GeneDB:SCO5379] [NCBI-GI:21223739] [NCBI-GeneID:1100819] [UniProt:Q9K4D2]
SCO5413	247	0.13883	1.07	metabolic process, regulation of biological process, DNA binding	SC8F4.17; MarR-transcriptional regulator; [GeneDB:SCO5413] [NCBI-GI:21223773] [NCBI-GeneID:1100853] [UniProt:Q9L2A7]
SCO5423	16	0.00063	2.50	metabolic process, catalytic activity, metal	pyk2. SC8F4.27c; pyruvate kinase [EC:2.7.1.40]; K00873 pyruvate kinase; [GeneDB:SCO5423] [NCBI-GI:21223783] [NCBI-GeneID:1100863] [UniProt:Q9L299]

				ion binding	
SCO5468	218	0.11179	1.11		SC2A11.02c; transmembrane transport protein; [GeneDB:SCO5468] [NCBI-GI:21223825] [NCBI-GeneID:1100908] [UniProt:O86563]
SCO5512	127	0.04181	1.41	metabolic process, catalytic activity, metal ion binding, nucleotide binding	ilvB. SC8D9.24; acetolactate synthase large subunit [EC:2.2.1.6]; K01652 acetolactate synthase large subunit; [GeneDB:SCO5512] [NCBI-GI:21223868] [NCBI-GeneID:1100952] [UniProt:Q9Z567]
SCO5513	90	0.01989	1.60		ilvN. SC8D9.25; acetolactate synthase small subunit [EC:2.2.1.6]; K01653 acetolactate synthase small subunit; [GeneDB:SCO5513] [NCBI-GI:21223869] [NCBI-GeneID:1100953] [UniProt:Q9Z566]
SCO5514	225	0.12004	1.09	metabolic process, catalytic activity	ilvC. SC8D9.26; acetolactate synthase small subunit [EC:1.1.1.86]; K00053 ketol-acid reductoisomerase; [GeneDB:SCO5514] [NCBI-GI:21223870] [NCBI-GeneID:1100954] [UniProt:Q9Z565]
SCO5515	76	0.01171	1.70	metabolic process, catalytic activity, nucleotide binding	SC8D9.27. serA; probable D-3-phosphoglycerate dehydrogenase [EC:1.1.1.95]; K00058 D-3-phosphoglycerate dehydrogenase; [GeneDB:SCO5515] [NCBI-GI:21223871] [NCBI-GeneID:1100955] [UniProt:Q9Z564]
SCO5521	4	0.00000	3.34		SC1C2.02; hypothetical protein; [GeneDB:SCO5521] [NCBI-GI:21223876] [NCBI-GeneID:1100961] [UniProt:O86503]
SCO5522	7	0.00143	3.20	metabolic process, catalytic activity, nucleotide binding	leuB; 3-isopropylmalate dehydrogenase [EC:1.1.1.85]; K00052 3-isopropylmalate dehydrogenase; [GeneDB:SCO5522] [NCBI-GI:21223877] [NCBI-GeneID:1100962] [UniProt:O86504]
SCO5552	129	0.04194	1.34	metabolic process, regulation of biological process, DNA binding	SC1C2.33c; regulator; [GeneDB:SCO5552] [NCBI-GI:21223907] [NCBI-GeneID:1100992] [UniProt:O86533]
SCO5553	69	0.01159	1.94	metabolic process, catalytic activity, metal ion binding	leuC; 3-isopropylmalate dehydratase large subunit [EC:4.2.1.33]; K01703 3-isopropylmalate/(R)-2-methylmalate dehydratase large subunit; [GeneDB:SCO5553] [NCBI-GI:21223908] [NCBI-GeneID:1100993] [UniProt:O86534]
SCO5554	24	0.00208	2.46	metabolic process, catalytic activity	leuD; 3-isopropylmalate dehydratase small subunit [EC:4.2.1.33]; K01704 3-isopropylmalate/(R)-2-methylmalate dehydratase small subunit; [GeneDB:SCO5554] [NCBI-GI:21223909] [NCBI-GeneID:1100994] [UniProt:O86535]
SCO5578	2	0.00000	4.38		SC7A1.22; putative sugar transporter; K08139 MFS transporter. SP family. sugar:H+ symporter; [GeneDB:SCO5578] [NCBI-GI:21223934] [NCBI-GeneID:1101019] [UniProt:Q9ZBQ1]
SCO5583	144	0.05257	1.37		SC7A1.27; ammonium transporter; K03320 ammonium transporter. Amt family; [GeneDB:SCO5583] [NCBI-GI:21223939] [NCBI-GeneID:1101024] [UniProt:Q9ZBP6]

SCO5638	43	0.00419	2.15		SC6A9.29; integral membrane protein; [GeneDB:SCO5638] [NCBI-GI:21223989] [NCBI-GeneID:1101077] [UniProt:O86761]
SCO5640	108	0.02667	1.44		SC6A9.27; hypothetical protein; [GeneDB:SCO5640] [NCBI-GI:21223991] [NCBI-GeneID:1101079] [UniProt:O86759]
SCO5771	246	0.13858	1.15		SC4H8.10c; hypothetical protein; [GeneDB:SCO5771] [NCBI-GI:21224117] [NCBI-GeneID:1101213] [UniProt:O50489]
SCO5774	18	0.00111	2.41		gluD; glutamate permease; K10007 glutamate transport system permease protein; [GeneDB:SCO5774] [NCBI-GI:21224120] [NCBI-GeneID:1101216] [UniProt:O50492]
SCO5775	29	0.00241	2.17		gluC; glutamate permease; K10006 glutamate transport system permease protein; [GeneDB:SCO5775] [NCBI-GI:21224121] [NCBI-GeneID:1101217] [UniProt:O50493]
SCO5776	34	0.00294	2.15		gluB; glutamate binding protein; K10005 glutamate transport system substrate-binding protein; [GeneDB:SCO5776] [NCBI-GI:21224122] [NCBI-GeneID:1101218] [UniProt:O50494]
SCO5777	57	0.00754	1.89		gluA; glutamate uptake system ATP-binding protein [EC:3.6.3.-]; K10008 glutamate transport system ATP-binding protein; [GeneDB:SCO5777] [NCBI-GI:21224123] [NCBI-GeneID:1101219] [UniProt:O50495]
SCO5810	205	0.10785	1.07		SC4H2.31c; transmembrane efflux protein; [GeneDB:SCO5810] [NCBI-GI:21224155] [NCBI-GeneID:1101252] [UniProt:O69986]
SCO5830	197	0.10477	1.12		hypothetical protein; [GeneDB:SCO5830] [NCBI-GI:21224174] [NCBI-GeneID:1101272] [UniProt:O70006]
SCO5929	149	0.05409	1.26	metabolic process, catalytic activity	SC10A5.34c; oxidoreductase; [GeneDB:SCO5929] [NCBI-GI:21224268] [NCBI-GeneID:1101371] [UniProt:O54125]
SCO5982	80	0.01350	1.62		StBAC16H6.17c; regulator; K02616 phenylacetic acid degradation operon negative regulatory protein; [GeneDB:SCO5982] [NCBI-GI:21224319] [NCBI-GeneID:1101424] [UniProt:Q93JE5]
SCO6008	27	0.00259	2.14		SC7B7.05; probable transcriptional repressor protein; [GeneDB:SCO6008] [NCBI-GI:21224343] [NCBI-GeneID:1101450] [UniProt:O50502]
SCO6009	8	0.00125	3.23		SC7B7.06; solute-binding protein; K02058 simple sugar transport system substrate-binding protein; [GeneDB:SCO6009] [NCBI-GI:21224344] [NCBI-GeneID:1101451] [UniProt:O50503]
SCO6010	3	0.00000	3.58	metabolic process, catalytic activity, nucleotide binding	SC7B7.07; probable ABC-transport system ATP binding protein [EC:3.6.3.17]; K02056 simple sugar transport system ATP-binding protein; [GeneDB:SCO6010] [NCBI-GI:21224345] [NCBI-GeneID:1101452] [UniProt:O50504]
SCO6011	6	0.00000	3.31		SC7B7.08; probable ABC-type transmembrane transport protein; K02057 simple sugar transport system permease protein; [GeneDB:SCO6011] [NCBI-GI:21224346]

					[NCBI-GeneID:1101453] [UniProt:O50505]
SCO6044	244	0.13811	1.01		SC1B5.04c; integral membrane protein; [GeneDB:SCO6044] [NCBI-GI:21224375] [NCBI-GeneID:1101485] [UniProt:O69832]
SCO6109	173	0.06942	1.20	metabolic process, catalytic activity	SCBAC1A6.33; secreted hydrolase; [GeneDB:SCO6109] [NCBI-GI:21224438] [NCBI-GeneID:1101550] [UniProt:Q9ADF4]
SCO6124	240	0.13254	1.15		SC9B2.11; hypothetical protein; [GeneDB:SCO6124] [NCBI-GI:21224451] [NCBI-GeneID:1101565] [UniProt:Q9Z548]
SCO6272	120	0.03408	1.22	metabolic process, catalytic activity, nucleotide binding	SC2C4.02; secreted FAD-binding protein; [GeneDB:SCO6272] [NCBI-GI:21224592] [NCBI-GeneID:1101713] [UniProt:Q9EX55]
SCO6273	179	0.08123	0.92	metabolic process, catalytic activity	SC2C4.03c; type I polyketide synthase; [GeneDB:SCO6273] [NCBI-GI:21224593] [NCBI-GeneID:1101714] [UniProt:Q9EX54]
SCO6274	155	0.05813	0.94	metabolic process, catalytic activity	SC2C4.04c; type I polyketide synthase; [GeneDB:SCO6274] [NCBI-GI:21224594] [NCBI-GeneID:1101715] [UniProt:Q9EX53]
SCO6275	203	0.10714	0.85	metabolic process, catalytic activity	SC1G7.01c. SC2C4.05c; type I polyketide synthase; [GeneDB:SCO6275] [NCBI-GI:32141295] [NCBI-GeneID:1101716] [UniProt:Q8CJN6]
SCO6276	103	0.02485	0.66		SC1G7.02; secreted protein; [GeneDB:SCO6276] [NCBI-GI:21224595] [NCBI-GeneID:1101717] [UniProt:Q93S13]
SCO6277	111	0.02748	0.62	metabolic process, catalytic activity	SC1G7.03; epoxide hydrolase; [GeneDB:SCO6277] [NCBI-GI:21224596] [NCBI-GeneID:1101718] [UniProt:Q93S12]
SCO6278	75	0.01173	1.09		SC1G7.04; putative integral membrane transport protein; K08167 MFS transporter. DHA2 family. methyl viologen resistance protein SmvA; [GeneDB:SCO6278] [NCBI-GI:21224597] [NCBI-GeneID:1101719] [UniProt:Q93S11]
SCO6279	48	0.00521	1.03		SC1G7.05; diaminobutyrate-pyruvate aminotransferase; [GeneDB:SCO6279] [NCBI-GI:21224598] [NCBI-GeneID:1101720] [UniProt:Q93S10]
SCO6282	66	0.01167	0.94	metabolic process, catalytic activity	SC1G7.08c; 3-oxoacyl-[acyl-carrier protein] reductase; [GeneDB:SCO6282] [NCBI-GI:21224601] [NCBI-GeneID:1101723] [UniProt:Q93S07]
SCO6283	107	0.02477	1.13		SC1G7.09; hypothetical protein; [GeneDB:SCO6283] [NCBI-GI:21224602] [NCBI-GeneID:1101724] [UniProt:Q93S06]
SCO6284	230	0.12743	0.78		SC1G7.10; putative decarboxylase [EC:4.1.1.41]; K01604 methylmalonyl-CoA decarboxylase alpha chain; [GeneDB:SCO6284] [NCBI-GI:21224603] [NCBI-GeneID:1101725] [UniProt:Q93S05]
SCO6285	163	0.06423	0.94		SC1G7.11; hypothetical protein; [GeneDB:SCO6285] [NCBI-GI:21224604] [NCBI-GeneID:1101726] [UniProt:Q93S04]

SCO6320	161	0.06348	1.26		SCIF3.22c; transport integral membrane protein; [GeneDB:SCO6320] [NCBI-GI:21224636] [NCBI-GeneID:1101761] [UniProt:Q93RS4]
SCO6429	44	0.00432	2.00		SC1A6.18; hypothetical protein; [GeneDB:SCO6429] [NCBI-GI:21224736] [NCBI-GeneID:1101868] [UniProt:O69823]
SCO6430	184	0.08451	1.18		SC1A6.19; hypothetical protein; [GeneDB:SCO6430] [NCBI-GI:21224737] [NCBI-GeneID:1101869] [UniProt:O69824]
SCO6431	86	0.01872	1.74		SC1A6.20; peptide synthase; [GeneDB:SCO6431] [NCBI-GI:21224738] [NCBI-GeneID:1101870] [UniProt:O69825]
SCO6433	20	0.00200	2.54		SC1A6.22; hypothetical protein; K06995; [GeneDB:SCO6433] [NCBI-GI:21224740] [NCBI-GeneID:1101872] [UniProt:O69827]
SCO6434	83	0.01566	1.85	metabolic process, catalytic activity	SC1A6.23. SC9B5.01; oxidoreductase; [GeneDB:SCO6434] [NCBI-GI:32141301] [NCBI-GeneID:1101873] [UniProt:Q8CJN0]
SCO6435	70	0.01143	1.97	metabolic process, regulation of biological process	SC9B5.02; hypothetical protein; [GeneDB:SCO6435] [NCBI-GI:21224741] [NCBI-GeneID:1101874] [UniProt:Q9ZBH8]
SCO6436	58	0.00828	2.04	metabolic process, catalytic activity	SC9B5.03; tRNA synthetase; [GeneDB:SCO6436] [NCBI-GI:21224742] [NCBI-GeneID:1101875] [UniProt:Q9ZBH7]
SCO6437	116	0.03017	1.55		SC9B5.04; hypothetical protein; [GeneDB:SCO6437] [NCBI-GI:21224743] [NCBI-GeneID:1101876] [UniProt:Q9ZBH6]
SCO6438	55	0.00691	2.00		SC9B5.05; diaminopimelate decarboxylase [EC:4.1.1.20]; K01586 diaminopimelate decarboxylase; [GeneDB:SCO6438] [NCBI-GI:21224744] [NCBI-GeneID:1101877] [UniProt:Q9ZBH5]
SCO6451	145	0.05248	1.30		SC9B5.18; putative substrate binding protein; K02035 peptide/nickel transport system substrate-binding protein; [GeneDB:SCO6451] [NCBI-GI:21224757] [NCBI-GeneID:1101890] [UniProt:Q9ZBG2]
SCO6452	117	0.03085	1.40		SC9B5.19; putative transport permease protein; K02033 peptide/nickel transport system permease protein; [GeneDB:SCO6452] [NCBI-GI:21224758] [NCBI-GeneID:1101891] [UniProt:Q9ZBG1]
SCO6453	160	0.06338	1.20		SC9B5.20; putative transport permease protein; K02034 peptide/nickel transport system permease protein; [GeneDB:SCO6453] [NCBI-GI:21224759] [NCBI-GeneID:1101892] [UniProt:Q9ZBG0]
SCO6509	13	0.00077	2.79		SC1E6.18c; hydrophobic protein; [GeneDB:SCO6509] [NCBI-GI:21224812] [NCBI-GeneID:1101948] [UniProt:Q9ZC04]
SCO6510	22	0.00182	2.39	metabolic process, catalytic activity	SC1E6.19c; hypothetical protein; [GeneDB:SCO6510] [NCBI-GI:21224813] [NCBI-GeneID:1101949] [UniProt:Q9ZC03]

SCO6658	138	0.04833	1.28	metabolic process, catalytic activity, nucleotide binding	SC5A7.08c; 6-phosphogluconate dehydrogenase [EC:1.1.1.44]; K00033 6-phosphogluconate dehydrogenase; [GeneDB:SCO6658] [NCBI-GI:21224954] [NCBI-GeneID:1102097] [UniProt:O88014]
SCO6659	212	0.11104	1.10	metabolic process, catalytic activity	pgi. SC5A7.09c; glucose-6-phosphate isomerase [EC:5.3.1.9]; K01810 glucose-6-phosphate isomerase; [GeneDB:SCO6659] [NCBI-GI:21224955] [NCBI-GeneID:1102098] [UniProt:O88015]
SCO6660	99	0.02141	1.49		SC5A7.10c; hypothetical protein; [GeneDB:SCO6660] [NCBI-GI:21224956] [NCBI-GeneID:1102099] [UniProt:O88016]
SCO6661	106	0.02500	1.44	metabolic process, catalytic activity, nucleotide binding	SC5A7.11c. zwf; glucose-6-phosphate 1-dehydrogenase [EC:1.1.1.49]; K00036 glucose-6-phosphate 1-dehydrogenase; [GeneDB:SCO6661] [NCBI-GI:21224957] [NCBI-GeneID:1102100] [UniProt:O88017]
SCO6662	30	0.00267	2.11	metabolic process, catalytic activity, nucleotide binding	tal1; transaldolase [EC:2.2.1.2]; K00616 transaldolase; [GeneDB:SCO6662] [NCBI-GI:21224958] [NCBI-GeneID:1102101] [UniProt:O88018]
SCO6663	97	0.01990	1.53	metabolic process, catalytic activity	tktB; transketolase B [EC:2.2.1.1]; K00615 transketolase; [GeneDB:SCO6663] [NCBI-GI:21224959] [NCBI-GeneID:1102102] [UniProt:O88019]
SCO6990	72	0.01139	1.77		SC8F11.16c; putative membrane transport protein.; K03453 bile acid:Na+ symporter. BASS family; [GeneDB:SCO6990] [NCBI-GI:21225276] [NCBI-GeneID:1102428] [UniProt:Q9KZG2]
SCO7047	188	0.08846	1.17		SC4G1.13; undecaprenyl-diphosphatase [EC:3.6.1.27]; K06153 undecaprenyl-diphosphatase; [GeneDB:SCO7047] [NCBI-GI:21225330] [NCBI-GeneID:1102485] [UniProt:Q9FC36]
SCO7153	1	0.00000	4.48		SC9A4.15; putative sugar transporter; K08139 MFS transporter. SP family. sugar:H+ symporter; [GeneDB:SCO7153] [NCBI-GI:21225433] [NCBI-GeneID:1102591] [UniProt:Q9ZBQ1]
SCO7154	257	0.15058	1.02	metabolic process, catalytic activity	ilvC2. SC9A4.16c; ketol-acid reductoisomerase [EC:1.1.1.86]; K00053 ketol-acid reductoisomerase; [GeneDB:SCO7154] [NCBI-GI:21225434] [NCBI-GeneID:1102592] [UniProt:Q9FBT8]
SCO7199	62	0.01000	1.68		SC1D2.02c; membrane protein; [GeneDB:SCO7199] [NCBI-GI:21225477] [NCBI-GeneID:1102637] [UniProt:Q9K448]
SCO7200	87	0.01851	1.80	metabolic process, catalytic activity	SC1D2.03c; hypothetical protein; [GeneDB:SCO7200] [NCBI-GI:21225478] [NCBI-GeneID:1102638] [UniProt:Q9K447]
SCO7399	204	0.10706	1.15		SC10G8.27c; binding-protein-dependent transport lipoprotein; [GeneDB:SCO7399] [NCBI-GI:21225670] [NCBI-GeneID:1102837] [UniProt:Q9L178]
SCO7400	245	0.13882	1.06	metabolic process,	SC10G8.28c; putative ABC-transport protein. ATP-binding component. [EC:3.6.3.34];

				catalytic activity, nucleotide binding	K02013 iron complex transport system ATP-binding protein; [GeneDB:SCO7400] [NCBI-GI:21225671] [NCBI-GeneID:1102838] [UniProt:Q9L177]
SCO7417	224	0.12054	1.12		SC6D11.13c; putative cytochrome P450-family protein. [EC:1.14.-.-]; K00517; [GeneDB:SCO7417] [NCBI-GI:21225687] [NCBI-GeneID:1102855] [UniProt:Q9L142]
SCO7424	45	0.00444	1.91	metabolic process, regulation of biological process, DNA binding	SC6D11.20; MarR-family transcriptional regulator; [GeneDB:SCO7424] [NCBI-GI:21225694] [NCBI-GeneID:1102862] [UniProt:Q9L135]
SCO7428	196	0.10500	1.13		hmpA1; flavohemoprotein [EC:1.14.12.17]; K05916 nitric oxide dioxygenase; [GeneDB:SCO7428] [NCBI-GI:21225698] [NCBI-GeneID:1102866] [UniProt:Q9L131]
SCO7459	71	0.01127	1.78	metabolic process, catalytic activity, nucleotide binding	SC5C11.16c; putative ABC transport protein.ATP-binding component. [EC:3.6.3.34]; K02013 iron complex transport system ATP-binding protein; [GeneDB:SCO7459] [NCBI-GI:21225728] [NCBI-GeneID:1102897] [UniProt:Q9L156]
SCO7462	162	0.06364	1.22		SCBAC14E8.02c; putative integral membrane protein; K07090; [GeneDB:SCO7462] [NCBI-GI:21225731] [NCBI-GeneID:1102900] [UniProt:Q9ADJ7]
SCO7463	79	0.01316	1.60	metabolic process, catalytic activity, nucleotide binding	cvnA13. SCBAC14E8.03; sensor histidine kinase; [GeneDB:SCO7463] [NCBI-GI:21225732] [NCBI-GeneID:1102901] [UniProt:Q9ADJ6]
SCO7464	65	0.01154	1.70		cvnB13. SCBAC14E8.04; hypothetical protein; [GeneDB:SCO7464] [NCBI-GI:21225733] [NCBI-GeneID:1102902] [UniProt:Q9ADJ5]
SCO7465	148	0.05378	1.25		cvnC13. SCBAC14E8.05; hypothetical protein; [GeneDB:SCO7465] [NCBI-GI:21225734] [NCBI-GeneID:1102903] [UniProt:Q9ADJ4]
SCO7510	112	0.02902	1.45		cypH. SCBAC25F8.02c; peptidyl-prolyl cis-trans isomerase [EC:5.2.1.8]; K01802 peptidylprolyl isomerase; [GeneDB:SCO7510] [NCBI-GI:21225776] [NCBI-GeneID:1102948] [UniProt:Q93J09]
SCO7511	23	0.00217	2.25	metabolic process, catalytic activity, nucleotide binding	gap2. SCBAC25F8.03c; glyceraldehyde 3-phosphate dehydrogenase [EC:1.2.1.12]; K00134 glyceraldehyde 3-phosphate dehydrogenase; [GeneDB:SCO7511] [NCBI-GI:21225777] [NCBI-GeneID:1102949] [UniProt:Q93J08]
SCO7544	180	0.08128	1.19		SC8G12.20; putative ABC-transport protein. membrane component.; K02050 sulfonate/nitrate/taurine transport system permease protein; [GeneDB:SCO7544] [NCBI-GI:21225809] [NCBI-GeneID:1102982] [UniProt:Q9KYY5]
SCO7676	14	0.00071	2.87		SC4C2.11; ferredoxin; [GeneDB:SCO7676] [NCBI-GI:21225936] [NCBI-GeneID:1103114] [UniProt:Q9EWQ1]
SCO7695	166	0.06470	1.24		SC1A4.03c; hypothetical protein; [GeneDB:SCO7695] [NCBI-GI:21225955] [NCBI-GeneID:1103133] [UniProt:Q9EX60]
SCO7756	19	0.00105	2.56		SC5E9.04; hypothetical protein; [GeneDB:SCO7756] [NCBI-GI:21226012] [NCBI-

					GeneID:1103194] [UniProt:Q9EWN1]
SCO7799	237	0.13000	1.05		SC10B8A.09c; transposase; [GeneDB:SCO7799] [NCBI-GI:21226051] [NCBI-GeneID:1103237] [UniProt:Q9FCL5]

Table S2. Complete dataset of differentially expressed genes at statistically significant levels (pfp values ≤0.15) in the *Sco*M145 wild type strain relative to the *ScoZm* mutant.

Gene name	Rank of down-regulated genes	pfp value	Log(2) Average	GO Biological Process / Molecular Function	Product/Function
SCO0138	28	0.06464	-2.21	metabolic process, catalytic activity	SCJ33.02; short chain dehydrogenase; [GeneDB:SCO0138] [NCBI-GI:21218697] [NCBI-GeneID:1095562] [UniProt:Q9RIX5]
SCO0555	15	0.06800	-1.96	metabolic process, catalytic activity, metal ion binding	SCF73.02c; membrane-bound oxidoreductase; [GeneDB:SCO0555] [NCBI-GI:21219089] [NCBI-GeneID:1095978] [UniProt:Q9RJI4]
SCO0556	6	0.01667	-2.96		SCF73.03c; hypothetical protein; [GeneDB:SCO0556] [NCBI-GI:21219090] [NCBI-GeneID:1095979] [UniProt:Q9RJI3]
SCO1087	44	0.14136	-1.14	metabolic process, catalytic activity	2SCG4.03c; putative aldolase [EC:4.1.2.5]; K01620 threonine aldolase; [GeneDB:SCO1087] [NCBI-GI:21219602] [NCBI-GeneID:1096510] [UniProt:Q9K3R1]
SCO1088	48	0.13979	-1.14	metabolic process, catalytic activity	2SCG4.04c; oxidoreductase; [GeneDB:SCO1088] [NCBI-GI:21219603] [NCBI-GeneID:1096511] [UniProt:Q9K3R0]
SCO1089	12	0.07667	-1.46		2SCG4.05c; hypothetical protein 2SCG405c; [GeneDB:SCO1089] [NCBI-GI:21219604] [NCBI-GeneID:1096512] [UniProt:Q9K3Q9]
SCO1570	16	0.06500	-1.48	metabolic process, catalytic activity	argH. SCL24.06c; argininosuccinate lyase [EC:4.3.2.1]; K01755 argininosuccinate lyase; [GeneDB:SCO1570] [NCBI-GI:21220068] [NCBI-GeneID:1097001] [UniProt:Q9L1B1]
SCO1576	11	0.05636	-1.55	metabolic process, regulation of biological process, DNA binding	argR. SCL24.12c; arginine repressor; K03402 transcriptional regulator of arginine metabolism; [GeneDB:SCO1576] [NCBI-GI:21220074] [NCBI-GeneID:1097007] [UniProt:Q9L1A5]
SCO1577	10	0.05000	-1.57	metabolic process, catalytic activity	argD. SCL24.13c; acetonitrile aminotransferase [EC:2.6.1.11]; K00818 acetylornithine aminotransferase; [GeneDB:SCO1577] [NCBI-GI:21220075] [NCBI-GeneID:1097008] [UniProt:Q9L1A4]
SCO1578	8	0.01875	-1.72	metabolic process, catalytic activity, nucleotide binding	argB. SCL24.14c; acetylglutamate kinase [EC:2.7.2.8]; K00930 acetylglutamate kinase; [GeneDB:SCO1578] [NCBI-GI:21220076] [NCBI-GeneID:1097009] [UniProt:Q9L1A3]
SCO1579	29	0.06828	-1.40	metabolic process, catalytic activity	argJ. SCL24.15c; putative glutamate N-acetyltransferase [EC:2.3.1.1 2.3.1.35]; K00620 amino-acid N-acetyltransferase; K00642 glutamate N-acetyltransferase; [GeneDB:SCO1579] [NCBI-

					GI:32141148] [NCBI-GeneID:1097010] [UniProt:Q8CK24]
SCO1580	14	0.07143	-1.50	metabolic process, catalytic activity, nucleotide binding, protein binding	argC; N-acetyl-gamma-glutamyl-phosphate reductase [EC:1.2.1.38]; K00145 N-acetyl-gamma-glutamyl-phosphate reductase; [GeneDB:SCO1580] [NCBI-GI:21220077] [NCBI-GeneID:1097011] [UniProt:P54895]
SCO1815	45	0.14400	-1.21	metabolic process, catalytic activity, nucleotide binding	fabG. SCI28.09c; probable 3-oxacyl-(acyl-carrier-protein) reductase [EC:1.1.1.100]; K00059 3-oxoacyl-[acyl-carrier protein] reductase; [GeneDB:SCO1815] [NCBI-GI:21220305] [NCBI-GeneID:1097249] [UniProt:Q9S274]
SCO2296	30	0.06700	-1.69		SCC30.04; integral membrane protein; [GeneDB:SCO2296] [NCBI-GI:21220765] [NCBI-GeneID:1097730] [UniProt:Q9L017]
SCO2463	31	0.07194	-1.58	metabolic process, catalytic activity, nucleotide binding, transporter activity	SC7A8.02; putative ABC transporter; K06147 ATP-binding cassette. subfamily B. bacterial; [GeneDB:SCO2463] [NCBI-GI:21220926] [NCBI-GeneID:1097897] [UniProt:Q9L2F3]
SCO2464	27	0.06444	-1.67	metabolic process, catalytic activity, nucleotide binding, transporter activity	SC7A8.03; putative ABC transporter; K06147 ATP-binding cassette. subfamily B. bacterial; [GeneDB:SCO2464] [NCBI-GI:21220927] [NCBI-GeneID:1097898] [UniProt:Q9L2F2]
SCO2776	42	0.13548	-2.00	metabolic process, catalytic activity	accD1. SCC105.07; acetyl/propionyl CoA carboxylase. beta subunit [EC:6.4.1.3]; K01966 propionyl-CoA carboxylase beta chain; [GeneDB:SCO2776] [NCBI-GI:21221227] [NCBI-GeneID:1098210] [UniProt:Q9L077]
SCO2778	47	0.14277	-1.86	metabolic process, catalytic activity	hmgL. SCC105.09; hydroxymethylglutaryl-CoA lyase [EC:4.1.3.4]; K01640 hydroxymethylglutaryl-CoA lyase; [GeneDB:SCO2778] [NCBI-GI:21221229] [NCBI-GeneID:1098212] [UniProt:Q9L075]
SCO2779	9	0.04111	-2.71	metabolic process, catalytic activity, nucleotide binding	acdH. SCC105.10; acyl-CoA dehydrogenase; [GeneDB:SCO2779] [NCBI-GI:21221230] [NCBI-GeneID:1098213] [UniProt:Q9XCG6]
SCO3138	3	0.02333	-1.86	metabolic process, catalytic activity, metal ion binding	galT. SCE66.17c; galactose-1-phosphate uridylyltransferase [EC:2.7.7.10]; K00964 galactose-1-phosphate uridylyltransferase; [GeneDB:SCO3138] [NCBI-GI:21221576] [NCBI-GeneID:1098572] [UniProt:Q9K3S6]
SCO3411	49	0.14939	-1.30		SCE9.18c; possible membrane protein; K03975 membrane-associated protein; [GeneDB:SCO3411] [NCBI-GI:21221838] [NCBI-GeneID:1098848] [UniProt:Q9X8J1]
SCO3413	1	0.00000	-3.35	metabolic process, DNA binding, nucleotide binding	SCE9.20. tipA; transcriptional regulator; [GeneDB:SCO3413] [NCBI-GI:21221840] [NCBI-GeneID:1098850] [UniProt:P0A4T8]

SCO3472	2	0.00500	-2.22	metabolic process, DNA binding, catalytic activity	SCE65.08c; transposase remnant; [GeneDB:SCO3472] [NCBI-GI:21221896] [NCBI-GeneID:1098909] [UniProt:Q9RKG5]
SCO3473	34	0.07853	-1.23	metabolic process, catalytic activity	SCE65.09c; putative aldolase [EC:4.1.2.14 4.1.3.16]; K01625 2-dehydro-3-deoxyphosphogluconate aldolase; K01650 4-hydroxy-2-oxoglutarate aldolase; [GeneDB:SCO3473] [NCBI-GI:21221897] [NCBI-GeneID:1098910] [UniProt:Q9RKG4]
SCO3474	24	0.06917	-1.73	metabolic process, catalytic activity	SCE65.10c; sugar kinase; [GeneDB:SCO3474] [NCBI-GI:21221898] [NCBI-GeneID:1098911] [UniProt:Q9RKG3]
SCO3475	26	0.06615	-1.84	metabolic process, catalytic activity	SCE65.11c; putative galactonate dehydratase protein [EC:4.2.1.6]; K01684 galactonate dehydratase; [GeneDB:SCO3475] [NCBI-GI:21221899] [NCBI-GeneID:1098912] [UniProt:Q9RKG2]
SCO3476	25	0.06840	-1.77	metabolic process, catalytic activity	SCE65.12c; putative short-chain dehydrogenase [EC:1.1.1.125]; K00065 2-deoxy-D-gluconate 3-dehydrogenase; [GeneDB:SCO3476] [NCBI-GI:21221900] [NCBI-GeneID:1098913] [UniProt:Q9RKG1]
SCO3477	33	0.07273	-1.79	metabolic process, catalytic activity, metal ion binding	SCE65.13c; dehydrogenase; [GeneDB:SCO3477] [NCBI-GI:21221901] [NCBI-GeneID:1098914] [UniProt:Q9RKG0]
SCO3478	37	0.12432	-1.72	metabolic process, catalytic activity	SCE65.14c; dehydrogenase; [GeneDB:SCO3478] [NCBI-GI:21221902] [NCBI-GeneID:1098915] [UniProt:Q9RKF9]
SCO3480	43	0.14047	-1.45	metabolic process, catalytic activity	SCE65.16c; putative racemase [EC:5.1.2.2]; K01781 mandelate racemase; [GeneDB:SCO3480] [NCBI-GI:21221904] [NCBI-GeneID:1098917] [UniProt:Q9RKF7]
SCO3481	40	0.12925	-1.36	metabolic process, catalytic activity	SCE65.17c; hypothetical protein; [GeneDB:SCO3481] [NCBI-GI:21221905] [NCBI-GeneID:1098918] [UniProt:Q9RKF6]
SCO3483	13	0.07615	-1.70	transporter activity	SCE65.19c; putative integral membrane transport protein; K02025 multiple sugar transport system permease protein; [GeneDB:SCO3483] [NCBI-GI:21221907] [NCBI-GeneID:1098920] [UniProt:Q9RKF4]
SCO3484	23	0.07174	-1.55		SCE65.20c; putative secreted sugar-binding protein; K02027 multiple sugar transport system substrate-binding protein; [GeneDB:SCO3484] [NCBI-GI:21221908] [NCBI-GeneID:1098921] [UniProt:Q9RKF3]
SCO3487	17	0.06353	-1.74	metabolic process, catalytic activity	SCE65.23; hydrolase; [GeneDB:SCO3487] [NCBI-GI:21221911] [NCBI-GeneID:1098924] [UniProt:Q9RKF0]
SCO5025	5	0.01400	-3.16	metabolic process, regulation of biological process, DNA binding,	SCK15.27; transcriptional regulator; [GeneDB:SCO5025] [NCBI-GI:21223398] [NCBI-GeneID:1100466] [UniProt:Q9KY77]

				nucleotide binding	
SCO5026	21	0.06286	-2.49	metabolic process, catalytic activity	SCK15.28; hypothetical protein; [GeneDB:SCO5026] [NCBI-GI:21223399] [NCBI-GeneID:1100467] [UniProt:Q9KY76]
SCO5367	39	0.13205	-0.97		2SC6G5.11. atpB; ATP synthase A chain [EC:3.6.3.14]; K02108 F-type H+-transporting ATPase a chain; [GeneDB:SCO5367] [NCBI-GI:21223727] [NCBI-GeneID:1100807] [UniProt:Q9K4D8]
SCO5536	18	0.06111	-1.59		SC1C2.17; hypothetical protein; [GeneDB:SCO5536] [NCBI-GI:21223891] [NCBI-GeneID:1100976] [UniProt:O86518]
SCO5676	35	0.08829	-1.27	metabolic process, catalytic activity	gabT; putative 4-aminobutyrate aminotransferase [EC:2.6.1.19]; K00823 4-aminobutyrate aminotransferase; [GeneDB:SCO5676] [NCBI-GI:21224026] [NCBI-GeneID:1101115] [UniProt:O86823]
SCO5839	46	0.14217	-1.16		SC9B10.06; hypothetical protein; [GeneDB:SCO5839] [NCBI-GI:21224183] [NCBI-GeneID:1101281] [UniProt:O50513]
SCO5976	32	0.06969	-1.34		arcB. StBAC16H6.11; ornithine carbamoyltransferase [EC:2.1.3.3]; K00611 ornithine carbamoyltransferase; [GeneDB:SCO5976] [NCBI-GI:21224313] [NCBI-GeneID:1101418] [UniProt:Q93JF1]
SCO6164	36	0.10833	-1.51		SC1A9.28c; putative DnaK suppressor protein; K06204 DnaK suppressor protein; [GeneDB:SCO6164] [NCBI-GI:21224490] [NCBI-GeneID:1101605] [UniProt:Q9ZBS4]
SCO6266	20	0.06350	-2.00		SCAH10.31. scbA; ScbA protein; [GeneDB:SCO6266] [NCBI-GI:21224587] [NCBI-GeneID:1101707] [UniProt:O86851]
SCO7036	4	0.01750	-1.84	metabolic process, catalytic activity, nucleotide binding	argG. SC4G1.02; argininosuccinate synthase [EC:6.3.4.5]; K01940 argininosuccinate synthase; [GeneDB:SCO7036] [NCBI-GI:21225319] [NCBI-GeneID:1102474] [UniProt:P24532]
SCO7262	22	0.07364	-1.51		SC5H1.30c; hypothetical protein; [GeneDB:SCO7262] [NCBI-GI:21225539] [NCBI-GeneID:1102700] [UniProt:Q9X7T7]
SCO7530	41	0.13220	-1.69	metabolic process, regulation of biological process, DNA binding, nucleotide binding	SC8G12.06c; regulatory protein; [GeneDB:SCO7530] [NCBI-GI:21225795] [NCBI-GeneID:1102968] [UniProt:Q9KYZ9]
SCO7586	7	0.02143	-2.78	metabolic process, catalytic activity, nucleotide binding	SC5F1.40; oxidoreductase; [GeneDB:SCO7586] [NCBI-GI:21225850] [NCBI-GeneID:1103024] [UniProt:Q9F389]
SCO7587	38	0.12237	-1.39		SC5F1.41; integral membrane protein; [GeneDB:SCO7587] [NCBI-GI:21225851] [NCBI-GeneID:1103025] [UniProt:Q9F388]

SCO7698	19	0.06579	-2.39	metabolic process, regulation of biological process, DNA binding, nucleotide binding	SC1A4.06c; MerR-family transcriptional regulator; [GeneDB:SCO7698] [NCBI-GI:21225958] [NCBI-GeneID:1103136] [UniProt:Q9EX58]
**Gene name**	**Rank of up-regulated genes**	**pfp value**	**Log(2)**	**GO Biological Process / Molecular Function**	**Product/Function**
SCO0016	68	0.10735	2.28		SCJ30.12; hypothetical protein; [GeneDB:SCO0016] [NCBI-GI:21218598] [NCBI-GeneID:1095450] [UniProt:Q9S1X7]
SCO0090	58	0.08345	1.62		SCJ11.19c; transposase; [GeneDB:SCO0090] [NCBI-GI:21218659] [NCBI-GeneID:1095518] [UniProt:Q9RI85]
SCO0489	36	0.04861	1.43		SCF34.08c; hypothetical protein; K05375 MbtH protein; [GeneDB:SCO0489] [NCBI-GI:21219027] [NCBI-GeneID:1095912] [UniProt:Q9RK17]
SCO0498	41	0.05122	1.27	metabolic process, catalytic activity	SCF34.17c; putative peptide monooxygenase [EC:1.13.12.-]; K00468; [GeneDB:SCO0498] [NCBI-GI:21219036] [NCBI-GeneID:1095921] [UniProt:Q9RK08]
SCO0861	65	0.09815	1.90		SCM2.14c; secreted protein; [GeneDB:SCO0861] [NCBI-GI:21219382] [NCBI-GeneID:1096284] [UniProt:Q9RCV0]
SCO0863	84	0.15250	1.64		SCM2.16c; integral membrane protein; [GeneDB:SCO0863] [NCBI-GI:21219384] [NCBI-GeneID:1096286] [UniProt:Q9RCU8]
SCO0973	66	0.09970	1.67	transporter activity	SCM11.28; integral membrane protein; [GeneDB:SCO0973] [NCBI-GI:21219491] [NCBI-GeneID:1096396] [UniProt:Q9RIT4]
SCO1550	69	0.10696	1.34		SCL11.06c; small membrane protein; [GeneDB:SCO1550] [NCBI-GI:21220049] [NCBI-GeneID:1096981] [UniProt:Q9L1D2]
SCO1713	45	0.05422	1.80		SCI11.02c; hypothetical protein; [GeneDB:SCO1713] [NCBI-GI:21220207] [NCBI-GeneID:1097144] [UniProt:Q9S2B7]
SCO2126	74	0.10730	2.03	metabolic process, catalytic activity, nucleotide binding	glk. SC6E10.20c; glucokinase [EC:2.7.1.2]; K00845 glucokinase; [GeneDB:SCO2126] [NCBI-GI:21220604] [NCBI-GeneID:1097560] [UniProt:P0A4E1]
SCO2306	83	0.14361	1.68	transporter activity, catalytic activity, nucleotide binding	SCC30.14; putative ABC transporter integral membrane protein; K01992 ABC-2 type transport system permease protein; [GeneDB:SCO2306] [NCBI-GI:21220775] [NCBI-GeneID:1097740] [UniProt:Q9L007]

SCO2513	35	0.04714	1.81		SCC121.16; hypothetical protein; [GeneDB:SCO2513] [NCBI-GI:21220974] [NCBI-GeneID:1097947] [UniProt:Q9L2H0]
SCO2528	72	0.10667	1.86	metabolic process, catalytic activity,	leuA. SCC121.31c; 2-ispoprylmalate synthase [EC:2.3.3.13]; K01649 2-isopropylmalate synthase; [GeneDB:SCO2528] [NCBI-GI:32141173] [NCBI-GeneID:1097962] [UniProt:O31046]
SCO2839	37	0.04784	1.81		SCE20.13c; lipoprotein; [GeneDB:SCO2839] [NCBI-GI:21221289] [NCBI-GeneID:1098273] [UniProt:Q9RDB2]
SCO2930	59	0.08763	1.53	transporter activity	SCE19A.30; putative permease membrane component; K05846 osmoprotectant transport system permease protein; [GeneDB:SCO2930] [NCBI-GI:21221377] [NCBI-GeneID:1098363] [UniProt:Q9S2F1]
SCO2986	47	0.05681	1.72	response to stimulus	SCE50.14c; hypothetical protein; [GeneDB:SCO2986] [NCBI-GI:21221430] [NCBI-GeneID:1098419] [UniProt:Q9L049]
SCO3089	34	0.04588	2.05		SCE25.30; putative ABC transporter ATP-binding protein; K02003; [GeneDB:SCO3089] [NCBI-GI:21221529] [NCBI-GeneID:1098523] [UniProt:Q9KZ60]
SCO3090	56	0.08179	1.62		SCE25.31; putative ABC transporter integral membrane protein; K02004; [GeneDB:SCO3090] [NCBI-GI:21221530] [NCBI-GeneID:1098524] [UniProt:Q9KZ59]
SCO3111	43	0.05395	1.87	metabolic process, catalytic activity, nucleotide binding	SCE41.20c; putative ABC transport system ATP-binding protein; K02003; [GeneDB:SCO3111] [NCBI-GI:21221550] [NCBI-GeneID:1098545] [UniProt:Q9F2N9]
SCO3152	39	0.05154	2.43		SCE87.03c; hypothetical protein; [GeneDB:SCO3152] [NCBI-GI:21221589] [NCBI-GeneID:1098586] [UniProt:Q9RKD5]
SCO3263	16	0.01938	3.25		SCE39.13c; hypothetical protein; [GeneDB:SCO3263] [NCBI-GI:21221696] [NCBI-GeneID:1098697] [UniProt:Q9X8D6]
SCO3264	38	0.05079	2.50	metabolic process, regulation of biological process, DNA binding	SCE39.14c; GntR-family regulator; [GeneDB:SCO3264] [NCBI-GI:21221697] [NCBI-GeneID:1098698] [UniProt:Q9X8D7]
SCO3265	7	0.00571	3.46		SCE39.15c; hypothetical protein; [GeneDB:SCO3265] [NCBI-GI:21221698] [NCBI-GeneID:1098699] [UniProt:Q9X8D8]
SCO3266	26	0.03615	2.77	metabolic process, catalytic activity, transporter activity	SCE39.16c; hypothetical protein; [GeneDB:SCO3266] [NCBI-GI:21221699] [NCBI-GeneID:1098700] [UniProt:Q9X8D9]
SCO3268	2	0.00500	3.74		SCE39.18c; secreted protein; [GeneDB:SCO3268] [NCBI-GI:21221701] [NCBI-GeneID:1098702] [UniProt:Q9X8E1]
SCO3390	77	0.12857	1.58	metabolic process, catalytic activity,	SCE126.08c; two component sensor kinase; [GeneDB:SCO3390] [NCBI-GI:21221818] [NCBI-GeneID:1098827] [UniProt:Q9X851]

				nucleotide binding, receptor activity	
SCO3717	49	0.06327	1.65	metabolic process, catalytic activity, transporter activity	SCH35.07; putative cation transport system component [EC:3.6.3.12]; K01547 K+-transporting ATPase ATPase B chain; [GeneDB:SCO3717] [NCBI-GI:21222130] [NCBI-GeneID:1099153] [UniProt:Q9X8Z9]
SCO3719	23	0.03478	2.20	metabolic process, catalytic activity, transporter activity	SCH35.05; small membrane protein; [GeneDB:SCO3719] [NCBI-GI:21222132] [NCBI-GeneID:1099155] [UniProt:Q9X901]
SCO3918	55	0.08309	1.59	metabolic process, catalytic activity	SCH24.40c. SCQ11.01c; hypothetical protein; [GeneDB:SCO3918] [NCBI-GI:32141220] [NCBI-GeneID:1099354] [UniProt:Q8CJV3]
SCO3956	19	0.02526	2.02	metabolic process, catalytic activity, nucleotide binding	SCD78.23; putative ABC transporter ATP-binding protein; K01990 ABC-2 type transport system ATP-binding protein; [GeneDB:SCO3956] [NCBI-GI:21222361] [NCBI-GeneID:1099392] [UniProt:Q9ZBX6]
SCO3957	32	0.04281	1.79		SCD78.24; possible integral membrane protein; K01992 ABC-2 type transport system permease protein; [GeneDB:SCO3957] [NCBI-GI:21222362] [NCBI-GeneID:1099393] [UniProt:Q9ZBX5]
SCO3958	42	0.05024	1.61	metabolic process, catalytic activity,, nucleotide binding	SCD78.25; ABC transporter ATP-binding protein; K01990 ABC-2 type transport system ATP-binding protein; [GeneDB:SCO3958] [NCBI-GI:21222363] [NCBI-GeneID:1099394] [UniProt:Q9ZBX4]
SCO3959	61	0.08885	1.55		SCD78.26; possible integral membrane protein; K01992 ABC-2 type transport system permease protein; [GeneDB:SCO3959] [NCBI-GI:21222364] [NCBI-GeneID:1099395] [UniProt:Q9ZBX3]
SCO3967	52	0.07750	1.67		SCBAC25E3.04c; conserved hypothetical membrane protein; K07040; [GeneDB:SCO3967] [NCBI-GI:21222371] [NCBI-GeneID:1099403] [UniProt:Q93J39]
SCO3982	3	0.00333	3.96		SCBAC25E3.19; hypothetical protein; [GeneDB:SCO3982] [NCBI-GI:21222386] [NCBI-GeneID:1099418] [UniProt:Q93J25]
SCO3983	15	0.01533	3.48		SCBAC25E3.20; hypothetical protein; [GeneDB:SCO3983] [NCBI-GI:21222387] [NCBI-GeneID:1099419] [UniProt:Q93J24]
SCO3985	48	0.06229	2.29		SCBAC25E3.22; hypothetical protein; [GeneDB:SCO3985] [NCBI-GI:21222389] [NCBI-GeneID:1099421] [UniProt:Q93J22]
SCO3986	8	0.00750	3.77	metabolic process, regulation of biological process, DNA binding	SCBAC25E3.23; GntR-family transcriptional regulator; [GeneDB:SCO3986] [NCBI-GI:21222390] [NCBI-GeneID:1099422] [UniProt:Q93J21]
SCO3987	14	0.01571	3.36		SCBAC25E3.24; hypothetical protein; [GeneDB:SCO3987] [NCBI-GI:21222391] [NCBI-GeneID:1099423] [UniProt:Q93J20]
SCO3988	30	0.04467	2.47		SCBAC25E3.25; hypothetical protein; [GeneDB:SCO3988] [NCBI-GI:21222392] [NCBI-

					GeneID:1099424] [UniProt:Q93J19]
SCO4004	6	0.00500	2.99		2SC10A7.08c; small membrane protein; [GeneDB:SCO4004] [NCBI-GI:21222407] [NCBI-GeneID:1099440] [UniProt:Q9ADQ1]
SCO4005	13	0.01538	2.90	metabolic process, regulation of biological process, DNA binding	2SC10A7.09; RNA polymerase sigma factor; [GeneDB:SCO4005] [NCBI-GI:21222408] [NCBI-GeneID:1099441] [UniProt:Q9ADQ0]
SCO4054	75	0.11027	1.45		2SCD60.20; integral membrane protein; [GeneDB:SCO4054] [NCBI-GI:21222457] [NCBI-GeneID:1099490] [UniProt:Q9AK63]
SCO4173	70	0.10857	1.87		SCD66.10c; hypothetical protein; [GeneDB:SCO4173] [NCBI-GI:21222570] [NCBI-GeneID:1099613] [UniProt:Q9K4G5]
SCO4174	11	0.01455	3.70		SCD66.11c; integral membrane protein; [GeneDB:SCO4174] [NCBI-GI:21222571] [NCBI-GeneID:1099614] [UniProt:Q9K4G4]
SCO4175	79	0.13608	1.75		SCD66.12c; hypothetical protein; [GeneDB:SCO4175] [NCBI-GI:21222572] [NCBI-GeneID:1099615] [UniProt:Q9K4G3]
SCO4261	54	0.08111	1.56	metabolic process, regulation of biological process, DNA binding	SCD49.02c; response regulator; [GeneDB:SCO4261] [NCBI-GI:21222655] [NCBI-GeneID:1099701] [UniProt:Q9K4F1]
SCO4293	78	0.13269	1.74	metabolic process, catalytic activity	SCD95A.26; putative threonine synthase [EC:4.2.3.1]; K01733 threonine synthase; [GeneDB:SCO4293] [NCBI-GI:21222686] [NCBI-GeneID:1099733] [UniProt:Q9KXU8]
SCO4317	53	0.07717	1.79		SCD95A.50; hypothetical protein; [GeneDB:SCO4317] [NCBI-GI:21222710] [NCBI-GeneID:1099757] [UniProt:Q9KXS4]
SCO4368	57	0.08368	1.77	metabolic process, catalytic activity	SCD19.23c; lipase (secreted protein); [GeneDB:SCO4368] [NCBI-GI:21222759] [NCBI-GeneID:1099808] [UniProt:Q9F2X9]
SCO4851	18	0.02111	2.19		SC5G8.19c; hypothetical protein; [GeneDB:SCO4851] [NCBI-GI:21223227] [NCBI-GeneID:1100292] [UniProt:Q9KZ95]
SCO4852	5	0.00600	2.77		SC5G8.20c; integral membrane protein; [GeneDB:SCO4852] [NCBI-GI:21223228] [NCBI-GeneID:1100293] [UniProt:Q9KZ94]
SCO4903	31	0.04323	1.98		2SCK8.29c; hypothetical protein; [GeneDB:SCO4903] [NCBI-GI:21223277] [NCBI-GeneID:1100344] [UniProt:Q9AK23]
SCO4947	80	0.13813	1.23	metabolic process, catalytic activity, metal ion binding	2SCK31.07. narG3; nitrate reductase alpha chain NarG3 [EC:1.7.99.4]; K00370 nitrate reductase 1. alpha subunit; [GeneDB:SCO4947] [NCBI-GI:21223320] [NCBI-GeneID:1100388] [UniProt:Q9EWF3]

SCO4950	85	0.15294	1.24	metabolic process, catalytic activity	2SCK31.10. narI3; nitrate reductase gamma chain NarI3; [GeneDB:SCO4950] [NCBI-GI:21223323] [NCBI-GeneID:1100391] [UniProt:Q9EWF6]
SCO5285	73	0.10562	1.50	metabolic process, response to stimulus, DNA binding, catalytic activity	lon. SCCB12.09; ATP-dependent protease [EC:3.4.21.53]; K01338 ATP-dependent Lon protease; [GeneDB:SCO5285] [NCBI-GI:21223651] [NCBI-GeneID:1100726] [UniProt:Q9EVK2]
SCO5521	1	0.01000	3.17		SC1C2.02; hypothetical protein; [GeneDB:SCO5521] [NCBI-GI:21223876] [NCBI-GeneID:1100961] [UniProt:O86503]
SCO5774	33	0.04273	2.04	transporter activity	gluD; glutamate permease; K10007 glutamate transport system permease protein; [GeneDB:SCO5774] [NCBI-GI:21224120] [NCBI-GeneID:1101216] [UniProt:O50492]
SCO5775	63	0.08746	1.68	transporter activity	gluC; glutamate permease; K10006 glutamate transport system permease protein; [GeneDB:SCO5775] [NCBI-GI:21224121] [NCBI-GeneID:1101217] [UniProt:O50493]
SCO5776	20	0.02800	2.18	transporter activity	gluB; glutamate binding protein; K10005 glutamate transport system substrate-binding protein; [GeneDB:SCO5776] [NCBI-GI:21224122] [NCBI-GeneID:1101218] [UniProt:O50494]
SCO5777	62	0.08742	1.77	metabolic process, catalytic activity, nucleotide binding	gluA; glutamate uptake system ATP-binding protein [EC:3.6.3.-]; K10008 glutamate transport system ATP-binding protein; [GeneDB:SCO5777] [NCBI-GI:21224123] [NCBI-GeneID:1101219] [UniProt:O50495]
SCO6268	64	0.09375	1.56	cell communication, metabolic process, regulation of biological process, nucleotide binding	SCAH10.33c; histidine kinase; [GeneDB:SCO6268] [NCBI-GI:21224589] [NCBI-GeneID:1101709] [UniProt:Q9RKS6]
SCO6272	21	0.02667	1.66	metabolic process, catalytic activity, nucleotide binding	SC2C4.02; secreted FAD-binding protein; [GeneDB:SCO6272] [NCBI-GI:21224592] [NCBI-GeneID:1101713] [UniProt:Q9EX55]
SCO6273	46	0.05609	1.70	metabolic process, catalytic activity	SC2C4.03c; type I polyketide synthase; [GeneDB:SCO6273] [NCBI-GI:21224593] [NCBI-GeneID:1101714] [UniProt:Q9EX54]
SCO6274	44	0.05545	1.59	metabolic process, catalytic activity	SC2C4.04c; type I polyketide synthase; [GeneDB:SCO6274] [NCBI-GI:21224594] [NCBI-GeneID:1101715] [UniProt:Q9EX53]
SCO6275	67	0.10746	1.41	metabolic process, catalytic activity	SC1G7.01c. SC2C4.05c; type I polyketide synthase; [GeneDB:SCO6275] [NCBI-GI:32141295] [NCBI-GeneID:1101716] [UniProt:Q8CJN6]
SCO6276	17	0.02118	1.57		SC1G7.02; secreted protein; [GeneDB:SCO6276] [NCBI-GI:21224595] [NCBI-GeneID:1101717] [UniProt:Q93S13]

SCO6277	27	0.03481	1.47	metabolic process, catalytic activity	SC1G7.03; epoxide hydrolase; [GeneDB:SCO6277] [NCBI-GI:21224596] [NCBI-GeneID:1101718] [UniProt:Q93S12]
SCO6278	24	0.03458	1.94	transport	SC1G7.04; putative integral membrane transport protein; K08167 MFS transporter. DHA2 family. methyl viologen resistance protein SmvA; [GeneDB:SCO6278] [NCBI-GI:21224597] [NCBI-GeneID:1101719] [UniProt:Q93S11]
SCO6279	12	0.01333	1.85	metabolic process, catalytic activity	SC1G7.05; diaminobutyrate-pyruvate aminotransferase; [GeneDB:SCO6279] [NCBI-GI:21224598] [NCBI-GeneID:1101720] [UniProt:Q93S10]
SCO6282	9	0.01000	2.19	metabolic process, catalytic activity	SC1G7.08c; 3-oxoacyl-[acyl-carrier protein] reductase; [GeneDB:SCO6282] [NCBI-GI:21224601] [NCBI-GeneID:1101723] [UniProt:Q93S07]
SCO6283	50	0.06740	1.44		SC1G7.09; hypothetical protein; [GeneDB:SCO6283] [NCBI-GI:21224602] [NCBI-GeneID:1101724] [UniProt:Q93S06]
SCO6285	82	0.14220	1.52		SC1G7.11; hypothetical protein; [GeneDB:SCO6285] [NCBI-GI:21224604] [NCBI-GeneID:1101726] [UniProt:Q93S04]
SCO6288	81	0.13901	1.00	cell communication, metabolic process, regulation of biological process, DNA binding, signal transducer activity	SC1G7.14. SCBAC8D1.01; regulatory protein; [GeneDB:SCO6288] [NCBI-GI:21224607] [NCBI-GeneID:1101729] [UniProt:Q93RY4]
SCO6429	25	0.03600	1.94		SC1A6.18; hypothetical protein; [GeneDB:SCO6429] [NCBI-GI:21224736] [NCBI-GeneID:1101868] [UniProt:O69823]
SCO6431	29	0.04379	1.85	metabolic process, catalytic activity	SC1A6.20; peptide synthase; [GeneDB:SCO6431] [NCBI-GI:21224738] [NCBI-GeneID:1101870] [UniProt:O69825]
SCO6433	10	0.00900	2.37		SC1A6.22; hypothetical protein; K06995; [GeneDB:SCO6433] [NCBI-GI:21224740] [NCBI-GeneID:1101872] [UniProt:O69827]
SCO6434	51	0.07765	1.63	metabolic process, catalytic activity	SC1A6.23. SC9B5.01; oxidoreductase; [GeneDB:SCO6434] [NCBI-GI:32141301] [NCBI-GeneID:1101873] [UniProt:Q8CJN0]
SCO6435	22	0.02591	2.00	metabolic process, regulation of biological process	SC9B5.02; hypothetical protein; [GeneDB:SCO6435] [NCBI-GI:21224741] [NCBI-GeneID:1101874] [UniProt:Q9ZBH8]
SCO6436	28	0.03893	1.96	metabolic process, catalytic activity, nucleotide binding	SC9B5.03; tRNA synthetase; [GeneDB:SCO6436] [NCBI-GI:21224742] [NCBI-GeneID:1101875] [UniProt:Q9ZBH7]

SCO6509	60	0.08633	1.88		SC1E6.18c; hydrophobic protein; [GeneDB:SCO6509] [NCBI-GI:21224812] [NCBI-GeneID:1101948] [UniProt:Q9ZC04]
SCO7399	76	0.12987	1.20		SC10G8.27c; binding-protein-dependent transport lipoprotein; [GeneDB:SCO7399] [NCBI-GI:21225670] [NCBI-GeneID:1102837] [UniProt:Q9L178]
SCO7643	71	0.10789	1.36		SC10F4.16; hypothetical protein; [GeneDB:SCO7643] [NCBI-GI:21225904] [NCBI-GeneID:1103081] [UniProt:Q9F3P4]
SCO7676	4	0.00500	3.17	metal ion binding	SC4C2.11; ferredoxin; [GeneDB:SCO7676] [NCBI-GI:21225936] [NCBI-GeneID:1103114] [UniProt:Q9EWQ1]
SCO7799	40	0.05200	1.92		SC10B8A.09c; transposase; [GeneDB:SCO7799] [NCBI-GI:21226051] [NCBI-GeneID:1103237] [UniProt:Q9FCL5]

Table S3. Differentially expressed genes shared between Glc/Agar and *Sco*M145/*ScoZm* comparisons

Gene name	Product	Function
SCO0090		Transposase
SCO0489		Hypothetical protein
SCO0498		Putative peptide monooxygenase [EC:1.13.12.-]
SCO0861		Secreted protein
SCO0863		Integral membrane protein
SCO0973		Integral membrane protein
SCO1087		Putative aldolase [EC:4.1.2.5]
SCO1088		Oxidoreductase
SCO1089		Hypothetical protein 2SCG405c
SCO1550		Small membrane protein
SCO1713		Hypothetical protein
SCO2306		Putative ABC transporter integral membrane protein
SCO2463		Putative ABC transporter
SCO2464		Putative ABC transporter
SCO2528	leuA. SCC121.31c	2-Ispoprylmalate synthase [EC:2.3.3.13]
SCO2776	accD1. SCC105.07	Acetyl/propionyl CoA carboxylase. beta subunit [EC:6.4.1.3]
SCO2778	hmgL. SCC105.09	Hydroxymethylglutaryl-CoA lyase [EC:4.1.3.4]
SCO2779	acdH. SCC105.10	Acyl-CoA dehydrogenase
SCO2839		Lipoprotein
SCO3089		Putative ABC transporter ATP-binding protein
SCO3090		Putative ABC transporter integral membrane protein

SCO3111		Putative ABC transport system ATP-binding protein
SCO3152		Hypothetical protein
SCO3263		Hypothetical protein
SCO3265		Hypothetical protein
SCO3266		Hypothetical protein
SCO3268		Secreted protein
SCO3390		Two component sensor kinase
SCO3472		Transposase remnant
SCO3473		Putative aldolase [EC:4.1.2.14 4.1.3.16]
SCO3474		Sugar kinase
SCO3475		Putative galactonate dehydratase protein [EC:4.2.1.6]
SCO3476		Putative short-chain dehydrogenase [EC:1.1.1.125]
SCO3477		Dehydrogenase
SCO3478		Dehydrogenase
SCO3480		Putative racemase [EC:5.1.2.2]
SCO3481		Hypothetical protein
SCO3483		Putative integral membrane transport protein
SCO3484		Putative secreted sugar-binding protein
SCO3487		Hydrolase
SCO3717		Putative cation transport system component [EC:3.6.3.12]
SCO3719		Small membrane protein
SCO3918		Hypothetical protein
SCO3956		Putative ABC transporter ATP-binding protein
SCO3957		Possible integral membrane protein
SCO3958		ABC transporter ATP-binding protein
SCO3959		Possible integral membrane protein

SCO3967		Conserved hypothetical membrane protein
SCO3982		Hypothetical protein
SCO3983		Hypothetical protein
SCO3986		GntR-family transcriptional regulator
SCO3987		Hypothetical protein
SCO3988		Hypothetical protein
SCO4005		RNA polymerase sigma factor
SCO4054		Integral membrane protein
SCO4261		Response regulator
SCO4293		Putative threonine synthase [EC:4.2.3.1]
SCO4368		Lipase (secreted protein)
SCO4851		Hypothetical protein
SCO4852		Integral membrane protein
SCO4947		Nitrate reductase alpha chain NarG3 [EC:1.7.99.4]
SCO5285		ATP-dependent protease [EC:3.4.21.53]
SCO5521		Hypothetical protein
SCO5676	gabT	Putative 4-aminobutyrate aminotransferase [EC:2.6.1.19]
SCO5774	gluD	Glutamate permease
SCO5775	gluC	Glutamate permease
SCO5776	gluB	Glutamate binding protein
SCO5777	gluA	Glutamate uptake system ATP-binding protein [EC:3.6.3.-]
SCO6164		Putative DnaK suppressor protein
SCO6272		Secreted FAD-binding protein
SCO6273		Type I polyketide synthase
SCO6274		Type I polyketide synthase
SCO6275		Type I polyketide synthase

SCO6276		Secreted protein
SCO6277		Epoxide hydrolase
SCO6278		Putative integral membrane transport protein
SCO6279		Diaminobutyrate-pyruvate aminotransferase
SCO6282		3-Oxoacyl-[acyl-carrier protein] reductase
SCO6283		Hypothetical protein
SCO6285		Hypothetical protein
SCO6429		Hypothetical protein
SCO6431		Peptide synthase
SCO6433		Hypothetical protein
SCO6434		Oxidoreductase
SCO6435		Hypothetical protein
SCO6436		tRNA synthetase
SCO6509		Hydrophobic protein
SCO7399		Binding-protein-dependent transport lipoprotein
SCO7643		Hypothetical protein
SCO7676		Ferredoxin
SCO7799		Transposase

Table S4. Oligonucleotides used in this study

**Name**	**Target**	**Sequence 5´-3´**
glk fwd-large		ttagcggggctcgaccgaactgagggattcatgggactcattccggggatccgtcgacc
glk rev-large		cgggtgagcgcagctcccggagtcgggaagtgatcgtcatgtaggctggagctgcttc
glk out-fwd		gtcgaaccacaggaacgccgctggaccac
glk out-rev		gagcgccgggagggatgttgccatgccgtcaa
*glk*1	*glk*	[23]
*glk*2	*glk*	[23]
SCO5373 fwd qpcr	*atpd*	tggtcttcggtcagatgga
SCO5373 rev qpcr	*atpd*	cggaagatgttgtcgatgaaga
SCO3878 fwd qpcr	*dnan*	cttcaacccgaccttcctg
SCO3878 rev qpcr	*dnan*	aggtacttgtaggcctcgt
SCO5769 fwd qpcr	*reca*	ggagatcgagggcgagat
SCO5769 rev qpcr	*reca*	gtggtcttggactggttgag
SCO6008 fwd qpcr	*rok7b7*	cgaaatcgggcacatcac
SCO6008 rev qpcr	*rok7b7*	aggagcggaagcacatag

SCO6162 fwd qpcr	*SCO6162*	atcgccgagatcctcttcat
SCO6162 rev qpcr	*SCO6162*	gtaccgggtcagctcca
SCO5578 fwd qpcr	*glcp*	gcagttcgtcggcatcaa
SCO5578 rev qpcr	*glcp*	cgtcgtgaacgagtagaagaac
SCO6164 fwd qpcr	*SCO6164*	gcgtcacccagcttcag
SCO6164 rev qpcr	*SCO6164*	tcgtcgatctccttgagca
SCO3471 fwd qpcr	*daga*	ttcactgaactggcgagaag
SCO3471 fwd qpcr	*daga*	tggaatccattccgaagaagag
SCO4729 fwd qpcr	*rpoa*	aagggcaagctggagatg
SCO4729 rev qpcr	*rpoa*	tgagaaccggcgagtaga
SCO3873 fwd qpcr	*gyra*	gcgaagatcaccgagtacaac
SCO3873 rev qpcr	*gyra*	tacgggatcagcttggtctt
SCO5820 fwd qpcr	*hrdb*	ggagaagttcgactacaccaag
SCO5820 rev qpcr	*hrdb*	ccatgtgcaccgggatac
